# Whole genome sequencing in cerebral palsy: a UK paediatric pilot study

**DOI:** 10.1016/j.lanepe.2026.101786

**Published:** 2026-07-30

**Authors:** Thiloka E. Ratnaike, Heather H. Pierce, Alison J. Coffey, Joao M.L. Dias, Iain R.L. Kean, Emily Li, Ravi P. More, Dulika S. Sumathipala, Maya Bajracharya, Zoya Kingsbury, Taksina Newington, Anthony S. Rogers, Isabelle Delon, Kate Downes, Yasmin de Alwis, Anna P. Basu, Manal Issa, Catherine Kearney, Amjad H. Khan, Benjamin P. Marlow, Alison Sansome, JooWook Ahn, A Yee Than, Tracy Lau, David R. Bentley, Sarah Bowdin, Evan Reid, Henry Houlden, Sean Humphray, David H. Rowitch

**Affiliations:** a Department of Paediatrics, University of Cambridge, Cambridge, UK; b Paediatric Neurology, Bristol Royal Hospital for Children, Upper Maudlin St, Bristol, UK; c School of Physiology, Pharmacology and Neuroscience, University of Bristol, Bristol, UK; d Illumina Inc., Illumina Centre, 19 Granta Park, Great Abington, Cambridge, UK; e East Genomic Medicine Service Alliance, Cambridge University Hospitals NHS Foundation Trust, Cambridge, UK; f Division of Immunology and Infectious Diseases, Australian National University, Australia; g East Genomic Laboratory Hub, Level 6 Addenbrookes Treatment Centre, Addenbrooke's Hospital, Cambridge, UK; h Paediatric Neurology, Royal Victoria Infirmary, Newcastle Upon Tyne, UK; i Population Health Sciences Institute, Newcastle University, Newcastle upon Tyne, UK; j Department of Paediatrics, Colchester Hospital, Turner Rd, Colchester, UK; k Redgrave Children and Young People's Centre Luton, UK; l Child Development Centre, Kempston Bedford, UK; m Child Development Centre, Addenbrookes Hospital, Cambridge, UK; n Department of Neuromuscular Diseases, UCL Queen Square Institute of Neurology, University College London, London, UK; o Cambridge Institute for Medical Research and Department of Genomic Medicine, The Keith Peters Building, Cambridge Biomedical Campus, Hills Road, Cambridge, UK; p Cedars-Sinai Guerin Children's, Departments of Paediatrics and Neurosurgery, Los Angeles, USA

**Keywords:** Cerebral palsy, Whole genome sequencing, Monogenic conditions, Genetic testing, National health service

## Abstract

**Background:**

Prior international studies indicate that 9–36% of people with cerebral palsy (CP) have a monogenic condition. However, the utility of whole genome sequencing (WGS) as a diagnostic tool for United Kingdom (UK) National Health Service (NHS) patients has not been evaluated.

**Methods:**

This prospective pilot study recruited 86 individuals with CP from specialist clinics in Bedford, Cambridge, Colchester, Newcastle, and Luton NHS Foundation Trusts. Gene-agnostic trio WGS was performed using AI-based variant prioritisation, with subsequent application of a CP gene list. Candidate diagnostic pathogenic (P) or likely pathogenic (LP) variants were reviewed at multidisciplinary meetings and confirmed in an NHS Genomic Laboratory Hub prior to issuing a clinical report. The use of human phenotype ontology (HPO) terms was evaluated to estimate probability of a diagnostic variant using a supervised linear discriminant analysis (PCA + LDA) model.

**Findings:**

86/157 (54.7%) individuals approached consented to the study. Variants meeting P/LP diagnostic criteria were identified in 11/86 cases (12.8%). 8/86 participants (9.3%) carried variants strongly suggestive of disease causation. Variants of uncertain significance were identified in 27/86 cases (31.4%). In all cases with P/LP variants, findings informed patient prognosis, specialist care, clinical management, and familial recurrence risk. Machine learning approaches were used to segregate the probability of diagnosis for participants based on HPO terms.

**Interpretation:**

WGS is clinically useful for diagnosis and management of genetic conditions associated with CP in the UK. Validation of these findings in a larger cohort is warranted.

**Funding:**

Rosetrees Charitable Trust, Isaac Newton Trust, NIHR Cambridge Biomedical Research Centre, and the Wellcome Trust.

**Research in context**

**Evidence before this study**

Our research built on a comprehensive narrative review we published in 2024 (Basu et al., Developmental Medicine & Child Neurology, 2025), which included a systematic PubMed search of genetic studies in cerebral palsy published between 2017 and 2024. We included studies reporting genetic diagnostic yields in predominantly paediatric cerebral palsy cohorts using whole exome or whole genome sequencing, with or without copy number variant analysis. We excluded case reports, cohorts of fewer than 50 participants, highly selected populations enriched for genetic diagnoses, studies focused on specific genetic syndromes, and those without clear cerebral palsy diagnostic criteria.

Nine relevant studies from Australia, China, Canada, and the USA included approximately 4284 individuals with cerebral palsy. Genetic causes were identified in at least 9% of cases, with most studies using exome sequencing. However, study design, ascertainment, variant interpretation, and reporting thresholds varied substantially. Risk of bias included selective recruitment, variable phenotypic characterisation, and inconsistent variant classification. No prospective studies had been conducted within the UK National Health Service, limiting evidence for this healthcare setting.

**Added value of this study**

This is the first prospective study of genetic diagnosis in cerebral palsy conducted within the NHS using whole genome sequencing in an unselected cohort recruited from specialist paediatric clinics. We identified pathogenic or likely pathogenic variants in 12.8% (11/86) of participants, with a further 9.3% carrying variants of interest, suggesting that approximately one in five children have clinically relevant genetic findings in routine NHS practice. Genetic diagnosis altered clinical management in all diagnosed cases, including modified specialist care (100%), new specialist referrals (64%), and prognostic counselling (82%). We developed and validated machine learning models using Human Phenotype Ontology terms that showed good discrimination (AUC 0.88) for predicting diagnostic yield, identifying intellectual disability, dystonia, and multisystem phenotypes as positive predictors, while spasticity and prematurity were associated with lower diagnostic probability. Performance of the modelling approach was consistent in an independent Genomics England cerebral palsy dataset.

**Implications of all the available evidence**

Available evidence suggests that 9–36% of individuals with cerebral palsy have an identifiable genetic cause. In an unselected UK paediatric cohort, our findings indicate that approximately one in five children have clinically relevant genetic findings, providing a realistic estimate for NHS service planning. These findings support incorporating genetic testing into routine cerebral palsy assessment because of its direct impact on clinical management. Machine learning approaches may further improve patient selection from electronic health records, increasing the efficiency of genomic testing programmes. Future studies should refine predictive models, evaluate cost-effectiveness, and assess the long-term impact of genetic diagnosis on patient and family outcomes.

## Introduction

Cerebral palsy (CP) has been described as “a group of permanent disorders of the development of movement and posture, causing activity limitation, that are attributed to non-progressive disturbances that occurred in the developing foetal or infant brain”.^1^ In addition to motor disorders, CP is often accompanied by disturbances of sensation, perception, cognition, communication, and behaviour, as well as by epilepsy, and secondary musculoskeletal problems.^1^ CP is the most common cause of childhood-onset lifelong physical disability,^2^ affecting over 18 million people globally.^3^ It is estimated that 1 in 400 children born in the UK are affected by CP, equating to approximately 1700 new cases each year.^4^ Recognised risk factors for CP include preterm birth, multiple births, placental pathology, intrauterine infection, intrauterine growth restriction, neonatal stroke, brain malformations, and hypoxic-ischaemic encephalopathy. In most cases, one or more of these factors is present, but for approximately a third of individuals with CP, evidence of such antenatal, perinatal and neonatal sentinel events is lacking.^5^

International studies from Australia, China, Canada, and the USA identified genetic causes of CP in approximately 9–36% of cases.^6^, ^7^, ^8^, ^9^, ^10^, ^11^, ^12^, ^13^, ^14^, ^15^ There is a growing list of genes with evidence for association with CP, movement and neurodevelopmental disorders,^16^ cerebrovascular events or brain structural anomalies.^17^ Importantly, the potential impact of a genetic diagnosis includes eligibility for clinical trials, treatment modification with existing drugs, and prognostication, enabling changes in specialist management based on gene-specific clinical features. For family members, genetic diagnosis can help in clarifying recurrence risk for future pregnancies.^8^ Thus, a genetic diagnosis may have clinical utility for the affected person as well as extended family members.

We report the results of the first prospective UK study designed to assess potential genetic diagnoses in an unselected CP cohort and how diagnosis impacts care in the National Health Service (NHS). We deployed whole genome sequencing (WGS) in five consultant-led paediatric outpatient specialist clinics with diverse patient populations to determine the rate of genetic diagnosis, impact to care following positive molecular diagnoses, and, through clinical and family surveys, the perceived value of WGS in the management of CP. The impact of negative diagnostic findings was not assessed. We also evaluated whether documented clinical features translated into human phenotype ontology (HPO) terms^18^^,^^19^ could improve prediction of a genetic diagnosis. This study provides evidence to inform the implementation of genomic testing for children with CP within the NHS and other publicly funded healthcare systems.

## Methods

### Study design, patient participation, involvement and engagement (PPI/E)

This study was conducted and reported in accordance with the STROBE guidelines (Supplementary Table S1).

Prior to study design, a patient and family engagement exercise was carried out to gauge pre-study interest in participation and acceptability of WGS testing for children with CP through anonymised surveys at three recruiting specialist paediatric clinics (Cambridge, Colchester, Newcastle). The results were collated and informed the design of patient and carer-facing documents used in the study.

Study participants, children aged <17 years with a clinical diagnosis of CP (documented in the medical record), were recruited from April 2023 to November 2024. Wherever possible, children were recruited with one or both biological parents for duo/trio analysis; however, children who were adopted or for whom neither biological parent was available were also recruited. Siblings affected with CP were also recruited if they met eligibility criteria. Children with a pre-existing genetic diagnosis were excluded. Participants were not stratified by phenotype for recruitment purposes.

Participants were identified by clinicians at five NHS England specialist clinics within Bedfordshire Hospitals NHS Foundation Trust, Cambridgeshire Community Services NHS Trust, East Suffolk North Essex NHS Foundation Trust, or Newcastle upon Tyne Hospitals NHS Foundation Trust. Referring clinicians comprised community paediatricians and specialists in paediatric neurology and neurodisability based in community or specialist tertiary care clinics. The ‘English indices of deprivation 2019’^20^ was used to annotate the postcode data on indices of multiple deprivation (IMD) deciles, a relative measure of socioeconomic status in the UK, to ensure representation of underserved communities in this cohort.

### DNA sequencing

Genomic DNA was extracted from blood samples as described.^21^ DNA was prepared using the “Illumina DNA PCR Free Library Prep Kit” following manufacturer's instructions (Illumina, San Diego, CA). Sequencing was performed by Illumina Inc, on the Illumina NovaSeq™ 6000 Sequencing System following the manufacturer's standard protocols (more details in Supplementary Methods). Quality control metrics were evaluated as described in Supplementary Methods.

### Secondary analysis

Variants were called using the DRAGEN™ secondary analysis pipeline version 4.3 (see Supplementary Methods).

### Tertiary analysis

Variant analysis including filtering, prioritisation and interpretation was performed using Emedgene™ software, v35, that uses explainable AI to generate a shortlist of potential causative variants (Supplementary Methods).

In addition to gene agnostic analysis, a comprehensive list of 1503 genes associated with CP compiled from peer-reviewed databases and the literature (the ‘CP gene list’; see Supplementary Table S2) was applied as detailed in Supplementary Methods. Variants were interpreted following the latest variant interpretation guidelines from the American College of Medical Genetics and Genomics (ACMG) and the Association for Molecular Pathology for SNVs,^22^ and the ACMG and Clinical Genome Resource (ClinGen) for CNVs^23^ with modifications suggested by the best practice guidelines for variant classification in rare disease from the Association for Clinical Genomic Science.^24^ Variants were classified as pathogenic (P), likely pathogenic (LP), variants of uncertain significance (VUS) or non-diagnostic, i.e. not meeting criteria for VUS and not relevant to the participant's presentation. Variants that potentially explained some features of the probands' phenotypes- ‘variants of interest’ (VOI) or VUS–were discussed in multidisciplinary team meetings. Prior to reporting, P/LP variants were orthogonally confirmed by the East Genomic Laboratory Hub (East GLH), or for the *ATL1* variant identified in NNET3025, by the Northeast and Yorkshire GLH. All results (including negative reports with no clinically relevant findings) were issued to the referring clinician in the form of a research report. A clinically actionable report was issued for cases with a confirmed P or LP variant. Genetic findings not associated with the primary reason for referral (i.e., incidental or unexpected findings including VOI), and VUS, were not reported. Variants classified as P, LP and VUS, found in genes on the CP gene list were submitted to ClinVar.

VUS were sub-classified as ‘High/Mid/Low’ using posterior probabilities estimated from a Bayesian approach and a point-scale derived from the Bayesian approach as described in Durkie et al.^24^

### Clinical data collection

Clinical/phenotype data were provided by referring clinicians via an online questionnaire (Qualtrics) using International Cerebral Palsy Genomics Consortium (ICPGC)-defined common data elements.^25^ Data were manually converted to human phenotype ontology (HPO) terms. Additional HPO data were obtained by reviewing clinic letters provided by the referring clinician or accessed through electronic health records. All data were pseudonymised. Data linkage was possible through the participant study identifier.

### Data storage

Clinical data and pedigree information were stored in Phenotips (https://www.eastgenomics.nhs.uk/about-us/genomic-medicine-service-alliance/Transformation_Projects/Phenotips/). After tertiary analysis, all processed WGS data were backed up on the ISO 27001 certified secured University of Cambridge Secure Research Computing Platform (SRCP) storage (https://www.hpc.cam.ac.uk/secure-research-computing).

### Phenotype data analysis

HPO terms were analysed using the OntologyX suite of packages^26^ in R (v4.5.0). The corresponding system-level abnormality for each HPO term was computed, to inform the breadth of systems captured by the dataset.

The HPO dataset was reviewed for the presence and absence of ‘red flag features' (adapted from Basu et al., Supplementary Table S3)^27^ using the *‘get_descendants*' and ‘*get_ancestors*’ functions in the OntologyIndex R package. Red flag features were counted according to the presence of the relevant HPO term per individual and evaluated for differences between participant diagnostic categories (explained below).

For development of an HPO-based predictive model based on diagnostic grouping per participant, a non-redundant (or ‘minimal set’) of HPO terms was used. The diagnostic grouping was based on participants with Reported, VOI and High VUS variants in the ‘positive’ group, and non-diagnostic or Low VUS in the ‘negative’ group (n = 72 from NeuralNET CP cohort, termed ‘NNET’). Datasets from the Next Generation Children's (‘NGC’) study (Cambridge South Research Ethics Committee approval 13/EE/0325) for individuals where CP was part of their phenotype (n = 10), and cases with a genetic diagnosis from Fehlings et al.^13^ (‘Fehlings’24′, n = 25) were also incorporated resulting in a total dataset of 107 samples. These additional datasets were included to increase the sample size, in particular, the number of positive genetic diagnoses, to improve model classification performance. We independently trained and validated the model with data from a Genomics England rare diseases CP cohort (‘GELCP')^28^ following the same approach described in Supplementary Methods. Participants were selected if they had at least three HPO terms, with ‘cerebral palsy’ included, and had a ‘resolved’ or ‘unresolved’ diagnostic status (n = 117). The visualisation of the different datasets and GELCP cohort evaluation is described in Supplementary Methods.

### Statistical analysis

All statistical analyses were performed in R (v4.5.0). For evaluating differences between the number of red flag features between positive and negative diagnostic categories, the Kruskal–Wallis test was performed in R, with post-hoc pairwise Wilcoxon rank sum tests (Benjamini-Hochberg adjusted).

### HPO-based, machine learning model development to discriminate between diagnostic categories

To develop a phenotype-based model for estimating the likelihood of a genetic diagnosis, HPO terms associated with the predefined ‘positive (P/LP, VOI, High VUS)’ and ‘negative (Low VUS, Non-diagnostic)’ diagnostic groups were compared. Features such as family history were not included within the model as these were not present within the additional datasets included in model development. To standardise annotations, obsolete terms were updated and duplicate HPO terms removed. The resulting binary matrix of HPO terms was used as model input. Dimensionality reduction was performed using Principal Component Analysis (PCA) to address the high ratio of features-to-cases and reduce multicollinearity. Linear Discriminant Analysis (LDA) was then trained on the principal components to maximise separation between positive and negative groups. Participants were randomly split into training (70%) and testing (30%) datasets. A comparison model was also constructed by combining PCA with a Gradient Boosting Model (GBM).

Model performance was evaluated using sensitivity, specificity, receiver operating characteristic (ROC) curves generated using the ‘pROC’ (v1.18.5) package, and area under the curve (AUC). To maximise clinical utility, the classification threshold was explicitly selected to prioritise clinical sensitivity rather than a standard balanced Youden Index. The contribution of individual HPO terms to model predictions was assessed by iteratively removing each term from patient phenotypes and recalculating classification probabilities. The mean change in predicted probability for each term was calculated across all relevant cases, with 95% confidence intervals generated to quantify its influence. Visualisation of term contributions was performed using volcano plots (ggplot2 v3.5.2 and ggrepel v0.9.6). Beyond traditional discriminative metrics, extended evaluations including global model calibration, decision curve analysis (DCA), and threshold-specific absolute classification counts were performed to validate clinical net benefit and ensure study compatibility with future meta-analyses; detailed descriptions of these advanced statistical workflows and model development are provided in the Supplementary Methods.

### Ethics approval

The NeuralNET for Cerebral Palsy (IRAS 319781; NCT05858268) study protocol was approved by Wales Research Ethics Committee 5 (23/WA/0012) and the Health Research Authority. Written informed consent was obtained from all participants or their parents/guardians.

### Role of the funding source

This study was funded by the Rosetrees Charitable Trust, Isaac Newton Trust, Wellcome Trust and NIHR Cambridge Biomedical Research Centre. No financial resources derived from Illumina; Illumina personnel participated in DNA sequencing, case analysis and variant interpretation as per contract with University of Cambridge. The study was designed by HHP, EL, AJC, TER, DHR; all authors participated in analysis, writing and decision to submit the paper for publication.

## Results

### Recruitment, demographic and clinical characteristics of cohort

**Fig. 1:**
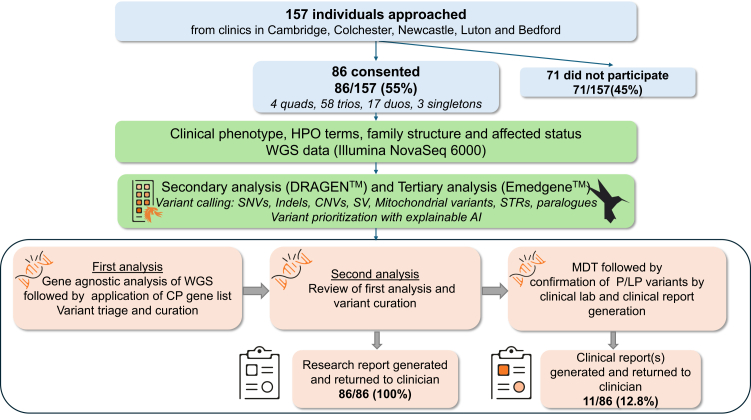
Overview of the study workflow. Blue sections indicate that the overall number of participants and families approached and consented; green sections describe the data collation processes; pink sections discuss the approach to case analysis and variant interpretation, report generation and the overall diagnostic yield.

**Fig. 2:**
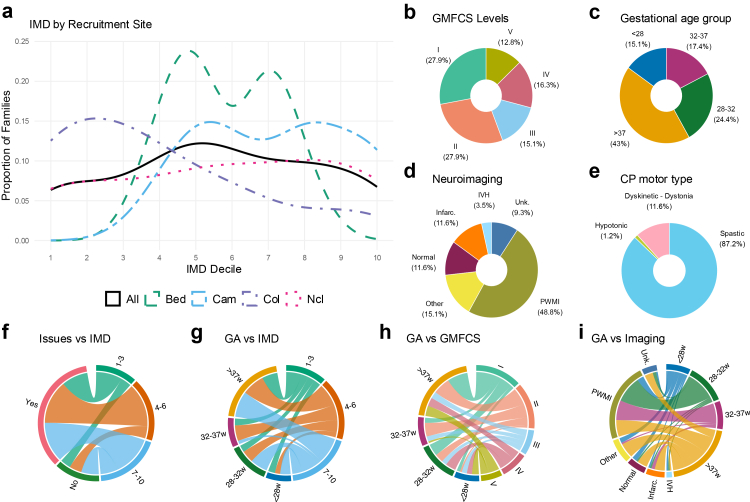
Demographics and clinical characteristics of the cohort. a: Density/proportion of participants per Indices of multiple deprivation (IMD) decile by site of recruitment (Bedford/Luton (‘Bed’), green; Cambridge (’Cam’), blue; Colchester (‘Col’), purple; Newcastle (‘Ncl’), pink). Overall IMD decile proportions indicated by black line. b: Cohort Gross motor function classification system (GMFCS) levels I (mild) to V (severely limited). c: Gestational age, corrected to week. d: Neuroimaging findings: *Infarc.,* Infarction/stroke; *IVH*, intraventricular hemorrhage; *PWMI*, perinatal white matter injury; *Unk.*, unknown. e: Predominant motor type of cerebral palsy (CP) recorded at time of recruitment to study. (f–i): Chord diagrams highlighting relationships (f) between IMD group (1–3, 4–6, 7–10, i.e. most deprived to least deprived) and perinatal issues (premature birth, pregnancy or birth related issues, neonatal infection); (g) IMD group and GA (<28 weeks, extremely premature to >37 weeks, term gestation at birth); (h) GMFCS level (I–V) and GA; and (i) GA versus neuroimaging findings. GA, Gestational age; w: weeks.

Prior to initiating the study, people under age 18 with CP, families, carers and advocacy groups in the UK were surveyed to explore acceptability of genetic testing diagnosis for CP patients. As shown (Supplementary Figure S1), 56% of respondents indicated they would have genetic testing if offered, supporting the study launch. Of 157 individuals prospectively invited to take part in the study, 86 (55%) consented to participate (Fig. 1). The demographic features and clinical characteristics of the cohort are shown in Table 1 and Fig. 2. Recruitment via the NHS reduced ascertainment bias. We used index of multiple deprivation (IMD) for participants by postcode location to confirm representation across all IMD deciles (Fig. 2a), with Bedford and Colchester showing a lower IMD profile compared to Cambridge. Cohort severity indicated by the GMFCS scores, indicated 48 participants (55.8%) had milder CP (GMFCS I-II), with the remainder having GMFCS III-IV (Fig. 2b). Gestational age demographics for the cohort consisted of 43% full term, and 15% were extremely preterm (Fig. 2c). The predominant neuroimaging finding was perinatal white matter injury (PWMI; 49%) followed by infarction (Fig. 2d); the predominant CP motor type was spastic (Fig. 2e). There were no significant correlations between the demographic features in the dataset (Fig. 2f–i). For example, children with perinatal issues were represented across IMD deciles (Fig. 2f), GMFCS V fell into both extremely preterm birth (<28 weeks) and term categories (Fig. 2h), and findings of PWMI were distributed across the cohort (Fig. 2i).

**Table 1:** Demographics and summary clinical characteristics of cohort.

Demographics	Number of patients	86
	Age, median (IQR) [range]	10.5 (6.3–13.4) [2.4–16.5]
	**Sex**	
	Male	55 (64%)
	Female	31 (36%)
	Family structure^a^	58 trios, 4 quads, 17 duos, 3 singletons
Cerebral palsy	**GMFCS**	
	GMFCS I	24 (28%)
	GMFCS II	24 (28%)
	GMFCS III	13 (15%)
	GMFCS IV	14 (16%)
	GMFCS V	11 (13%)
	**Predominant motor type**	
	Dyskinetic—Dystonia	10 (12%)
	Hypotonic	1 (1%)
	Spastic	75 (87%)
Additional clinical features^b^	Neurodevelopmental abnormality	52 (61%)
	Epilepsy	29 (34%)
	Autism	37 (42%)
	Visual impairment	38 (44%)
	Hearing impairment	4 (5%)
	Congenital abnormalities	27 (32%)
Birth information	**Prematurity**^c^	
	Extremely preterm	15 (17%)
	Very preterm	19 (22%)
	Moderate to late preterm	15 (17%)
	Born at term	37 (43%)
	**Birth weight**	
	<10% centile	7 (8%)
	>10% centile	59 (69%)
	Not reported	20 (23%)

Sex, age, family structure, gross motor function classification system (GMFCS) level, cerebral palsy motor type, additional clinical features (e.g. hearing impairment, congenital abnormality etc.), neonatal data (gestational age, birth weight).

a 56 trios included a child with cerebral palsy and both biological parents. Two trios included two affected children and one biological parent.

b Some patients had more than one additional clinical feature

c Extremely preterm is defined as birth before 28 completed weeks of gestation, very preterm is defined as birth before 32 completed weeks of gestation, moderate to late preterm is defined as birth at 32–36 completed weeks of gestation, and born at term is defined as birth at 36+ weeks gestation.

### Genetic findings

**Fig. 3:**
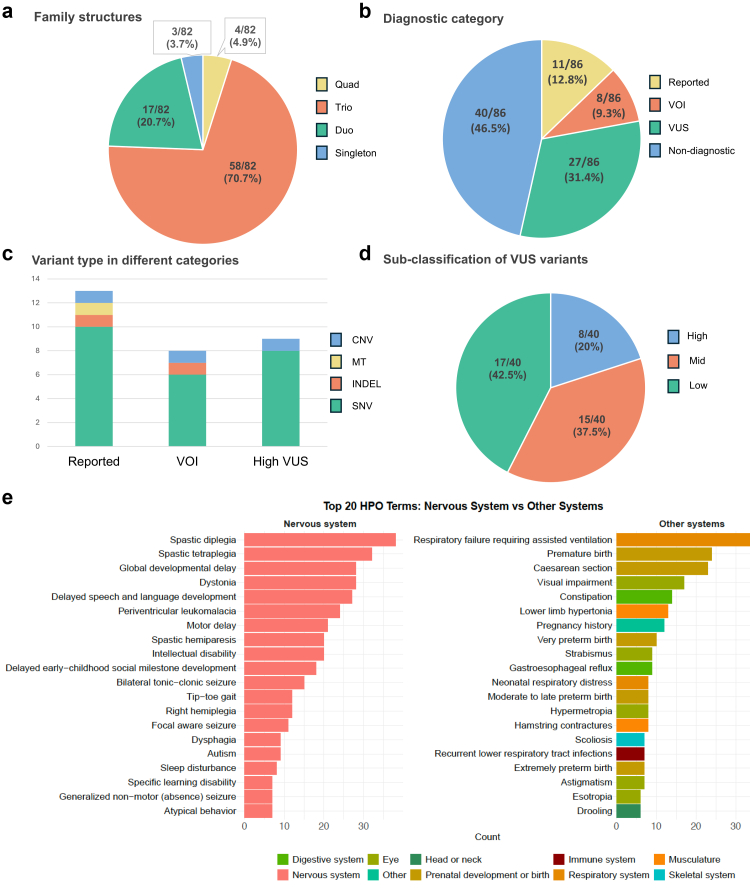
Genetic testing results and phenotype overview. a: Pie chart of family structures included in the NeuralNET cerebral palsy cohort. b: Diagnostic category of cases based on variants detected. Reported: cases containing variants that were confirmed pathogenic or likely pathogenic by the NHS East Genomic Laboratory Hub (EGLH) and returned in a clinical report; VOI (variant of interest) cases containing VOI that were classified mostly as P/LP but did not meet diagnostic criteria of EGLH or were considered an incidental finding (Supplementary Table S5); VUS—cases containing variants classified as VUS in genes from the CP gene list (Supplementary Table S2); Non-diagnostic: cases from the cohort which did not contain P/LP, VOI or VUS variants as defined above. c: Variant type per diagnostic category of case containing ‘Reported’, ‘VOI’ and High VUS variants. d: Sub-classification of 40 VUS variants found across the 86 participants into High, Mid or Low. e. Frequently used human phenotype ontology (HPO) terms across the NNET cohort. The plot on the left shows the top 20 terms associated with the nervous system (in red). The plot on the right shows the top 20 terms associated with other systems, coloured by system as per the legend, highlighting the large proportion of patients who were premature at birth or had perinatal issues such as respiratory failure.

We performed WGS on DNA from 86 participants with a diagnosis of CP from 82 families, including 58 trios (56 with index child and both biological parents and two with two affected children and one biological parent), four quads, 17 duos and three proband only cases (Fig. 3a). Following WGS, cases were divided into four diagnostic groups: Reported, VOI, VUS or Non-diagnostic (Fig. 3b). There were 12 reported P/LP variants from 11/86 (12.8%) cases (Table 2, Supplementary Table S4). Eleven of the variants were prioritised by the AI algorithm in Emedgene™ as “Most Likely”. One of the variants, *F11* c.901C > T p. (Phe301Leu), was given a lower prioritisation by the AI as a candidate variant and was found by review of all known pathogenic variants in the CP gene list. Notably, five variants that were reported in 4/11 diagnosed individuals were not in the CP gene list. Table 2 lists the reported variants and the impact to the patient as returned by the referring clinician. 8/86 cases (9.3%) had genetic findings we considered to be VOI (Table 3, Supplementary Table S5). VUS in CP genes were found in an additional 27 individuals (Supplementary Table S6), and 40 cases had no relevant genetic findings. Two cases, NNET2019 and NNET3023, contained both a reported variant and a VUS, whilst one case, NNET2010, contained a VOI and a VUS (Supplementary Tables S4–S6). A range of variant types were identified across the diagnostic categories including single nucleotide variants (SNVs), indels, copy number variants (CNVs) and one mitochondrial variant (Fig. 3c).

**Table 2:** Pathogenic and likely pathogenic variants returned in a clinical report.

Impact to the patient as reported by the clinician. Variant classification: P, Pathogenic; LP, Likely pathogenic.

**Table 3:** Variants of interest identified in the NeuralNET cerebral palsy cohort.

Sample ID	Gene	Variant	Variant zygosity	Variant classification	Disorder
NNET1006	*ADAR*	c.577C > G; p. (Pro193Ala)	Heterozygous	LP	Aicardi-Goutières syndrome
NNET1010	*VWF*	c.2561G > A; p. (Arg854Gln)	Heterozygous	P	von Willebrand disease type 2N
NNET2010	*NIPA1;NIPA2*	chr15:22648948–23124083del	Heterozygous	P	15q11.2 microdeletion
NNET2015	*F2*	c.∗97G > A	Heterozygous	P	Cerebral infarction, susceptibility to
NNET3004	*POLG*	c.2209G > C; p. (Gly737Arg)	Heterozygous	P	Primary mitochondrial disease
NNET3024	*PAX8*	c.936dup; p. (Glu313ArgfsTer6)	Heterozygous	LP	Hypothyroidism, congenital, due to thyroid dysgenesis or hypoplasia
NNET4003	*ARFGEF1*	c.1441A > T; p. (Ile481Phe)	Heterozygous	VUS	Developmental delay, impaired speech, and behavioural abnormalities, with or without seizures
NNET4006	*COL11A1*	c.1245+1G > A	Heterozygous	LP	Stickler syndrome

Variant classification: P, Pathogenic; LP, Likely pathogenic; VUS, Variant of uncertain significance.

### Sub-classification of VUS variants

Reporting VUS can be challenging and lead to misunderstanding of the significance of the variant. It is possible to sub-classify VUS based on the likelihood of a variant being pathogenic.^22^^,^^24^ VUS SNVs identified in genes on the CP gene list were sub-classified into High, Medium and Low (Fig. 3d, Supplementary Methods and Supplementary Table S6). Eight variants (20%) had multiple pieces of evidence for pathogenicity and required minimal additional evidence to be reclassified as LP (Fig. 3d). For example, an intronic variant in *COL4A1*, c.652-14T > G, was identified in one individual and classified as High VUS based on its rarity in population databases, strong *in silico* support from Splice AI for an effect on splicing and phenotypic features matching those of other cases with variants in this gene. RNA studies on skin fibroblasts demonstrating a definite effect on splicing could provide the additional evidence needed to reclassify this variant as LP. Overall, four individuals were found to have heterozygous collagen-related gene variants classified as VUS (Supplementary Table S6). One individual with a LP variant *COL11A1*, c.1245+1G > A, did not show sufficient phenotype correlation, therefore was categorised as VOI (Supplementary Tables S5 and S7). The five individuals with collagen-related gene variants were all born prematurely and had neuroimaging abnormalities, predominantly PWMI, which could not be attributed to the gene variants.

### Genetic diagnosis resulted in impacts to care in all solved cases

Genetic testing had not previously been offered to 9/11 individuals whom we determined to have diagnostic variants. The diagnostic findings identified in this study were associated with a broad range of rare genetic disorders and comorbidities, which previously were thought to be part of the CP presentation, but were explained by the genetic aetiology (Supplementary Table S8). In each case the diagnosis impacted care according to the referring clinician's feedback report (Table 2). In all 11 cases, the genetic diagnoses provided recurrence risk information that may be relevant to the patient's family now or in the future. In 4/11 individuals, diagnosis enabled tailored clinical management. For example, prospective screening and physiotherapy plans were modified due to finding of a pathogenic variant in *PMP22* associated with Charcot-Marie-Tooth disease. Referral to a specialist mitochondrial service was initiated due to a pathogenic *MT-ATP6* variant associated with primary mitochondrial disease, and a medical alert regarding general anaesthesia risk was arranged. Identification of a LP variant in *SCN1A* (associated with Dravet syndrome) resulted in a change in antiseizure medication. Referral to a paediatric cardiology clinic was initiated due to a pathogenic variant in *COL4A1* to screen for *COL4A1*-associated heart conditions. In 9/11 (82%) cases, the referring clinician reported that the genetic diagnosis provided important prognostic information for the patient, in 10/11 (91%) cases the diagnosis informed existing specialist care, and in 7/11 (64%) cases the diagnosis informed new specialist care. Moreover, further advantages could be accrued because of genetic diagnosis due to eligibility for future clinical trials or management in national centres of excellence. Additionally, informal feedback indicated that negative results were also appreciated by family members as it helped focus their efforts on rehabilitation therapies and clarified that the condition was not inherited. Together these findings highlight the broad range of clinical impact that WGS for CP can have on children and their families in the UK (Table 2).

### Predominant phenotypes in cohort

We collected 367 non-redundant HPO terms translated from clinical features submitted by clinicians in an online data collection tool (Qualtrics) and additional data manually extracted from electronic health records. Of these, the 40 most common HPO terms are shown in Fig. 3e. Predominant terms were neurological, related to the CP spastic motor type, followed by terms denoting neurodevelopmental delay, e.g., motor or speech/language delay, global developmental delay, and intellectual disability. Seizures and dystonia were frequent phenotypes in this cohort. Microcephaly was present in one case. Respiratory, digestive, eye, and immune system HPOs were observed with frequent visual impairment, constipation and chest infection terms. Less frequently documented clinical features in our study cohort were endocrine and metabolic disturbances (Supplementary Figure S2).

### Approaches to estimate likelihood of genetic diagnosis from phenotypic features

**Fig. 4:**
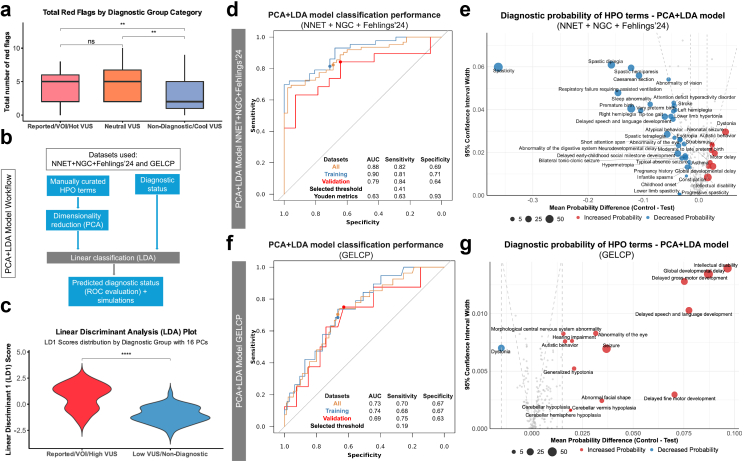
Visualisation of HPO data mapped to diagnostic groups, and development of a model to discriminate diagnostic categories by HPO data. a: Total red flag features shown per participant per diagnostic category (NNET cohort only). b: Simplified workflow for modelling strategy and evaluation. Diagnostic status includes ‘Positive’ for P/LP/High VUS across NNET + NGC + Fehlings'24 or ‘solved’ in GELCP, and ‘Negative’ includes Non-diagnostic/Low VUS results across NNET + NGC + Fehlings'24 or ‘unsolved’ in GELCP. See Supplementary Figures S5 and S6 for methodology. c: Violin plots to display differences between the diagnostic categories for NNET + NGC + Fehlings'24 dataset using LDA evaluation of HPO terms. d: ROC curves for NNET + NGC + Fehlings'24 dataset (n = 107); Training (n = 75), Validation (n = 32). e: Volcano plot to highlight HPO terms with increased or decreased probability of a genetic diagnosis in the context of a child with cerebral palsy. HPO terms are labelled if the absolute mean probability difference is greater than 0.015 and exceeds the width of its 95% confidence interval. The size of each data point is proportional to the HPO term's frequency in the cohorts. f: ROC curves for GELCP dataset (n = 117); Training (n = 82), Validation (n = 35). g: Volcano plot as per E, for the GELCP dataset. AUC: Area under the curve; Fehlings'24: Fehlings et al. (2024) cohort; GELCP, Genomics England rare disease Cerebral Palsy dataset; HPO, Human Phenotype Ontology; LDA, Linear Discriminant Analysis; NGC, Next Generation Children's project cohort; NNET, NeuralNET Cerebral Palsy cohort; PCA, Principal Component Analysis; ROC, Receiver Operating Characteristic; VOI, Variant of interest; VUS, Variant of uncertain significance.

Clinical consensus is lacking regarding which patients with CP should be offered WGS. Development of clinical decision tools could help in deciding the probability of a genetic condition associated with CP diagnosis. We proposed that CP presentation owing to a genetic condition might be recognised from so called ‘red flag’ clinical features (defined as phenotypic features in 25 publications which reported CP genetic diagnoses; Supplementary Table S3).^27^ To test this, we first conducted HPO-based analyses and red flag features associated with clinical presentation, perinatal history, additional investigations, targeted investigations, examination, and family history (Supplementary Figure S3). While we found no statistically significant differences between the diagnostic groups in terms of total HPO terms, we observed a significant (*p =* 0.01, Kruskal–Wallis test) difference in average number of red flag features (Fig. 4a), with a median of 2 red flag features in the ‘Non-diagnostic/Low VUS’ category compared to a median of 5 red flag features in the other categories. This difference was confirmed by post-hoc pairwise Wilcoxon rank sum tests (Benjamini-Hochberg adjusted, *p* < 0.01). Whilst the largest proportion of reported diagnoses were in children born at term and belonged to higher IMD groups (Supplementary Figure S4), there was no obvious pattern to the red flag features with regards to the diagnostic categories (Supplementary Figure S3).

Given these findings, we next developed and trained machine learning models for discrimination of HPO terms associated with an increased likelihood of a genetic diagnosis, comparing aggregated data from the NNET cohort (this study), NGC cohort,^29^ and Fehlings'24 cohort.^13^ We divided subjects into categories of ‘Positive’ (Reported/VOI/High VUS) or ‘Negative’ (Non-diagnostic/Low VUS), excluding the ‘Mid VUS’ group. We used a Principal Component Analysis (PCA)-based approach to identify HPO terms that segregated with classification of the 107 participants in the two groups (Fig. 4b, Supplementary Figures S5, S6). We compared Linear Discriminant Analysis (LDA) and Gradient Boosting Model (GBM) approaches in terms of their ability to discriminate between the two groups with HPO data. As shown (Fig. 4c, Supplementary Figure S7), PCA + LDA evaluation of HPO terms captured significant differences between the two diagnostic groups, this was also seen with PCA + GBM models (Supplementary Figure S8). The PCA + LDA achieved good discrimination between the Positive and Negative diagnostic groups indicated by an area under the curve (AUC) of 0.88 in the overall dataset (Fig. 4d).

To maximise clinical utility and prioritise diagnostic sensitivity, an operational classification threshold of 0.41 was explicitly selected to minimise missed cases. The PCA + LDA model performance was evaluated on data completely held back from model development, using a 100-iteration Monte Carlo cross-validation framework and demonstrated minimal optimism bias. At this selected threshold, the model achieved a sensitivity of 0.84 and a specificity of 0.64 (shown as the Youden Index in Supplementary Figure S7B). Further evaluation confirmed the model's reliability in practice. When predicted probabilities were compared against actual diagnostic outcomes across patient risk groups, predictions closely matched observed results, with no systematic tendency to over- or under-predict risk. Formal calibration statistics supported this finding (Observed-to-Expected ratio: 1.04; Brier score: 0.18; Supplementary Figure S7C). Decision Curve Analysis (DCA) further confirmed its clinical value, demonstrating that utilising the classifier provides a superior population Net Benefit compared to default “test all” and “test none” strategies at threshold probabilities above 0.41. Furthermore, a global Kruskal–Wallis sensitivity analysis mathematically confirmed that the model's performance was highly sensitive to architectural parameterisation. The test across the validation performance matrix demonstrated that AUC scores varied substantially across configurations (H = 968.25, *p* < 0.0001), with a large practical effect size (ϵ2 = 0.142; 95% bootstrap CI: 0.135–0.166). This confirmed that the choice of dimensional restriction impacted predictive optimisation, justifying the necessity of our empirical cross-validation pipeline. Threshold-specific absolute classification counts, complete risk calibration curves, decision curve net benefits, and the parameterisation effect's empirical AUC tuning trajectory are detailed across panels A–D in Supplementary Figure S7.

Although the PCA + GBM model achieved classification performance comparable to PCA + LDA in the validation datasets (Supplementary Figure S8), it entailed a substantially more complex architecture. Due to the reduced complexity of the LDA model, we gained explainable insights about the HPO terms that confer increased and decreased probability towards achieving a genetic diagnosis in the overall dataset. Our findings indicated that the categories of HPO terms associated with increased probability of a genetic diagnosis comprised ‘intellectual disability’, ‘dystonia’, neurodevelopmental disorder terms, and additional multisystem terms ‘constipation’, and seizure phenotypes. Conversely, terms related to spasticity, premature birth and respiratory difficulties associated with decreased probability of a genetic diagnosis (Fig. 4e) in this cohort of unselected participants.

The model was next applied to an independent GELCP cohort dataset (n = 117). This showed transferability/validation of the approach (Fig. 4f), with a clear signal for the terms ‘intellectual disability’ and ‘global developmental delay’ increasing the probability of a genetic diagnosis (Fig. 4g). The more balanced number of solved and unsolved diagnoses in the larger GELCP cohort demonstrated similar training and validation model performance suggesting better prediction of diagnostic category for new cases. Differences in terms conferring reduced probability of a genetic diagnosis (i.e. lack of ‘prematurity’-based terms) were likely due to the different recruitment criteria into the Genomics England rare diseases study. This was evident in the clustering analysis and distribution of the hierarchical levels of HPO terms used across the cohorts which showed that the GELCP cohort appeared into distinct clusters (Supplementary Figure S9).

## Discussion

This is the first study of WGS in an unselected cohort of children with CP in the UK, helping establish genetic variants that are distinct to/or found across national populations. Beyond this, a dimension of the study that adds to our understanding of genotype–phenotype relationships is the development of tools for ascertainment of genetic risk based on HPO terms. We compared approaches using HPO-based ‘red flags’^27^ and developed a supervised machine learning approach to address the gap in knowledge about who should be offered WGS.

Advances in exome and genome sequencing have expanded opportunities for precision medicine, enabling improved prognostic counselling and tailored specialist care.^8^ Recent studies indicate that 9–36% of individuals diagnosed with CP have an underlying monogenic condition.^9^^,^^11^^,^^16^ Despite this, WGS is not currently listed as a specific indication for CP in the National Health Service England National Genomic Test Directory (https://www.england.nhs.uk/publication/national-genomic-test-directories/). This study aimed to assess the diagnostic yield of WGS in children and young people attending specialist paediatric clinics in Bedford, Cambridge, Colchester, Luton, and Newcastle. Pathogenic or likely pathogenic variants were identified in 12.8% of participants, with a further 9.3% carrying a VOI. Together, these findings suggest that approximately 1 in 5 children in this representative unselected clinical cohort had a potentially relevant genetic finding. Importantly, diagnoses either explained the CP presentation or comorbidities which were previously thought to be associated with CP, and mimics of CP were also identified. For several participants the molecular diagnosis appeared to explain part—but not necessarily all—of the phenotype (e.g., epilepsy, neurodevelopmental impairment, additional neurological features), while the motor phenotype remained consistent with CP and often occurred in the context of prematurity and/or neuroimaging abnormalities. We therefore highlight the possibility of dual aetiology in some individuals and emphasise the importance of avoiding diagnostic overshadowing, whereby either the CP label or a genetic diagnosis is assumed to explain all clinical features. Nevertheless, each confirmed diagnosis was associated with changes to patient care or counselling. Both gene-agnostic and targeted CP gene list analyses contributed to these diagnoses, highlighting the potential value of combining these approaches in future studies.

Our diagnostic yield (12.8%) was lower than many international studies, but similar to a recent study from Canada^13^; by including VOIs, we found increased overall yield of 22.1%. Differences in diagnostic rates across studies may result from factors such as population genetics, variant frequency, reporting thresholds, and ascertainment bias. For example, criteria for assigning a diagnosis of CP, recruitment from general practice versus specialist paediatric clinics, and selection for or against individuals with syndromic features might influence patient inclusion to genetic studies. Additionally, insurance status in a private healthcare system (e.g., USA) versus the NHS would introduce ascertainment bias; in this respect it is interesting to note a trend toward diagnosis and higher IMD in this study. Both meta-analyses across countries and further research within UK cohorts (e.g., the UK Biobank) will help establish prior risk scores for genetic testing in CP.

While some monogenic conditions directly explain neurological or neuromuscular disorders, others may impact resilience to birth-related events such as hypoxic-ischaemic encephalopathy or neonatal stroke. Notably, we identified variants in several genes related to vascular integrity—*F2, VWF, COL4A1*—in the reported and VOI groups. Additional variants in collagen-related genes, classified as VUS or VOI, were identified in participants born preterm with positive neuroimaging findings. These associations, documented in several prior studies,^17^^,^^30^ warrant further investigation in larger cohorts of individuals with CP or neonatal stroke with WGS. Finally, we note that in many studies similar to ours, genes identified in people with CP may point to an entirely different diagnosis. Examples include *PLP1*, *SCN1A*, and *TREX1*, genes identified across multiple CP cohorts^31^; their recurrence suggests that CP features may represent part of the broader phenotypic spectrum associated with these genes, rather than coincidental findings.

A 2024 workshop with UK specialist neurologists indicated a range of opinions both on the utility of WGS diagnostics and whether all patients with CP should be generally screened or selected based on clinical judgement. Suggestions for clinical phenotype-driven approaches included number of HPO terms or specific clinical features to guide genetic testing decisions. While admittedly, ours is a small cohort, we exploited its rich HPO annotation to assess these questions. We found no association between the number of HPO terms and the likelihood of a genetic diagnosis, VOI, or VUS; using pre-defined ‘red flag’ features we found non-diagnostic cases tended to have fewer red flag HPOs, but neither pointed reliably to genetic prior risk. In contrast, machine learning PCA-LDA and PCA-GBM models achieved good discrimination between positive and negative diagnostic groups. The simpler PCA-LDA model highlighted that intellectual disability, dystonia, and multisystem phenotypes were associated with a higher probability of genetic diagnosis, whereas spasticity and premature birth conferred lower probability. Further work is however required to ascertain clustering of HPO terms that confer increased probability of genetic diagnosis, because stand-alone phenotypes such as ‘dystonia’ are common in individuals with CP and do not distinguish genetic types.

We acknowledge as a limitation that HPO terms were captured at the time of recruitment to the study, and patients may have developed additional phenotypes since then. This temporal aspect could influence the completeness of phenotypic representation; however, we conducted reverse phenotyping and explored available health records to mitigate this. Furthermore, within the context of childhood-onset conditions, the duration of symptoms is not typically a factor in diagnostic variant evaluation, indicating that age does not introduce significant confounding risks in these multivariable phenotypic models. Interestingly, the model performance was similar when trained and validated exclusively on the GELCP cohort. This highlighted that the modelling approach was reproducible in a different data context, with some shared terms between the datasets seen to confer increased likelihood of a genetic diagnosis. However, a limitation of the datasets used was the difference in inclusion criteria leading to differences in the granularity of phenotypic data gathered. Additionally, a major limitation of this study is the lack of independent external validation using an identically recruited prospective cohort. While we maximised sample size and phenotypic diversity during model training by pooling data from multiple clinical cohorts (NNET, NGC, and Fehlings'24) and demonstrated model transportability using the independent GELCP cohort, a true external prospective replication remains absent as there are no similar prospective studies ongoing. To mitigate this sample constraint and maximise internal validity, we bypassed traditional static splits in favour of evaluating independent out-of-sample subsets across 100 resampling iterations. This rigorous cross-validation framework effectively safeguarded the pipeline against optimism bias and ensured generalisability to unseen patient profiles within these clinical feature matrices. Therefore, our findings suggest machine learning may be useful in clinical decision support tools about when to offer WGS, and that validation of these tools in larger cohorts with similar recruitment strategies is warranted.

### Conclusions

Our findings support use of trio gene-agnostic and panel-based WGS as a clinically appropriate test for some individuals with CP, given the impact on patient care and counselling. Machine learning approaches leveraging detailed phenotypic annotation may further refine patient selection for genomic testing. Future research in larger, deeply-phenotyped cohorts will be important to evaluate cost/benefits of WGS for individuals with CP in national testing frameworks.

## Contributors

HHP, EL, AJC, TER, and DHR conceptualised the study. TER, HHP, AJC, EL, RPM, and JA curated the data. AJC, JMLD, DSS, EL, HHP, and TER performed the formal analyses. ER and DHR acquired funding. MB, AJC, ID, KD, JMLD, ZK, HHP, EL, TN, and TER conducted the investigation. AJC, TER, JMLD, and ASR developed the methodology. EL, HHP, and DHR administered the project. JMLD, IRLK, RPM, and ASR developed the software. DRB, ID, KD, SH, ASR, and DHR supervised the study. AJC, JMLD, IRLK, HHP, and TER were responsible for data visualisation. TER, HHP, AJC, JMLD, IRLK, EL, RPM, and DHR wrote the original draft of the manuscript. TER, HHP, AJC, RPM, JMLD, EL, APB, YdA, AS, BPM, JA, MI, CK, AYT, AHK, DSS, ZK, TN, MB, ASR, TL, HH, ER, DRB, IRLK, ID, KD, SB, SH, and DHR reviewed and edited the manuscript. All authors approved the final version of the manuscript.

## Data sharing statement

De-identified WGS data, variant-level genetic and phenotypic data for this study from participants who have consented to share the data are available upon request from the corresponding author. To facilitate reproducibility, the complete R codebase used for dimensionality reduction, machine learning model training, and statistical evaluation has been made publicly available on University of Cambridge data repository at the following link: https://doi.org/10.17863/CAM.130807. Due to privacy and ethical considerations, the raw data are not publicly available. Data from the National Genomic Research Library (NGRL) used in this research are available within the secure Genomics England Research Environment. Access to NGRL data is restricted to adhere to consent requirements and protect participant privacy. Data used in this research include datasets accessed through RR945. Access to NGRL data is provided to approved researchers who are members of the Genomics England Research Network, subject to institutional access agreements and research project approval under participant-led governance. For more information on data access, visit: https://www.genomicsengland.co.uk/research.

## Declaration of interests

MB, AJC, SH, TN, ASR are employees and shareholders of Illumina, Inc. ZK and DRB are shareholders of Illumina, Inc. TR was supported by the Elizabeth Blackwell Institute AI in Health Award (University of Bristol). BM is a clinical advisor to Kingdom Therapeutics. AB has been recently supported by grants from Legal and General Health Equity fund, BrAInwaves Project, RS Macdonald Charitable Trust, NHS Charities Together Health Innovation Fund, Newcastle Hospitals Charity, Newcastle University Policy Support Fund, NIHR ARC NENC, Great North Children's Hospital Foundation. AB has been an associate editor for Mac Keith Press Editorial Board and British Paediatric Neurology Association Research board member (completed January 2025). ER was paid for me consultancy on gene therapy till January 2023 by SwanBio Therapeutics Ltd. HH and TL are funded by the MRC, Wellcome Trust, UCL/UCLH BRC, UK DRI, Rosetrees Trust, and CPA Research Foundation. DR was supported by funding through Rosetrees Trust, Isaac Newton Trust, NIHR Cambridge BRC and collaborated with Illumina, Inc. staff on design of DNA sequencing protocols.
