# Supplementary material for: Whole genome sequencing in cerebral palsy: a UK paediatric pilot study
Source: Lancet Reg Health Eur. 2026 Jul 30;68:101786. doi: 10.1016/j.lanepe.2026.101786 (PMC13452173; doi:10.1016/j.lanepe.2026.101786)
Supplement: Supplementary file 1 [file mmc1.pdf]

Supplemental Appendix

Table of contents

Supplemental Figures

Figure S1	Patient participation, involvement and engagement.
Figure S2	System level abnormalities across dataset using Human Phenotype Ontology terms.
Figure S3	Heatmap visualization of red flag features identified across the cohort.
Figure S4	Linked plot to visualise associations within the cohort between diagnostic category, gestational age and indices of multiple deprivation decile (IMD) groups.
Figure S5	Flowchart of the PCA + LDA (or GBM) model implementation and selection workflow.
Figure S6	Workflow for HPO Term Impact Analysis.
Figure S7	Results for the PCA+LDA models (NNET+NGC+Fehlings'24 cohorts)
Figure S8	Results for the PCA+GBM models (NNET+NGC+Fehlings'24 cohorts)
Figure S9	HPO hierarchical levels histogram and clustering plot (NNET+NGC+Fehlings'24+GELCP)

Supplemental Tables

Table S1	The Strengthening the Reporting of Observational Studies in Epidemiology (STROBE) Statement
Table S2	CP gene list
Table S3	Red flag features
Table S4	Clinically reported pathogenic or likely pathogenic variants identified in the cohort
Table S5	Variants of interest (VOI) identified in the cohort
Table S6	Variants of uncertain significance identified in the cohort in CP genes
Table S6	Phenotypic details reported in individuals with a P/LP/VOI variant
Table S7	Clinical correlation of pathogenic / likely pathogenic variants identified in the cohort

Supplemental Methods

Supplemental References

Supplementary Figures

Supplementary Figure 1: Patient participation, involvement and engagement.

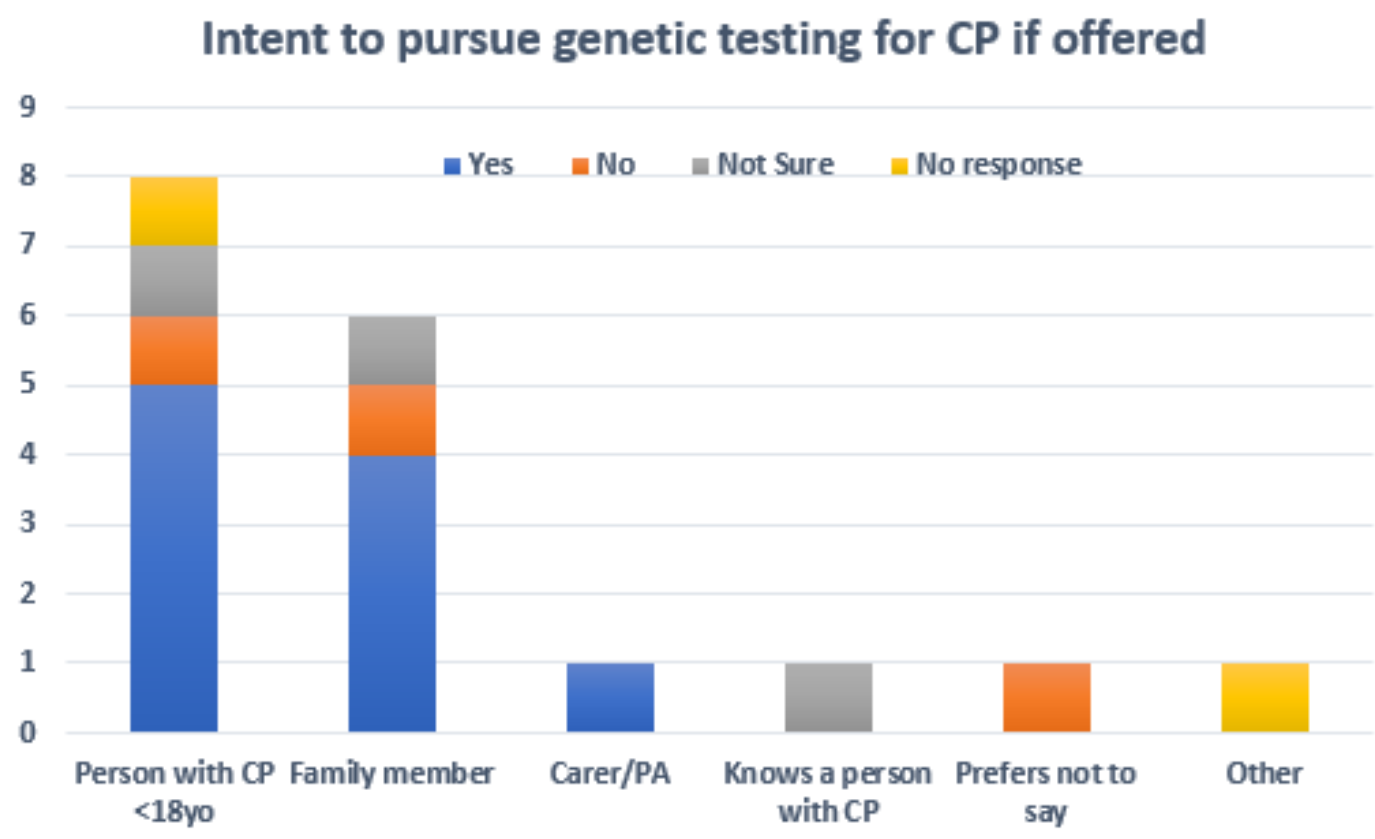

A pre-study survey of attitudes toward genetic testing for CP was administered via CP clinics in the UK to individuals under age 18 with CP, their family members, carers and friends. Of 18 respondents, 10 (56%) said they would pursue genetic testing if it were offered for CP.

Supplementary Figure 2: System level abnormalities across dataset using Human Phenotype Ontology terms.

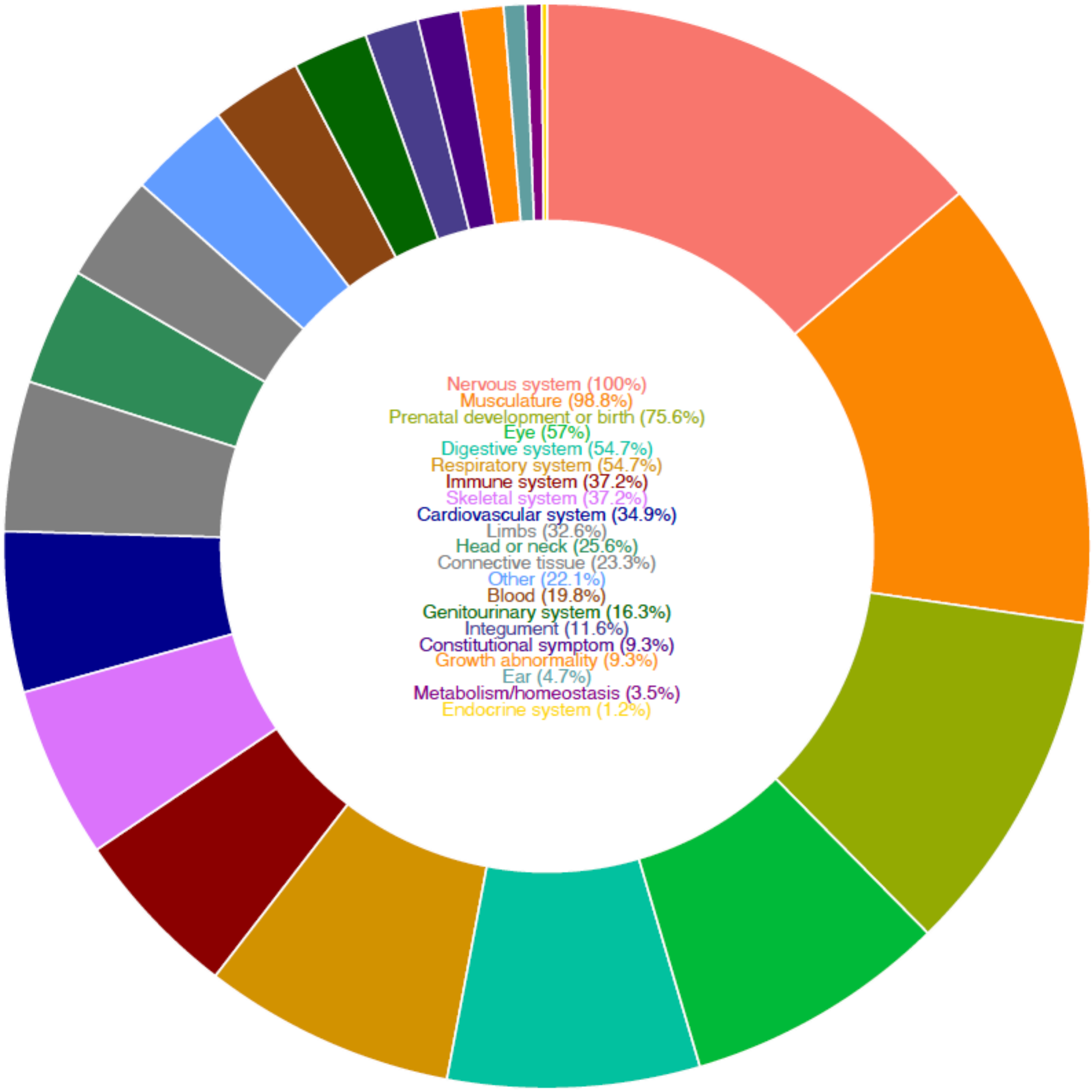

Donut plot to show system level abnormalities across dataset using Human Phenotype Ontology terms. The percentage of cases with terms in the different systems is listed in the centre of the donut.

Supplementary Figure 3: Heatmap visualization of red flag features across cohort.

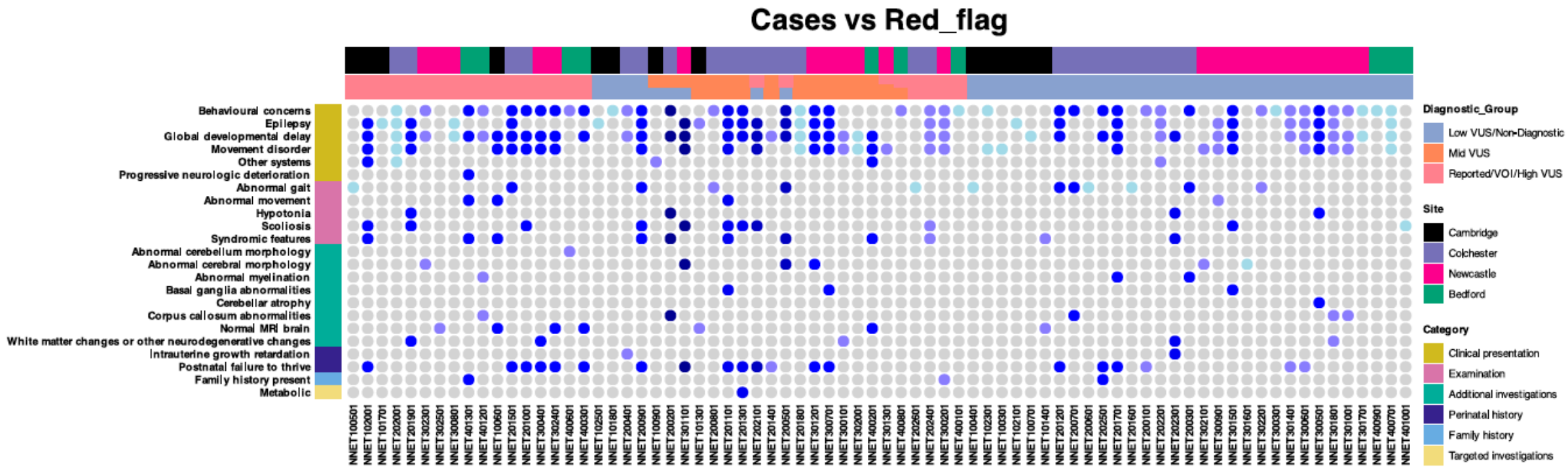

Red flag features are defined as frequent phenotypic features documented across 25 publications which reported genetic diagnoses in children with a prior diagnosis of cerebral palsy adapted from a recent publication Basu et al. 2024) (Supplementary Table 6). Columns represent individual cases, and rows depict red flag features. The colour intensity (light to dark blue) corresponds to the number of HPO terms within each red flag feature per case. Annotations indicate the red flag feature category (according to Basu et al. 2024), the diagnostic group for each case (with stacked bar plots showing the respective proportions for patients with multiple variants), and the recruitment site.

Supplementary Figure 4: Linked plot to visualise associations within the cohort between diagnostic category, gestational age and indices of multiple deprivation decile (IMD) groups.

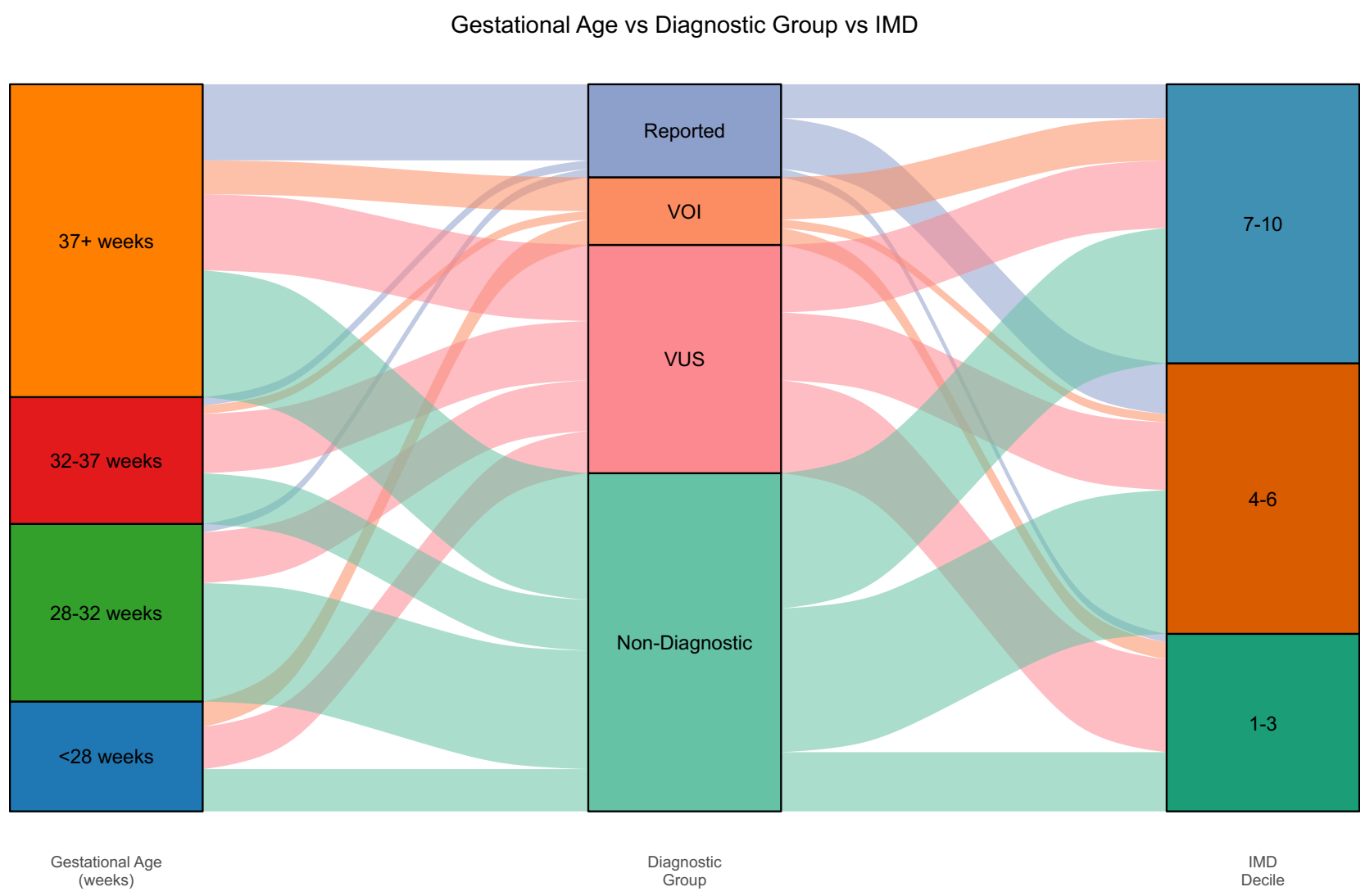

Supplementary Figure 5: Flowchart of the PCA + LDA (or GBM) model implementation and selection workflow.

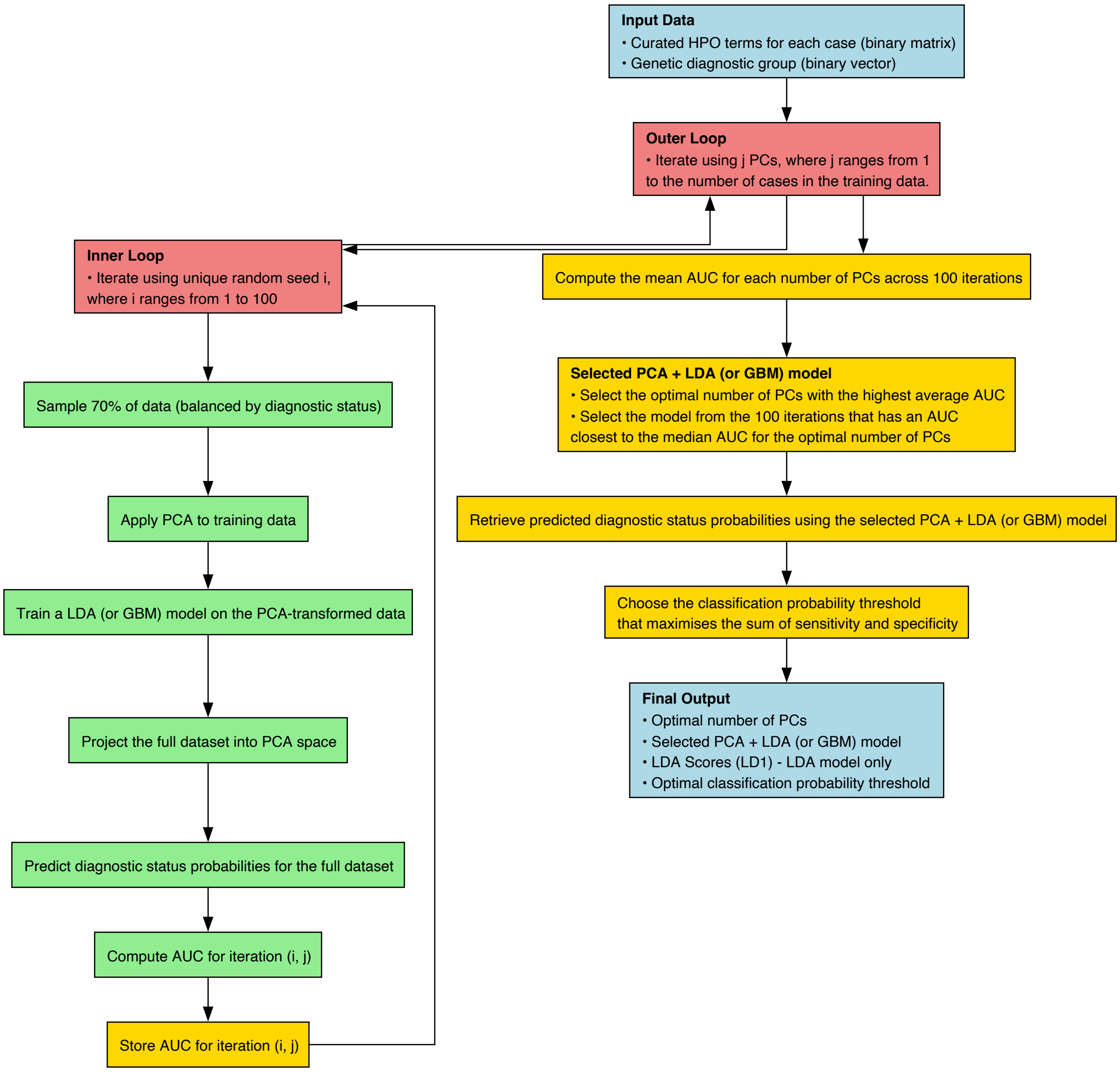

This diagram illustrates the process of predicting diagnostic status from HPO terms, using PCA for dimensionality reduction and LDA (or GBM) for classification. The methodology employs a nested loop structure to determine the optimal number of principal components and select a final, robust model. Each step is visually distinguished by colour: blue boxes represent the initial input data and final outputs; red boxes indicate the outer loop (iterating through the number of PCs) and the inner loop (100 bootstrapped iterations); green boxes show the core computational tasks; and yellow boxes mark the supporting tasks for result aggregation and model selection.

Supplementary Figure 6: Workflow for HPO Term Impact Analysis.

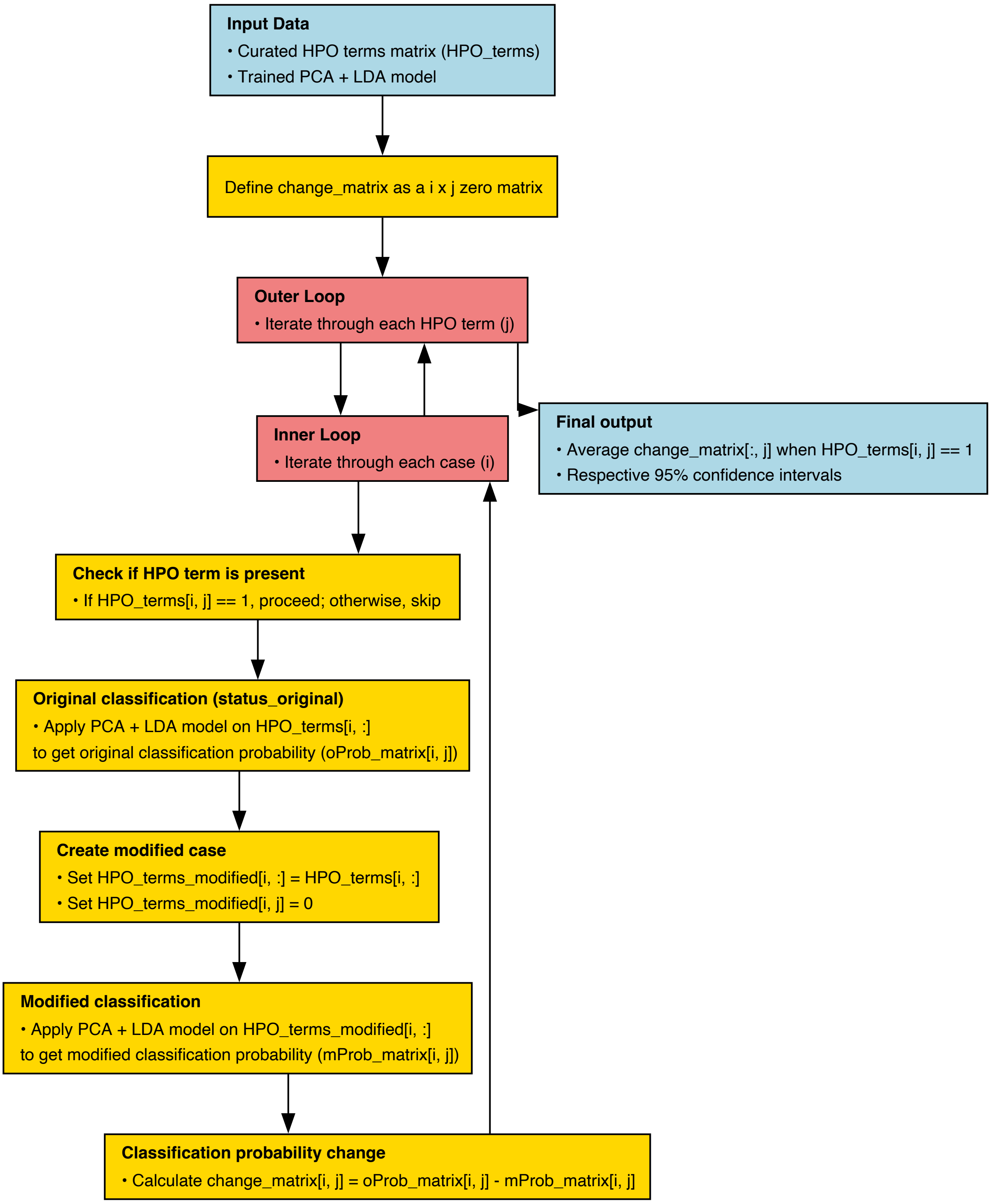

This flowchart details the process of quantifying the impact of removing each HPO term on the classification probability generated by the selected PCA + LDA model. The methodology uses a nested loop to iterate through each term and each case. For cases where a term is present, the change in classification probability upon its removal is calculated. The final output is the average of these changes for each term, providing a measure of its

overall diagnostic importance. Each step is visually distinguished by colour: blue boxes represent input and output, red boxes indicate loops, and yellow boxes show the core computations and supporting tasks.

Supplementary Figure 7: Results for the PCA+LDA models (NNET+NGC+Fehlings'24 cohorts)

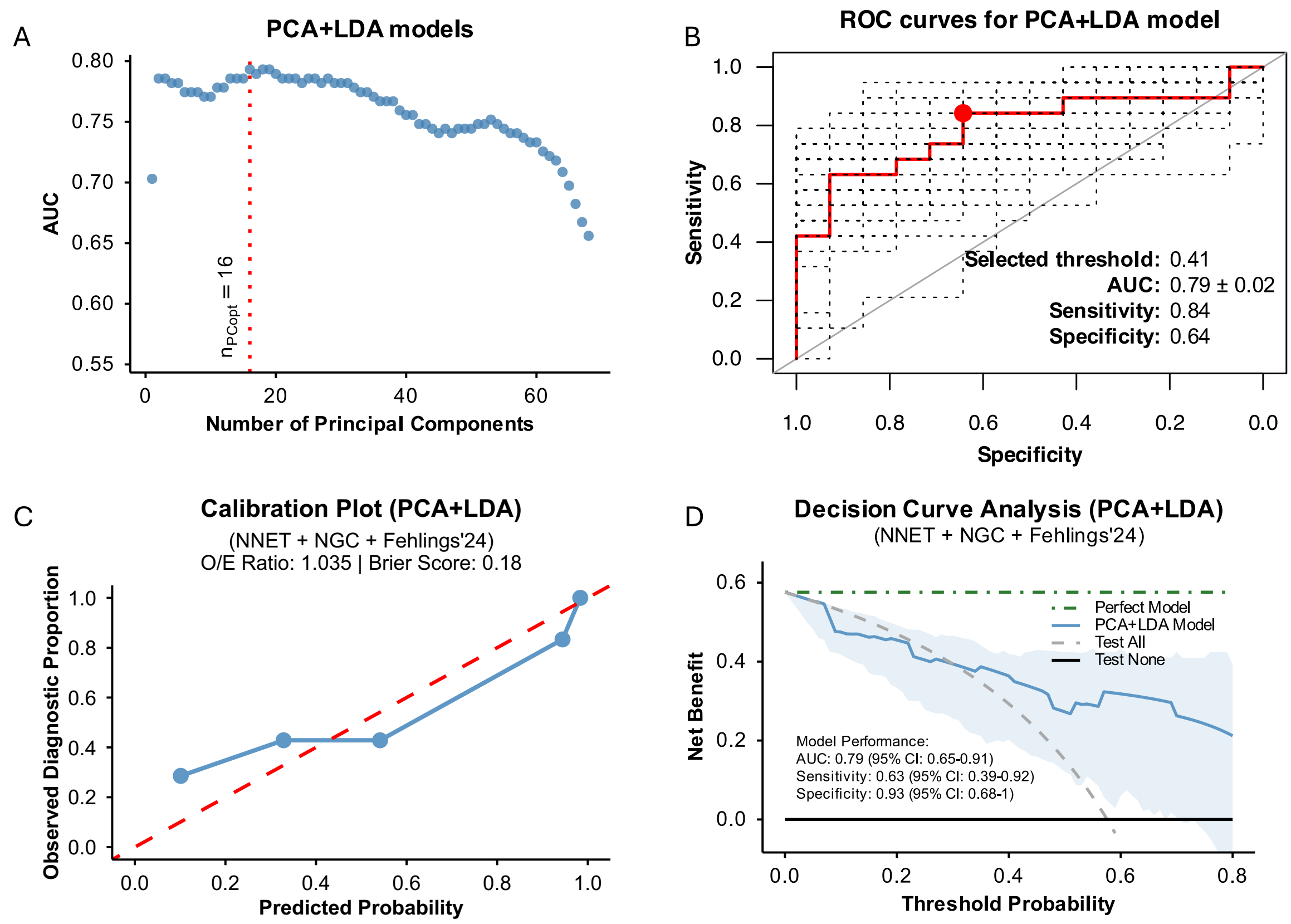

A: Median AUC as a function of the number of principal components (PCs) for the PCA+LDA models (NNET + NGC + Fehlings'24 cohorts). The red dotted line indicates the optimal number of PCs that maximized performance across the 100 resampling iterations used for principal component optimization. B: Receiver operating characteristic (ROC) analysis. Evaluations of the predictive performance of the optimized PCA+LDA model on the internal validation dataset (N=33). The individual validation curves are plotted across the identical 100 resampling iterations used for principal component optimization, from which the aggregate AUC (0.79 ± 0.02) and its confidence interval were estimated. The diagonal grey line indicates a random classifier baseline. A selected classification threshold (red dot) highlights the operational cutoff of 0.41, chosen specifically to prioritize clinical sensitivity and maximize case detection. At this specific sensitivity-optimized threshold, the model achieved a sensitivity of 84.2% (16/19) and a specificity of 64.3% (9/14). The absolute classification counts comprised 16 true positives, 9 true negatives, 5 false positives, and 3 false negatives. C: Calibration plot of the PCA+LDA model for the validation dataset. Observed diagnostic proportions are plotted against mean predicted probabilities across five bins/quantiles (light-blue points and line). The red dashed line represents perfect calibration. Overall model calibration and predictive accuracy are evaluated via the Brier score and Observed-to-Expected (O/E) ratio. D: Decision Curve Analysis (DCA) for the PCA+LDA model evaluated on the validation dataset. Net benefit curves are plotted across clinical threshold probabilities from 0 to 0.8. The model-guided strategy (solid light-blue line) is presented with its empirical 95% confidence ribbon (shaded light-blue area) against baseline clinical strategies: "Test All" (dashed dark-grey line), "Test None" (solid black line), and "Perfect Model" (dot-dashed green line). The inset text displays companion classification metrics with the operational threshold chosen via the Youden index. The empirical 95% confidence bounds (the 2.5th and 97.5th percentiles of the net benefit distributions) were calculated across the identical 100 resampling iterations used for principal component optimization to ensure strict consistency.

Supplementary Figure 8: Results for the PCA+GBM models (NNET+NGC+Fehlings'24 cohorts)

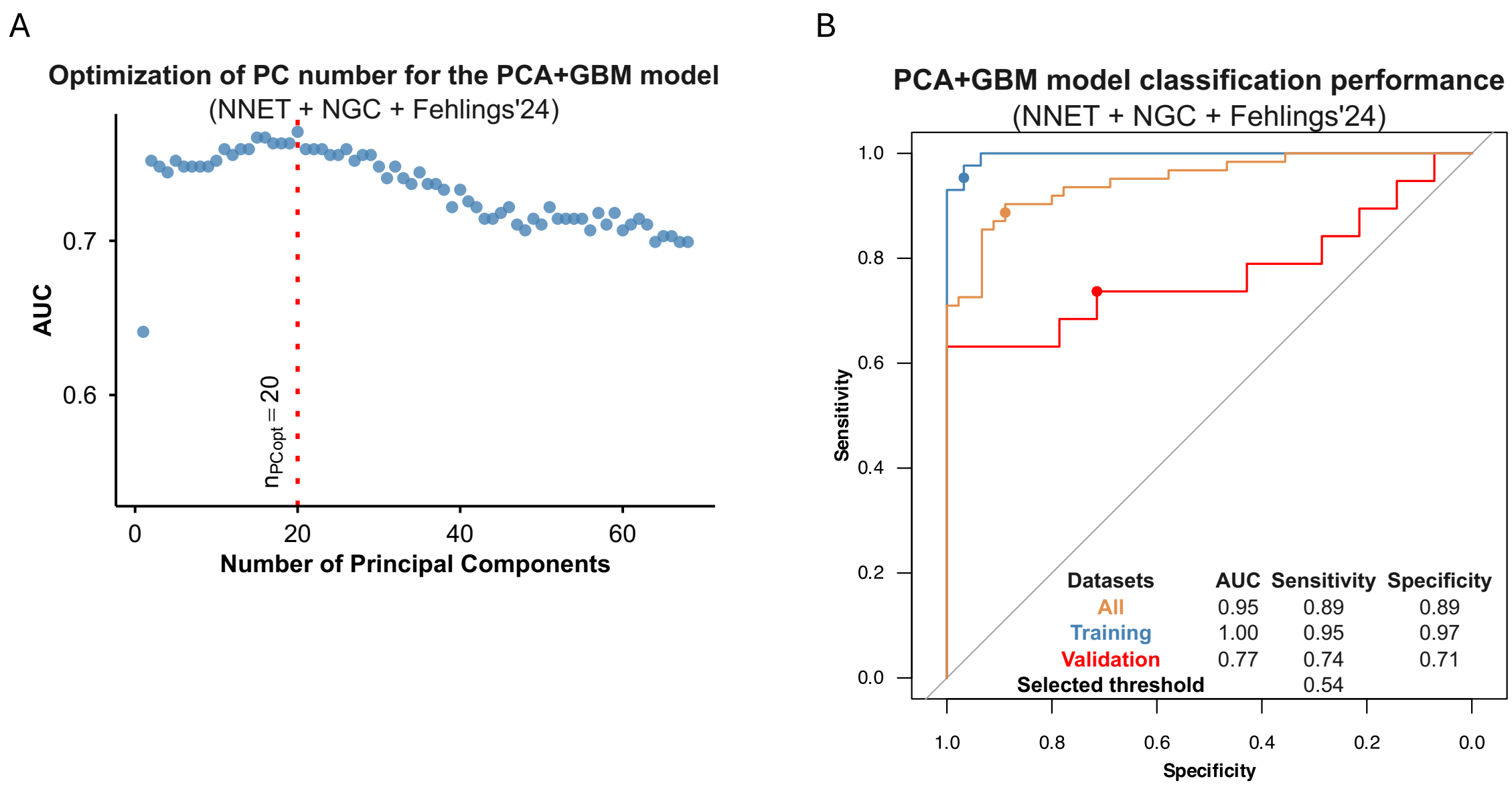

A: Average AUC as a function of the number of principal components (PCs) for the PCA+GBM models (NNET+NGC+Fehlings'24 cohorts). The red dashed line indicates the number of PCs that maximized the model's performance. B: ROC curves for the PCA+GBM model, developed using the optimal number of PCs. The curves for the full (orange), training (blue), and validation (red) datasets are shown. Each curve represents the bootstrap model iteration with an AUC closest to the average AUC from all iterations (as described in Supplementary Figure 5 and the "Phenotype-Based Genetic Diagnostic Model Pipeline" section). The marked point on each curve indicates the operational classification threshold. Rather than relying solely on the mathematical optimization of the Youden's J statistic (which weighs false positives and false negatives equally), this operational threshold was selected based on Decision Curve Analysis (DCA) profiles to optimize net clinical utility and ensure high diagnostic sensitivity. This clinically justified threshold balances the benefit of identifying positive cases against the risks of false positives and false negatives across modelled triage pathways. AUC: Area under the curve; GBM: Gradient Boosting Machine.

Supplementary Figure 9: HPO hierarchical levels histogram and clustering plot (NNET+NGC+Fehlings'24+GELCP)

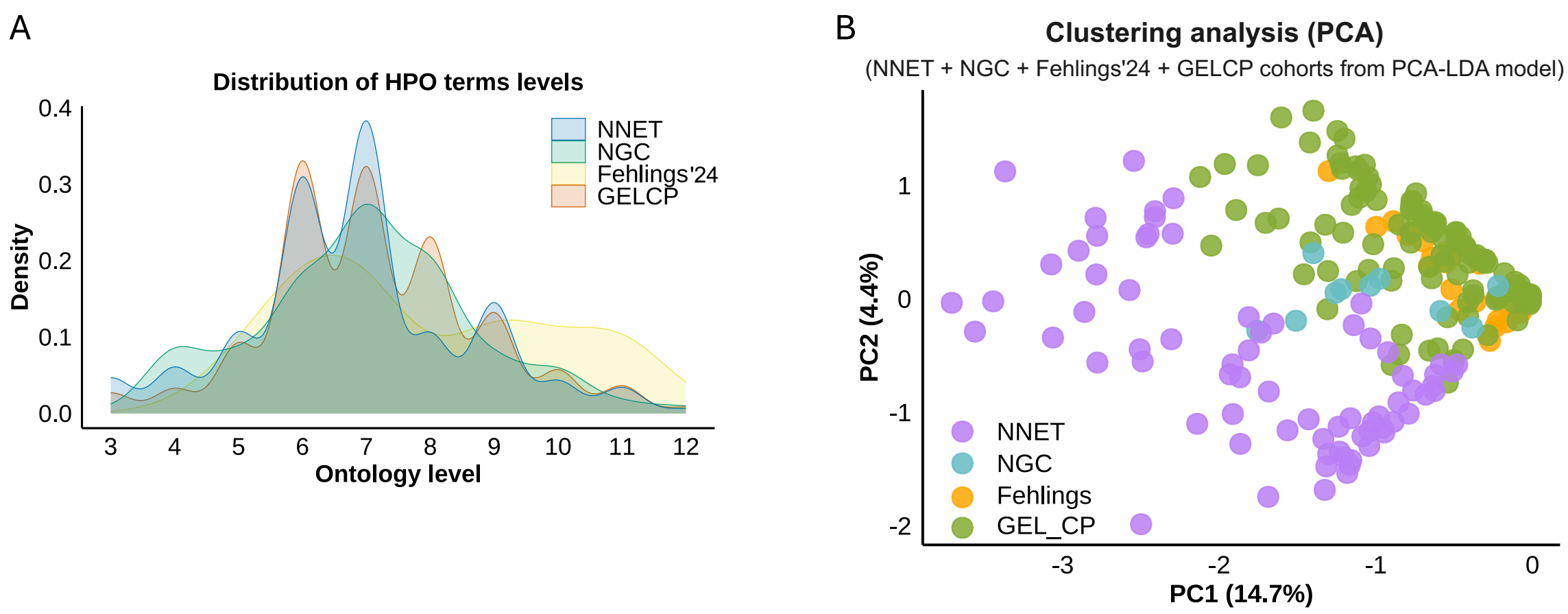

A: Distribution of HPO term depths across study cohorts. Density plot comparing the hierarchical levels of HPO terms for the NNET+NGC+ Fehlings'24+GELCP cohorts. The x-axis represents the ontology level, where higher values indicate more specific clinical phenotypes. For each original HPO term, the lowest possible level (minimum distance from the root) was identified across all potential paths within the ontology hierarchy using the ontologyIndex R package and a custom breadth-first search algorithm. The visualization was generated

using the ggplot2 R library. B: Scatter plot of PC1 versus PC2 scores for each case. These scores result from the dimensionality reduction of HPO terms described in Supplementary Figure 5 and “Phenotype-Based Genetic Diagnostic Model Pipeline” section in Supplementary Information. The represent a subset of the optimal number of principal components used as inputs for the PCA+LDA model from the NNET+NGC+ Fehlings’24+GELCP dataset. Cases are coloured by cohort: NNET (purple), NGC (light blue), Fehlings’24 (yellow), and GELCP (green).

Supplemental Tables

Supplementary Table 1: The Strengthening the Reporting of Observational Studies in Epidemiology (STROBE) Statement

	Item No	Recommendation	Page
**Title and abstract**	1	(*a*) Indicate the study's design with a commonly used term in the title or the abstract	1
		(*b*) Provide in the abstract an informative and balanced summary of what was done and what was found	3
**Introduction**			
Background/rationale	2	Explain the scientific background and rationale for the investigation being reported	4-5
Objectives	3	State specific objectives, including any prespecified hypotheses	4-5
**Methods**			
Study design	4	Present key elements of study design early in the paper	5
Setting	5	Describe the setting, locations, and relevant dates, including periods of recruitment, exposure, follow-up, and data collection	5-7
Participants	6	(*a*) *Cohort study*—Give the eligibility criteria, and the sources and methods of selection of participants. Describe methods of follow-up<br>*Case-control study*—Give the eligibility criteria, and the sources and methods of case ascertainment and control selection. Give the rationale for the choice of cases and controls<br>*Cross-sectional study*—Give the eligibility criteria, and the sources and methods of selection of participants	5
		(*b*) *Cohort study*—For matched studies, give matching criteria and number of exposed and unexposed<br>*Case-control study*—For matched studies, give matching criteria and the number of controls per case	
Variables	7	Clearly define all outcomes, exposures, predictors, potential confounders, and effect modifiers. Give diagnostic criteria, if applicable	N/A
Data sources/ measurement	8*	For each variable of interest, give sources of data and details of methods of assessment (measurement). Describe comparability of assessment methods if there is more than one group	6-7
Bias	9	Describe any efforts to address potential sources of bias	8-9
Study size	10	Explain how the study size was arrived at	N/A
Quantitative variables	11	Explain how quantitative variables were handled in the analyses. If applicable, describe which groupings were chosen and why	8-9
Statistical methods	12	(*a*) Describe all statistical methods, including those used to control for confounding	8-9
		(*b*) Describe any methods used to examine subgroups and interactions	9
		(*c*) Explain how missing data were addressed	N/A

		(*d*) *Cohort study*—If applicable, explain how loss to follow-up was addressed<br>*Case-control study*—If applicable, explain how matching of cases and controls was addressed<br>*Cross-sectional study*—If applicable, describe analytical methods taking account of sampling strategy	N/A
		(*e*) Describe any sensitivity analyses	N/A
**Results**			
Participants	13*	(a) Report numbers of individuals at each stage of study—eg numbers potentially eligible, examined for eligibility, confirmed eligible, included in the study, completing follow-up, and analysed	11
		(b) Give reasons for non-participation at each stage	N/A
		(c) Consider use of a flow diagram	Figure 1
Descriptive data	14*	(a) Give characteristics of study participants (eg demographic, clinical, social) and information on exposures and potential confounders	Table 1
		(b) Indicate number of participants with missing data for each variable of interest	N/A
		(c) *Cohort study*—Summarise follow-up time (eg, average and total amount)	N/A
Outcome data	15*	*Cohort study*—Report numbers of outcome events or summary measures over time	
		Case-control study—Report numbers in each exposure category, or summary measures of exposure	
		Cross-sectional study—Report numbers of outcome events or summary measures	11, 13
Main results	16	(*a*) Give unadjusted estimates and, if applicable, confounder-adjusted estimates and their precision (eg, 95% confidence interval). Make clear which confounders were adjusted for and why they were included	15-16
		(*b*) Report category boundaries when continuous variables were categorized	N/A
		(*c*) If relevant, consider translating estimates of relative risk into absolute risk for a meaningful time period	N/A

Other analyses	17	Report other analyses done—eg analyses of subgroups and interactions, and sensitivity analyses	14-17
**Discussion**			
Key results	18	Summarise key results with reference to study objectives	17-20
Limitations	19	Discuss limitations of the study, taking into account sources of potential bias or imprecision. Discuss both direction and magnitude of any potential bias	20
Interpretation	20	Give a cautious overall interpretation of results considering objectives, limitations, multiplicity of analyses, results from similar studies, and other relevant evidence	21
Generalisability	21	Discuss the generalisability (external validity) of the study results	20-21
**Other information**			
Funding	22	Give the source of funding and the role of the funders for the present study and, if applicable, for the original study on which the present article is based	10

*Give information separately for cases and controls in case-control studies and, if applicable, for exposed and unexposed groups in cohort and cross-sectional studies. See: https://www.equator-network.org/reporting-guidelines/strobe/

Supplementary Table 2: CP gene list

HGNC symbol	Source	HGNC symbol	Source	HGNC symbol	Source
AARS1	Fehlings et al. (2024)	GABRB2	van Eyk et al. (2023); Fehlings et al. (2024)	PLK4	Fehlings et al. (2024)
AARS2	Fehlings et al. (2024)	GABRB3	Fehlings et al. (2024)	PLOD1	Fehlings et al. (2024)
AASS	Fehlings et al. (2024)	GABRG2	Fehlings et al. (2024)	PLP1	PanelApp Australia; Matthews et al. (2019); Suchowersky et al. (2021); Lee et al. (2021); van Eyk et al. (2023); Fehlings et al. (2024)
ABAT	Fehlings et al. (2024)	GAD1	Fehlings et al. (2024)	PLXNA2	Matthews et al. (2019); Fehlings et al. (2024)
ABCA4	van Eyk et al. (2021)	GALC	Fehlings et al. (2024); van Eyk et al. (2021)	PMM2	Sreedevi et al. (2023); van Eyk et al. (2023)
ABCC8	Fehlings et al. (2024)	GAMT	Fehlings et al. (2024)	PMP22	Fehlings et al. (2024)
ABCD1	Fehlings et al. (2024)	GAN	Fehlings et al. (2024)	PMPCA	Fehlings et al. (2024)
ABHD12	Fehlings et al. (2024)	GANAB	Fehlings et al. (2024)	PMS1	Fehlings et al. (2024)
ACAD9	Fehlings et al. (2024)	GARS1	May et al. (2021)	PNKD	Fehlings et al. (2024)
ACADM	PanelApp Australia; van Eyk et al. (2023)	GAS7	van Eyk et al. (2021)	PNKP	Fehlings et al. (2024)
ACAT2	Fehlings et al. (2024)	GATAD2B	Fehlings et al. (2024)	PNP	Fehlings et al. (2024)
ACO2	Fehlings et al. (2024)	GATM	Fehlings et al. (2024)	PNPLA4	van Eyk et al. (2021)
ACOX1	Fehlings et al. (2024)	GBA1	Fehlings et al. (2024)	PNPLA6	Zahrani et al. (2022); Suchowersky et al. (2021); van Eyk et al. (2023); Fehlings et al. (2024); van Eyk et al. (2021)
ACP2	Fehlings et al. (2024)	GBA2	Zahrani et al. (2022); Fehlings et al. (2024)	PNPLA7	Fehlings et al. (2024)
ACP5	Fehlings et al. (2024)	GBE1	Fehlings et al. (2024)	PNPLA8	Fehlings et al. (2024)
ACSL4	Fehlings et al. (2024)	GCDH	PanelApp Australia; Matthews et al. (2019); van Eyk et al. (2023); Fehlings et al. (2024)	PNPO	Fehlings et al. (2024)
ACTA1	Fehlings et al. (2024)	GCH1	PanelApp Australia; Fehlings et al. (2024)	PNPT1	van Eyk et al. (2023); Fehlings et al. (2024)
ACTB	PanelApp Australia; van Eyk et al. (2023); Fehlings et al. (2024)	GCK	Fehlings et al. (2024)	POGZ	van Eyk et al. (2023); Fehlings et al. (2024)
ACTG1	Fehlings et al. (2024)	GCSH	Fehlings et al. (2024)	POLA1	Fehlings et al. (2024)
ACTN1	van Eyk et al. (2021)	GDF6	Fehlings et al. (2024)	POLG	Fehlings et al. (2024)
ACY1	Fehlings et al. (2024)	GEMIN4	Fehlings et al. (2024)	POLR1C	Fehlings et al. (2024)
ADA2	Fehlings et al. (2024)	GFAP	Fehlings et al. (2024)	POLR1D	Fehlings et al. (2024)
ADAM22	Fehlings et al. (2024)	GFM1	Fehlings et al. (2024)	POLR2A	Chopra et al. (2022)
ADAR	PanelApp Australia; Jones et al. (2021); van Eyk et al. (2023); Fehlings et al. (2024)	GFM2	Fehlings et al. (2024)	POLR3A	Fehlings et al. (2024)
ADAT3	PanelApp Australia; Chopra et al. (2022); Yechieli et al. (2022); van Eyk et al. (2023)	GGA1	Fehlings et al. (2024)	POLR3B	Fehlings et al. (2024)
COQ8A	Zahrani et al. (2022)	GJA1	Fehlings et al. (2024)	POMGNT1	PanelApp Australia; van Eyk et al. (2023); Fehlings et al. (2024)
ADCY5	PanelApp Australia; van Eyk et al. (2023); Fehlings et al. (2024)	GJB1	Fehlings et al. (2024)	POMGNT2	Fehlings et al. (2024)
ADCY6	van Eyk et al. (2021)	GJB2	van Eyk et al. (2021)	POMK	Fehlings et al. (2024)

ADD3	PanelApp Australia; ClinGen CP GCEP; van Eyk et al. (2023); Fehlings et al. (2024)	GJC2	Fehlings et al. (2024)	POMT1	Fehlings et al. (2024)
ADGRG1	Fehlings et al. (2024)	GLB1	Fehlings et al. (2024)	POMT2	Fehlings et al. (2024)
ADK	Fehlings et al. (2024)	GLDC	Fehlings et al. (2024)	PON1	Fehlings et al. (2024)
ADNP	PanelApp Australia; van Eyk et al. (2023); Fehlings et al. (2024)	GLE1	Fehlings et al. (2024)	PON2	Fehlings et al. (2024)
ADRA2B	Fehlings et al. (2024)	GLI2	Fehlings et al. (2024)	PON3	Fehlings et al. (2024)
ADRB2	Fehlings et al. (2024)	GLI3	Fehlings et al. (2024)	PPARGC1A	Fehlings et al. (2024)
ADSL	Fehlings et al. (2024)	GLRA1	Fehlings et al. (2024)	PPM1D	Fehlings et al. (2024)
AFG2A	PanelApp Australia; Rosello et al. (2021)	GLRB	Fehlings et al. (2024)	PPP1R15B	Fehlings et al. (2024)
AFG2B	PanelApp Australia	GLRX5	Fehlings et al. (2024)	PPP2R1A	Fehlings et al. (2024)
AFG3L2	Fehlings et al. (2024)	GLUL	Fehlings et al. (2024)	PPP2R5D	Fehlings et al. (2024)
AGA	Fehlings et al. (2024)	GLYCTK	Fehlings et al. (2024)	PPP3CA	Fehlings et al. (2024)
AGAP1	Fehlings et al. (2024)	GM2A	Fehlings et al. (2024)	PPT1	May et al. (2021); Fehlings et al. (2024)
AGAP2	van Eyk et al. (2021)	GMPPB	Fehlings et al. (2024)	PQBP1	Fehlings et al. (2024)
AGTR2	Fehlings et al. (2024)	GNA11	Fehlings et al. (2024)	PRDM8	Fehlings et al. (2024)
AHDC1	PanelApp Australia; van Eyk et al. (2023); Fehlings et al. (2024); van Eyk et al. (2021)	GNAL	Fehlings et al. (2024)	PRICKLE1	Fehlings et al. (2024)
AHI1	Fehlings et al. (2024)	GNAO1	PanelApp Australia; May et al. (2021); Rosello et al. (2021); Zahrani et al. (2022); Chopra et al. (2022); Matthews et al. (2019); Vasconcellos et al. (2023); Malaquias et al. (2019); van Eyk et al. (2023); Fehlings et al. (2024); Fehlings et al. (2024); van Eyk et al. (2021)	PRKCG	Fehlings et al. (2024)
AIFM1	Yechieli et al. (2022); Fehlings et al. (2024)	GNAS	Fehlings et al. (2024)	PRKDC	Fehlings et al. (2024)
AIMP1	Fehlings et al. (2024)	GNB1	PanelApp Australia; Rosello et al. (2021); van Eyk et al. (2023); van Eyk et al. (2023); Fehlings et al. (2024); van Eyk et al. (2021)	PRKRA	Fehlings et al. (2024)
AIMP2	Fehlings et al. (2024)	GOSR2	Fehlings et al. (2024)	PRMT7	Zahrani et al. (2022)
AIP	Fehlings et al. (2024)	GPAM	Fehlings et al. (2024)	PRNP	Fehlings et al. (2024)
AIPL1	Fehlings et al. (2024)	GPBP1	Fehlings et al. (2024)	PROC	PanelApp Australia; van Eyk et al. (2023); Fehlings et al. (2024); Fehlings et al. (2024); van Eyk et al. (2021)
AKT1	Fehlings et al. (2024)	GPC3	Fehlings et al. (2024)	PRPH	Fehlings et al. (2024)
AKT3	Matthews et al. (2019); van Eyk et al. (2023); Fehlings et al. (2024)	GPHN	Fehlings et al. (2024)	PRRT2	Zahrani et al. (2022); Fehlings et al. (2024)
ALDH18A1	Fehlings et al. (2024)	GPR101	Fehlings et al. (2024)	PRUNE1	PanelApp Australia; Zahrani et al. (2022); van Eyk et al. (2023); Fehlings et al. (2024)
ALDH3A2	PanelApp Australia; van Eyk et al. (2023); van Eyk et al. (2023); Fehlings et al. (2024); van Eyk et al. (2021)	GPR137C	Fehlings et al. (2024)	PSAP	Fehlings et al. (2024)
ALDH5A1	Fehlings et al. (2024)	GPR35	Fehlings et al. (2024)	PSAT1	Fehlings et al. (2024)
ALDH6A1	Fehlings et al. (2024)	GPR88	Fehlings et al. (2024)	PSD	Fehlings et al. (2024)
ALDH7A1	Fehlings et al. (2024)	ADGRV1	Fehlings et al. (2024)	PSEN1	Fehlings et al. (2024)

ALG11	Fehlings et al. (2024)	GPSM2	Fehlings et al. (2024)	PTCHD1	Fehlings et al. (2024)
ALG13	Fehlings et al. (2024)	GPT2	Fehlings et al. (2024)	PTEN	Fehlings et al. (2024)
ALG2	Fehlings et al. (2024)	GPX4	Fehlings et al. (2024)	PTK2B	Fehlings et al. (2024)
ALG3	Fehlings et al. (2024)	GRAMD1B	Fehlings et al. (2024)	PTK7	Fehlings et al. (2024)
ALG6	Zahrani et al. (2022); Fehlings et al. (2024)	GRIA3	Fehlings et al. (2024)	PTPN11	van Eyk et al. (2023); Fehlings et al. (2024)
ALK	Jin et al. (2020); van Eyk et al. (2023); Fehlings et al. (2024)	GRID2	Fehlings et al. (2024); Fehlings et al. (2024)	PTPN23	van Eyk et al. (2023); Fehlings et al. (2024)
ALKBH3	Fehlings et al. (2024)	GRIK2	Fehlings et al. (2024)	PTPRM	Fehlings et al. (2024)
ALS2	PanelApp Australia; van Eyk et al. (2023); Fehlings et al. (2024)	GRIN1	PanelApp Australia; van Eyk et al. (2023); Fehlings et al. (2024)	PTS	Fehlings et al. (2024)
AMACR	Fehlings et al. (2024)	GRIN2A	Fehlings et al. (2024)	PURA	PanelApp Australia; van Eyk et al. (2023); Fehlings et al. (2024)
AMER1	Fehlings et al. (2024)	GRIN2B	PanelApp Australia; van Eyk et al. (2023); van Eyk et al. (2023); Fehlings et al. (2024); van Eyk et al. (2021)	PUS3	Fehlings et al. (2024)
AMMECR1	Fehlings et al. (2024)	GRIN2D	Fehlings et al. (2024)	PYCR2	Fehlings et al. (2024)
AMPD1	Fehlings et al. (2024)	GRIP1	Fehlings et al. (2024)	QARS1	Fehlings et al. (2024)
AMPD2	PanelApp Australia; van Eyk et al. (2023); Fehlings et al. (2024); Fehlings et al. (2024)	GRK2	Fehlings et al. (2024)	QDPR	Fehlings et al. (2024)
AMT	Fehlings et al. (2024)	GRSF1	Fehlings et al. (2024)	QKI	Fehlings et al. (2024)
ANG	Fehlings et al. (2024)	GSS	Fehlings et al. (2024)	RAB11A	Fehlings et al. (2024)
ANK3	Fehlings et al. (2024)	GSX2	ClinGen CP GCEP; Fehlings et al. (2024)	RAB12	Fehlings et al. (2024)
ANKLE2	Fehlings et al. (2024); van Eyk et al. (2021)	GTF2IRD1	Fehlings et al. (2024)	RAB18	Fehlings et al. (2024)
ANKRD11	Fehlings et al. (2024)	GTPBP3	Fehlings et al. (2024)	RAB27A	Fehlings et al. (2024)
ANO10	Fehlings et al. (2024)	GUCY2D	Fehlings et al. (2024)	RAB3GAP1	PanelApp Australia; van Eyk et al. (2023); Fehlings et al. (2024)
ANO3	Fehlings et al. (2024)	GUF1	Fehlings et al. (2024)	RAB3GAP2	Fehlings et al. (2024)
ANO5	Fehlings et al. (2024)	H1-4	Fehlings et al. (2024)	RAB5C	van Eyk et al. (2021)
AP1S2	Fehlings et al. (2024); Fehlings et al. (2024)	HACE1	Fehlings et al. (2024)	RABEP1	Fehlings et al. (2024)
AP2M1	Yechieli et al. (2022)	HADH	Fehlings et al. (2024)	RAD21	Fehlings et al. (2024)
AP3B2	Fehlings et al. (2024)	HCFC1	Fehlings et al. (2024)	RAD50	Fehlings et al. (2024)
AP3D1	Fehlings et al. (2024)	HCN1	Fehlings et al. (2024)	RAG1	Fehlings et al. (2024)
AP4B1	PanelApp Australia; Rosello et al. (2021); van Eyk et al. (2023); Fehlings et al. (2024)	HDAC8	Fehlings et al. (2024)	RAI1	Fehlings et al. (2024)
AP4E1	PanelApp Australia; van Eyk et al. (2023); Fehlings et al. (2024)	HECTD1	Fehlings et al. (2024)	RANBP2	Matthews et al. (2019); Fehlings et al. (2024)
AP4M1	PanelApp Australia; van Eyk et al. (2023); Fehlings et al. (2024); Fehlings et al. (2024)	HECW1	Fehlings et al. (2024)	RAP2C	Fehlings et al. (2024)
AP4S1	PanelApp Australia; van Eyk et al. (2023); Fehlings et al. (2024)	HECW2	PanelApp Australia; van Eyk et al. (2023); Fehlings et al. (2024)	RAPGEFL1	van Eyk et al. (2021)
AP5Z1	ClinGen CP GCEP; Fehlings et al. (2024)	HEPACAM	Fehlings et al. (2024)	RARS2	PanelApp Australia; van Eyk et al. (2023); Fehlings et al. (2024)
APOE	Fehlings et al. (2024)	HERC1	Fehlings et al. (2024)	RASGEF1B	van Eyk et al. (2021)

APTX	Zahrani et al. (2022); Fehlings et al. (2024)	HERC2	van Eyk et al. (2021)	RAX	Fehlings et al. (2024)
ARCN1	Fehlings et al. (2024)	HIBCH	Fehlings et al. (2024)	RBPJ	Fehlings et al. (2024)
ARFGAP2	van Eyk et al. (2021)	HIC1	Fehlings et al. (2024)	RD3	Fehlings et al. (2024)
ARFGEF2	Fehlings et al. (2024)	HIKESHI	Fehlings et al. (2024)	RDH12	Fehlings et al. (2024)
ARG1	PanelApp Australia; Mills et al. (2023); van Eyk et al. (2023); Fehlings et al. (2024)	H1-4	Fehlings et al. (2024)	RECQL4	Fehlings et al. (2024)
ARHGAP31	van Eyk et al. (2023); Fehlings et al. (2024); van Eyk et al. (2021)	HIVEP2	Fehlings et al. (2024)	REEP1	Yechieli et al. (2022); Fehlings et al. (2024)
ARHGAP32	van Eyk et al. (2021)	HNMT	Fehlings et al. (2024)	REEP2	Fehlings et al. (2024)
ARHGEF15	Fehlings et al. (2024)	HNRNPA1	Fehlings et al. (2024)	RELN	Zahrani et al. (2022); Fehlings et al. (2024)
ARHGEF9	Fehlings et al. (2024)	HNRNPH2	Fehlings et al. (2024)	REM2	van Eyk et al. (2021)
ARID1A	Fehlings et al. (2024)	HNRNPL	Fehlings et al. (2024)	RETREG1	Fehlings et al. (2024)
ARID1B	Fehlings et al. (2024)	HNRNPU	Fehlings et al. (2024)	RFC2	Fehlings et al. (2024)
ARID2	PanelApp Australia; van Eyk et al. (2023); Fehlings et al. (2024)	HOOK3	Fehlings et al. (2024)	RFPL2	Fehlings et al. (2024)
ARL13B	Fehlings et al. (2024)	HPCA	Fehlings et al. (2024)	RFT1	Fehlings et al. (2024)
ARL6IP1	ClinGen CP GCEP; Fehlings et al. (2024)	HPDL	PanelApp Australia; van Eyk et al. (2023); Fehlings et al. (2024)	RFX2	Fehlings et al. (2024)
ARMC9	Fehlings et al. (2024)	HPGD	Fehlings et al. (2024)	RHOB	Jin et al. (2020); Fehlings et al. (2024)
ARNT2	Fehlings et al. (2024)	HPRT1	PanelApp Australia; van Eyk et al. (2023); Fehlings et al. (2024)	RIT1	Fehlings et al. (2024)
ARSA	Fehlings et al. (2024)	HSD17B10	Fehlings et al. (2024)	RMND1	Fehlings et al. (2024)
ARSL	Fehlings et al. (2024)	HSD17B4	Fehlings et al. (2024)	RNASEH2A	PanelApp Australia; van Eyk et al. (2023); Fehlings et al. (2024)
ARSI	Fehlings et al. (2024)	HSPA4	Fehlings et al. (2024)	RNASEH2B	PanelApp Australia; Rosello et al. (2021); Beysen et al. (2021); van Eyk et al. (2023); Fehlings et al. (2024)
ARSL	Fehlings et al. (2024)	HSPB3	Fehlings et al. (2024)	RNASEH2C	PanelApp Australia; van Eyk et al. (2023); Fehlings et al. (2024)
ARV1	Fehlings et al. (2024)	HSPD1	Fehlings et al. (2024)	RNASET2	Fehlings et al. (2024)
ARX	PanelApp Australia; Yechieli et al. (2022); van Eyk et al. (2023); Fehlings et al. (2024)	HSPG2	Fehlings et al. (2024)	RNF113A	Fehlings et al. (2024)
ASCC1	Fehlings et al. (2024)	HTRA1	Fehlings et al. (2024)	RNF125	Fehlings et al. (2024)
ASNS	Fehlings et al. (2024)	HTRA2	Fehlings et al. (2024); van Eyk et al. (2021)	RNF170	Fehlings et al. (2024)
ASPA	Fehlings et al. (2024)	HTT	van Eyk et al. (2021)	RNF216	Fehlings et al. (2024)
ASPM	Fehlings et al. (2024)	HUWE1	van Eyk et al. (2023); Fehlings et al. (2024)	ROBO1	Fehlings et al. (2024)
ASTN2	Fehlings et al. (2024); van Eyk et al. (2021)	HYLS1	Fehlings et al. (2024)	ROCK2	van Eyk et al. (2021)
ASXL1	PanelApp Australia; Matthews et al. (2019); van Eyk et al. (2023); Fehlings et al. (2024)	IARS1	Fehlings et al. (2024)	ROGDI	Fehlings et al. (2024)
ASXL3	PanelApp Australia; Svantnerova et al. (2022); van Eyk et al. (2023); Fehlings et al. (2024)	IBA57	Fehlings et al. (2024)	RPE65	Fehlings et al. (2024)
ATAD3A	Fehlings et al. (2024)	ICAM5	van Eyk et al. (2021)	RPGRIP1	Fehlings et al. (2024)
ATAD3B	Fehlings et al. (2024)	IDH1	Fehlings et al. (2024)	RPGRIP1L	Fehlings et al. (2024)

ATCAY	Fehlings et al. (2024)	IDH2	Fehlings et al. (2024)	RPIA	Fehlings et al. (2024)
ATF4	Fehlings et al. (2024); van Eyk et al. (2021)	IDH3A	Fehlings et al. (2024)	RPS6KA3	Fehlings et al. (2024)
ATG7	Fehlings et al. (2024)	IDS	Fehlings et al. (2024)	RRM2B	Fehlings et al. (2024)
ATIC	Fehlings et al. (2024)	IDUA	Fehlings et al. (2024)	RSPH4A	Fehlings et al. (2024)
ATL1	PanelApp Australia; Rosello et al. (2021); Chopra et al. (2022); Yechieli et al. (2022); Suchowersky et al. (2021); Jin et al. (2020); van Eyk et al. (2023); Fehlings et al. (2024)	IER3IP1	Fehlings et al. (2024)	RTN2	Fehlings et al. (2024)
ATM	PanelApp Australia; May et al. (2021); Zahrani et al. (2022); van Eyk et al. (2023); Fehlings et al. (2024)	IFIH1	PanelApp Australia; Rosello et al. (2021); van Eyk et al. (2023); Fehlings et al. (2024)	RTN4IP1	Fehlings et al. (2024)
ATP13A2	Fehlings et al. (2024)	IFT140	Fehlings et al. (2024)	RTTN	Fehlings et al. (2024)
ATP1A2	Fehlings et al. (2024)	IFT172	Fehlings et al. (2024)	RXYLT1	Fehlings et al. (2024)
ATP1A3	PanelApp Australia; Matthews et al. (2019); van Eyk et al. (2023); Fehlings et al. (2024); van Eyk et al. (2021)	IKBKG	van Eyk et al. (2021)	RYR1	Fehlings et al. (2024)
ATP2B3	Fehlings et al. (2024)	IL10	Fehlings et al. (2024)	RYR2	Fehlings et al. (2024)
ATP2B4	Fehlings et al. (2024)	IL1B	Fehlings et al. (2024)	S100A9	Fehlings et al. (2024)
ATP5F1A	Fehlings et al. (2024)	IL1RAP	van Eyk et al. (2021)	SACS	PanelApp Australia; Suchowersky et al. (2021); van Eyk et al. (2023); Fehlings et al. (2024)
ATP6AP2	Fehlings et al. (2024)	IL1RAPL1	Fehlings et al. (2024)	SAMHD1	PanelApp Australia; Beysen et al. (2021); van Eyk et al. (2023); Fehlings et al. (2024)
ATP6V0A2	Fehlings et al. (2024)	IL6	Fehlings et al. (2024)	SARS2	Fehlings et al. (2024)
ATP6V0A4	Fehlings et al. (2024)	IMPDH1	Fehlings et al. (2024)	SASS6	Fehlings et al. (2024)
ATP6V1A	Fehlings et al. (2024)	INPP4A	Fehlings et al. (2024)	SATB2	PanelApp Australia; Chopra et al. (2022); van Eyk et al. (2023); Fehlings et al. (2024)
ATP6V1E1	Fehlings et al. (2024)	INPP5E	Fehlings et al. (2024)	SCARB2	Fehlings et al. (2024)
ATP7A	PanelApp Australia; van Eyk et al. (2023); Fehlings et al. (2024)	INPP5K	Fehlings et al. (2024)	SCN10A	Fehlings et al. (2024)
ATP7B	van Eyk et al. (2023); Fehlings et al. (2024)	INS	Fehlings et al. (2024)	SCN1A	PanelApp Australia; May et al. (2021); Zahrani et al. (2022); van Eyk et al. (2023); Fehlings et al. (2024); van Eyk et al. (2021)
ATP8A2	PanelApp Australia; van Eyk et al. (2023); Fehlings et al. (2024)	IQCB1	Fehlings et al. (2024)	SCN1B	Fehlings et al. (2024)
ATR	Fehlings et al. (2024)	IQSEC2	van Eyk et al. (2023); Fehlings et al. (2024)	SCN2A	PanelApp Australia; May et al. (2021); Zahrani et al. (2022); van Eyk et al. (2023); Fehlings et al. (2024)
ATRX	PanelApp Australia; van Eyk et al. (2023); Fehlings et al. (2024)	IRF2BPL	PanelApp Australia; van Eyk et al. (2023)	SCN3A	Matthews et al. (2019); Fehlings et al. (2024)
ATXN2	Fehlings et al. (2024)	ISCA2	Fehlings et al. (2024)	SCN3B	Fehlings et al. (2024)
AUH	Fehlings et al. (2024)	ITM2B	Fehlings et al. (2024)	SCN4A	Fehlings et al. (2024)
AUTS2	PanelApp Australia; van Eyk et al. (2023); Fehlings et al. (2024)	ITPA	Fehlings et al. (2024)	SCN8A	PanelApp Australia; van Eyk et al. (2023); Fehlings et al. (2024); Fehlings et al. (2024); van Eyk et al. (2021)

AXL	van Eyk et al. (2021)	ITPR1	PanelApp Australia; van Eyk et al. (2023); Fehlings et al. (2024); van Eyk et al. (2021)	SCO1	Fehlings et al. (2024); van Eyk et al. (2021)
B3GALNT2	Fehlings et al. (2024)	ITPR3	van Eyk et al. (2021)	SCP2	Fehlings et al. (2024)
B3GNT2	Fehlings et al. (2024)	JAM3	Fehlings et al. (2024)	SCRIB	Fehlings et al. (2024)
B4GALNT1	ClinGen CP GCEP; Fehlings et al. (2024)	KDM7A	Fehlings et al. (2024)	SCYL1	Fehlings et al. (2024)
B4GAT1	Fehlings et al. (2024)	KANK1	Matthews et al. (2019); Fehlings et al. (2024); van Eyk et al. (2021)	SDHA	Fehlings et al. (2024)
B9D1	Fehlings et al. (2024)	KANSL1	Fehlings et al. (2024)	SDHAF1	Fehlings et al. (2024)
B9D2	Fehlings et al. (2024)	KARS1	Fehlings et al. (2024)	SDHB	Fehlings et al. (2024)
BAZ1B	Fehlings et al. (2024)	KAT6A	PanelApp Australia; van Eyk et al. (2023); Fehlings et al. (2024); van Eyk et al. (2021)	SDHD	Fehlings et al. (2024)
BCAP31	PanelApp Australia; van Eyk et al. (2023); Fehlings et al. (2024)	KAT6B	Fehlings et al. (2024)	SEC24B	Fehlings et al. (2024)
BCL11A	PanelApp Australia; van Eyk et al. (2023); Fehlings et al. (2024)	KATNB1	Fehlings et al. (2024)	SEMA4A	Fehlings et al. (2024)
BCL11B	Fehlings et al. (2024)	KATNIP	Fehlings et al. (2024)	SEMA4C	Fehlings et al. (2024)
BCOR	Fehlings et al. (2024); van Eyk et al. (2021)	KAZALD1	van Eyk et al. (2021)	SEMA4D	Fehlings et al. (2024)
BDNF	Fehlings et al. (2024)	KCNA1	Fehlings et al. (2024)	SEMA5A	Fehlings et al. (2024)
BRAT1	van Eyk et al. (2023)	KCNA2	Fehlings et al. (2024)	SEPSECS	PanelApp Australia; van Eyk et al. (2023); Fehlings et al. (2024)
BSCL2	van Eyk et al. (2021)	KCNA6	van Eyk et al. (2021)	SERAC1	Fehlings et al. (2024)
BUB1B	Fehlings et al. (2024); van Eyk et al. (2021)	KCNAB1	Fehlings et al. (2024)	SERPINI1	Fehlings et al. (2024)
C19orf12	Fehlings et al. (2024)	KCNB1	PanelApp Australia; van Eyk et al. (2023); Fehlings et al. (2024)	SETBP1	Fehlings et al. (2024)
C2CD3	Fehlings et al. (2024)	KCNC1	Fehlings et al. (2024)	SETD2	Fehlings et al. (2024)
CPLANE1	Fehlings et al. (2024)	KCNC3	PanelApp Australia; van Eyk et al. (2023); Fehlings et al. (2024)	SETD5	Fehlings et al. (2024)
CA8	Fehlings et al. (2024)	KCND3	Fehlings et al. (2024)	SETX	van Eyk et al. (2023); Fehlings et al. (2024); van Eyk et al. (2021)
CACNA1A	PanelApp Australia; Zahrani et al. (2022); van Eyk et al. (2023); van Eyk et al. (2023); Fehlings et al. (2024); van Eyk et al. (2021)	KCNE5	Fehlings et al. (2024)	SF3B4	Fehlings et al. (2024)
CACNA1B	ClinGen CP GCEP; Fehlings et al. (2024)	KCNH1	Fehlings et al. (2024); van Eyk et al. (2021)	SGCE	Fehlings et al. (2024)
CACNA1C	van Eyk et al. (2021)	KCNH5	van Eyk et al. (2021)	SH2D1A	Fehlings et al. (2024)
CACNA1D	Fehlings et al. (2024)	KCNJ10	Fehlings et al. (2024)	SH3GL2	Fehlings et al. (2024)
CACNA1E	Fehlings et al. (2024)	KCNJ11	Fehlings et al. (2024)	SHANK3	Zahrani et al. (2022); van Eyk et al. (2023); Fehlings et al. (2024)
CACNA1G	PanelApp Australia; Fehlings et al. (2024)	KCNJ13	Fehlings et al. (2024)	SHOC2	Fehlings et al. (2024)
CACNA1S	van Eyk et al. (2021)	KCNJ6	Matthews et al. (2019); Fehlings et al. (2024)	SHROOM4	Fehlings et al. (2024)
CACNA2D1	Fehlings et al. (2024)	KCNJ9	Fehlings et al. (2024)	SIGMAR1	Fehlings et al. (2024)
CACNA2D2	Fehlings et al. (2024)	KCNK9	Fehlings et al. (2024)	SIK1	Fehlings et al. (2024)
CACNA2D3	Fehlings et al. (2024)	KCNMA1	Fehlings et al. (2024)	SIL1	Fehlings et al. (2024); Fehlings et al. (2024)
CAD	Fehlings et al. (2024)	KCNQ2	PanelApp Australia; van Eyk et al. (2023); Fehlings et al. (2024)	SIN3A	Fehlings et al. (2024)

CALM1	Fehlings et al. (2024)	KCNQ3	Fehlings et al. (2024)	SIRT1	Fehlings et al. (2024)
CAMTA1	PanelApp Australia; van Eyk et al. (2023); Fehlings et al. (2024); van Eyk et al. (2021)	KCNQ5	Fehlings et al. (2024)	SIX6	Fehlings et al. (2024)
CAMTA2	Fehlings et al. (2024)	KCNT1	PanelApp Australia; van Eyk et al. (2023); Fehlings et al. (2024)	SKI	van Eyk et al. (2021)
CAPN1	Fehlings et al. (2024)	KCTD13	Fehlings et al. (2024)	SLC12A5	Fehlings et al. (2024)
CAPN3	Fehlings et al. (2024)	KCTD17	Fehlings et al. (2024); van Eyk et al. (2021)	SLC13A5	Zahrani et al. (2022); van Eyk et al. (2023); Fehlings et al. (2024)
CARS2	Fehlings et al. (2024)	KCTD7	Fehlings et al. (2024)	SLC16A2	PanelApp Australia; Chopra et al. (2022); van Eyk et al. (2023); Fehlings et al. (2024)
CASK	PanelApp Australia; van Eyk et al. (2023); Fehlings et al. (2024)	KDM5A	Fehlings et al. (2024)	SLC17A5	Fehlings et al. (2024)
CASR	Fehlings et al. (2024)	KDM5C	PanelApp Australia; van Eyk et al. (2023); Fehlings et al. (2024)	SLC18A2	Fehlings et al. (2024)
CAV1	Fehlings et al. (2024)	KDM6A	Fehlings et al. (2024)	SLC19A3	Fehlings et al. (2024)
CC2D1A	Fehlings et al. (2024)	KDM6B	Fehlings et al. (2024)	SLC1A2	Fehlings et al. (2024)
CC2D2A	Fehlings et al. (2024)	KDM7A	Fehlings et al. (2024)	SLC1A4	Fehlings et al. (2024)
CCBE1	Fehlings et al. (2024)	KIAA0586	Fehlings et al. (2024)	SLC20A2	Fehlings et al. (2024)
CCDC22	Fehlings et al. (2024)	NEXMIF	Fehlings et al. (2024)	SLC22A5	Fehlings et al. (2024)
CCDC88A	Fehlings et al. (2024)	KIDINS220	PanelApp Australia; van Eyk et al. (2023); van Eyk et al. (2023); Fehlings et al. (2024); van Eyk et al. (2021)	SLC25A1	Fehlings et al. (2024)
CCDC88C	Fehlings et al. (2024); van Eyk et al. (2021)	KIF11	Fehlings et al. (2024)	SLC25A12	Fehlings et al. (2024)
CCN2	Fehlings et al. (2024)	KIF1A	PanelApp Australia; van Eyk et al. (2023); Fehlings et al. (2024)	SLC25A15	Fehlings et al. (2024)
CCND2	Fehlings et al. (2024)	KIF1C	Fehlings et al. (2024)	SLC25A19	Fehlings et al. (2024)
CCNF	Fehlings et al. (2024)	KIF23	Fehlings et al. (2024)	SLC25A20	Fehlings et al. (2024)
CCT5	Fehlings et al. (2024)	KIF2A	Fehlings et al. (2024)	SLC25A22	Fehlings et al. (2024)
CD99L2	Fehlings et al. (2024)	KIF5A	Fehlings et al. (2024)	SLC25A23	Fehlings et al. (2024)
CDC42	Fehlings et al. (2024)	KIF5C	Fehlings et al. (2024)	SLC26A1	Fehlings et al. (2024)
CDC42BPB	Fehlings et al. (2024)	KIF7	Fehlings et al. (2024)	SLC26A4	Fehlings et al. (2024)
CDK13	Fehlings et al. (2024); van Eyk et al. (2021)	KIFBP	Fehlings et al. (2024)	SLC2A1	PanelApp Australia; van Eyk et al. (2023); Fehlings et al. (2024); Fehlings et al. (2024); Fehlings et al. (2024); van Eyk et al. (2021)
CDK5	Fehlings et al. (2024)	KLC2	Fehlings et al. (2024)	SLC2A3	Fehlings et al. (2024)
CDK5RAP1	Fehlings et al. (2024)	KLC4	Fehlings et al. (2024)	SLC30A10	Fehlings et al. (2024)
CDK5RAP2	Fehlings et al. (2024)	KLF13	Fehlings et al. (2024)	SLC33A1	Fehlings et al. (2024)
CDK6	Fehlings et al. (2024)	KLHL15	Fehlings et al. (2024)	SLC35A2	Fehlings et al. (2024)
CDKL5	van Eyk et al. (2023); Fehlings et al. (2024)	KLHL3	van Eyk et al. (2023); van Eyk et al. (2021)	SLC35A3	Fehlings et al. (2024)
CDNF	van Eyk et al. (2021)	KMT2A	PanelApp Australia; van Eyk et al. (2023); Fehlings et al. (2024)	SLC35C1	Zahrani et al. (2022); Fehlings et al. (2024)
CENPE	Fehlings et al. (2024)	KMT2B	PanelApp Australia; van Eyk et al. (2023); Fehlings et al. (2024); van Eyk et al. (2021)	SLC35F5	Fehlings et al. (2024)

CPAP	Fehlings et al. (2024)	KMT2C	Fehlings et al. (2024)	SLC39A14	Fehlings et al. (2024)
CEP104	Fehlings et al. (2024)	KMT2D	Zahrani et al. (2022); Fehlings et al. (2024); van Eyk et al. (2021)	SLC39A8	Fehlings et al. (2024)
CEP120	Fehlings et al. (2024)	KMT2E	van Eyk et al. (2021)	SLC46A1	Fehlings et al. (2024)
CEP135	Fehlings et al. (2024)	KNL1	Fehlings et al. (2024)	SLC4A11	Fehlings et al. (2024)
CEP152	Fehlings et al. (2024)	KRAS	Fehlings et al. (2024)	SLC52A2	Zahrani et al. (2022)
CEP290	Fehlings et al. (2024)	KY	Fehlings et al. (2024)	SLC5A4	Fehlings et al. (2024)
CEP41	Fehlings et al. (2024)	KYNU	Fehlings et al. (2024)	SLC5A6	PanelApp Australia; van Eyk et al. (2023)
CEP63	Fehlings et al. (2024)	L1CAM	PanelApp Australia; May et al. (2021); van Eyk et al. (2023); Fehlings et al. (2024)	SLC6A1	Fehlings et al. (2024)
CFAP410	Fehlings et al. (2024)	L2HGDH	Fehlings et al. (2024)	SLC6A17	Fehlings et al. (2024)
CFTR	van Eyk et al. (2021)	L3HYPDH	Fehlings et al. (2024)	SLC6A3	van Eyk et al. (2023); Fehlings et al. (2024)
CHAMP1	Fehlings et al. (2024)	LAGE3	Fehlings et al. (2024)	SLC6A4	Fehlings et al. (2024)
CHAT	Fehlings et al. (2024)	LAMA1	Fehlings et al. (2024)	SLC6A5	Fehlings et al. (2024)
CHCHD10	Fehlings et al. (2024)	LAMA2	Fehlings et al. (2024)	SLC6A8	Fehlings et al. (2024)
CHD2	Fehlings et al. (2024)	LAMB1	Fehlings et al. (2024)	SLC6A9	Fehlings et al. (2024)
CHD4	Fehlings et al. (2024)	LAMC3	Zahrani et al. (2022); Fehlings et al. (2024)	SLC7A2	Fehlings et al. (2024)
CHD7	Fehlings et al. (2024); van Eyk et al. (2021)	LAMP2	Fehlings et al. (2024)	SLC9A1	Fehlings et al. (2024)
CHD8	PanelApp Australia; van Eyk et al. (2023); Fehlings et al. (2024); van Eyk et al. (2021)	LARGE1	Fehlings et al. (2024)	SLC9A5	van Eyk et al. (2021)
CHD9	van Eyk et al. (2021)	LARP7	Fehlings et al. (2024)	SLC9A6	Fehlings et al. (2024)
CHEK2	Fehlings et al. (2024)	LCA5	Fehlings et al. (2024)	SLCO2A1	Fehlings et al. (2024)
CHMP1A	Fehlings et al. (2024)	LDB3	van Eyk et al. (2021)	SLCO6A1	Fehlings et al. (2024)
CHMP2B	Fehlings et al. (2024)	LETM1	Fehlings et al. (2024)	SLITRK2	PanelApp Australia; van Eyk et al. (2023)
CHN1	Fehlings et al. (2024)	LGI1	Fehlings et al. (2024)	SMARCA2	Fehlings et al. (2024)
CHRNA1	Matthews et al. (2019); Fehlings et al. (2024)	LIAS	Fehlings et al. (2024)	SMARCA4	Fehlings et al. (2024)
CHRNA2	Fehlings et al. (2024)	LIMK1	Fehlings et al. (2024)	SMARCB1	May et al. (2021); van Eyk et al. (2023); Fehlings et al. (2024)
CHRNA4	Fehlings et al. (2024)	LIPH	Fehlings et al. (2024)	SMARCE1	Fehlings et al. (2024)
CHRNA7	Fehlings et al. (2024); van Eyk et al. (2021)	LIPT1	Fehlings et al. (2024)	SMC1A	Fehlings et al. (2024)
CHRNB2	Fehlings et al. (2024); van Eyk et al. (2021)	LIPT2	Fehlings et al. (2024)	SMC3	Fehlings et al. (2024)
CHRNE	Fehlings et al. (2024)	LMAN2L	Fehlings et al. (2024)	SMN1	Fehlings et al. (2024)
CIT	Fehlings et al. (2024)	LMNB1	Fehlings et al. (2024)	SMO	Fehlings et al. (2024)
CIZ1	Fehlings et al. (2024)	LMNB2	Fehlings et al. (2024)	SMOC1	Fehlings et al. (2024)
CKAP2L	Fehlings et al. (2024)	LOX	Fehlings et al. (2024)	SMPD1	Fehlings et al. (2024)
CLCN1	van Eyk et al. (2021)	LRAT	Fehlings et al. (2024)	SNAP25	Fehlings et al. (2024)
CLCN2	Fehlings et al. (2024); van Eyk et al. (2021)	LRFN2	Fehlings et al. (2024)	SNAP29	Fehlings et al. (2024)
CLCN4	van Eyk et al. (2023); Fehlings et al. (2024)	LRRC30	Fehlings et al. (2024)	SNCA	Fehlings et al. (2024)
CLIC2	Fehlings et al. (2024)	LTBP1	Fehlings et al. (2024)	SNCAIP	Fehlings et al. (2024)
CLIP2	Fehlings et al. (2024); van Eyk et al. (2021)	LYRM7	Fehlings et al. (2024)	SNX14	van Eyk et al. (2023); Fehlings et al. (2024)
CLN3	Fehlings et al. (2024)	MAFB	Fehlings et al. (2024)	SOBP	Fehlings et al. (2024)

CLN5	Fehlings et al. (2024)	MAG	ClinGen CP GCEP; Fehlings et al. (2024)	SOD1	Fehlings et al. (2024)
CLN6	Fehlings et al. (2024)	MAGI2	Fehlings et al. (2024)	SON	van Eyk et al. (2023); Fehlings et al. (2024)
CLN8	Fehlings et al. (2024)	MAN2B1	Fehlings et al. (2024)	SOS1	Fehlings et al. (2024)
CLP1	Fehlings et al. (2024)	MAOB	Fehlings et al. (2024)	SOX10	Fehlings et al. (2024)
CLPB	Fehlings et al. (2024)	MAP2K1	PanelApp Australia; van Eyk et al. (2023); Fehlings et al. (2024)	SOX2	Fehlings et al. (2024)
CLTC	PanelApp Australia; van Eyk et al. (2023); Fehlings et al. (2024)	MAP3K12	Fehlings et al. (2024)	SOX6	Fehlings et al. (2024)
CLTCL1	Fehlings et al. (2024)	MAP3K4	Fehlings et al. (2024)	SPART	ClinGen CP GCEP; Fehlings et al. (2024)
CNKSR2	Zahrani et al. (2022); Fehlings et al. (2024); van Eyk et al. (2021)	MAP3K9	Fehlings et al. (2024)	SPAST	PanelApp Australia; May et al. (2021); Rosello et al. (2021); van Eyk et al. (2023); Chopra et al. (2022); Matthews et al. (2019); Suchowersky et al. (2021); Jin et al. (2020); van Eyk et al. (2023); Fehlings et al. (2024); van Eyk et al. (2021)
CNNM2	Fehlings et al. (2024)	MAPK10	Fehlings et al. (2024)	AFG2A	van Eyk et al. (2023); Fehlings et al. (2024)
CNTN4	Fehlings et al. (2024)	MAPK8IP3	Yechieli et al. (2022)	AFG2B	van Eyk et al. (2023)
CNTN6	Fehlings et al. (2024); van Eyk et al. (2021)	MAPT	Fehlings et al. (2024)	SPATA7	Fehlings et al. (2024)
CNTNAP2	Fehlings et al. (2024); van Eyk et al. (2021)	MARCHF6	Fehlings et al. (2024)	SPG11	PanelApp Australia; Yechieli et al. (2022); van Eyk et al. (2023); Fehlings et al. (2024); van Eyk et al. (2021)
COA7	Fehlings et al. (2024)	MARS1	Fehlings et al. (2024)	SPG21	Fehlings et al. (2024)
COA8	Fehlings et al. (2024)	MARS2	Fehlings et al. (2024)	SPG7	Fehlings et al. (2024); van Eyk et al. (2021)
COASY	Fehlings et al. (2024)	MARVELD3	van Eyk et al. (2021)	SPP1	Fehlings et al. (2024)
COG2	Fehlings et al. (2024)	MAST1	van Eyk et al. (2023); Fehlings et al. (2024)	SPR	PanelApp Australia; van Eyk et al. (2023); Fehlings et al. (2024)
COG8	Fehlings et al. (2024)	MAT1A	Fehlings et al. (2024)	SPTAN1	PanelApp Australia; Zahrani et al. (2022); van Eyk et al. (2023); Fehlings et al. (2024)
COL12A1	May et al. (2021); Fehlings et al. (2024)	MATN3	van Eyk et al. (2021)	SPTBN2	PanelApp Australia; Rea et al. (2020); van Eyk et al. (2023); Fehlings et al. (2024); van Eyk et al. (2021)
COL18A1	Fehlings et al. (2024)	MATR3	Fehlings et al. (2024)	SQSTM1	Fehlings et al. (2024)
COL1A1	Fehlings et al. (2024)	MBD5	Fehlings et al. (2024)	SRCAP	van Eyk et al. (2021)
COL25A1	Fehlings et al. (2024)	MBOAT7	Fehlings et al. (2024)	SRD5A3	Fehlings et al. (2024)
COL4A1	PanelApp Australia; May et al. (2021); van Eyk et al. (2023); Chopra et al. (2022); Shah et al. (2020); van Eyk et al. (2023); Fehlings et al. (2024); van Eyk et al. (2021)	MBTPS2	Fehlings et al. (2024)	SRGAP1	van Eyk et al. (2021)
COL4A2	PanelApp Australia; van Eyk et al. (2023); van Eyk et al. (2023); Fehlings et al. (2024); van Eyk et al. (2021)	MC2R	Fehlings et al. (2024)	SRPX2	Fehlings et al. (2024)
COL4A4	van Eyk et al. (2021)	MCCC1	Fehlings et al. (2024)	SSR4	Fehlings et al. (2024)
COL5A2	van Eyk et al. (2021)	MCCC2	Fehlings et al. (2024)	ST3GAL5	PanelApp Australia; van Eyk et al. (2023); Fehlings et al. (2024)
COL6A1	Fehlings et al. (2024)	MCOLN1	PanelApp Australia; van Eyk et al. (2023); Fehlings et al. (2024)	STAMBP	PanelApp Australia; van Eyk et al. (2023); Fehlings et al. (2024)

COL6A2	Fehlings et al. (2024)	MCPH1	Fehlings et al. (2024)	STAT1	Fehlings et al. (2024)
COL6A3	Fehlings et al. (2024); van Eyk et al. (2021)	MDH2	Fehlings et al. (2024)	STAT2	Fehlings et al. (2024)
COLEC11	Fehlings et al. (2024)	MDM2	Fehlings et al. (2024)	STAT3	Fehlings et al. (2024)
COLGALT1	Fehlings et al. (2024)	MECP2	PanelApp Australia; May et al. (2021); Zahrani et al. (2022); Kim et al. (2023); van Eyk et al. (2023); Fehlings et al. (2024)	STIL	Fehlings et al. (2024); Fehlings et al. (2024)
COPS3	Fehlings et al. (2024)	MECR	Fehlings et al. (2024)	STK16	Fehlings et al. (2024)
COQ2	Fehlings et al. (2024)	MED12	Fehlings et al. (2024)	STN1	Fehlings et al. (2024)
COQ4	Fehlings et al. (2024)	MED13L	Fehlings et al. (2024)	STRA6	Fehlings et al. (2024)
COQ7	Fehlings et al. (2024)	MED17	Fehlings et al. (2024)	STRADA	van Eyk et al. (2021)
COQ9	Fehlings et al. (2024)	MED25	Fehlings et al. (2024)	STRIP1	van Eyk et al. (2021)
PTGS1	Fehlings et al. (2024)	MEF2C	PanelApp Australia; van Eyk et al. (2023); Fehlings et al. (2024)	STS	Fehlings et al. (2024); van Eyk et al. (2021)
COX10	Fehlings et al. (2024)	MET	Fehlings et al. (2024)	STUB1	Fehlings et al. (2024)
COX15	Fehlings et al. (2024)	MFF	Fehlings et al. (2024)	STX16	Fehlings et al. (2024)
MT-CO2	Fehlings et al. (2024)	MFN2	van Eyk et al. (2023); van Eyk et al. (2023); Fehlings et al. (2024); van Eyk et al. (2021)	STX1B	Fehlings et al. (2024)
CP	Fehlings et al. (2024)	MFSD2A	Fehlings et al. (2024)	STX8	Fehlings et al. (2024)
CPA6	van Eyk et al. (2021)	MFSD8	Fehlings et al. (2024)	STXBP1	PanelApp Australia; van Eyk et al. (2023); Fehlings et al. (2024); van Eyk et al. (2021)
CPLANE1	Fehlings et al. (2024)	MGAT2	Fehlings et al. (2024)	SUCLA2	Fehlings et al. (2024)
CPLX1	Fehlings et al. (2024)	MICOS13	Fehlings et al. (2024)	SUCLG1	Fehlings et al. (2024)
CPS1	Fehlings et al. (2024)	MICU1	Fehlings et al. (2024)	SUMF1	Fehlings et al. (2024)
CPT1A	Fehlings et al. (2024)	MICU2	Fehlings et al. (2024)	SUN1	van Eyk et al. (2021)
CPT1C	ClinGen CP GCEP; Fehlings et al. (2024)	MINPP1	PanelApp Australia; van Eyk et al. (2023); Fehlings et al. (2024)	SUOX	PanelApp Australia; van Eyk et al. (2023); Fehlings et al. (2024)
CPT2	Fehlings et al. (2024)	MITF	van Eyk et al. (2021)	SURF1	Fehlings et al. (2024)
CRADD	Fehlings et al. (2024)	MRTFA	Fehlings et al. (2024)	SUZ12	Fehlings et al. (2024)
CRB1	Fehlings et al. (2024)	MKS1	Fehlings et al. (2024)	SV2C	Fehlings et al. (2024)
CREBBP	PanelApp Australia; van Eyk et al. (2023); Fehlings et al. (2024)	MLC1	van Eyk et al. (2023); Fehlings et al. (2024)	SYNE1	Yechieli et al. (2022); Suchowersky et al. (2021); van Eyk et al. (2023); Fehlings et al. (2024)
CRLF1	Fehlings et al. (2024)	MLEC	Fehlings et al. (2024)	SYNE2	van Eyk et al. (2023); van Eyk et al. (2021)
CRPPA	Fehlings et al. (2024)	MLH3	Fehlings et al. (2024)	SYNGAP1	PanelApp Australia; van Eyk et al. (2023); Fehlings et al. (2024)
CRX	Fehlings et al. (2024)	MLYCD	Fehlings et al. (2024)	SYNJ1	Fehlings et al. (2024)
CSF1R	Fehlings et al. (2024)	MMADHC	Fehlings et al. (2024)	SYP	Fehlings et al. (2024)
CSMD1	PanelApp Australia; May et al. (2021); Fehlings et al. (2024)	MME	Fehlings et al. (2024)	SYT14	Fehlings et al. (2024); van Eyk et al. (2021)
CSNK2A1	Fehlings et al. (2024)	MMUT	Fehlings et al. (2024)	SYT15	Fehlings et al. (2024)
CSPP1	Fehlings et al. (2024)	MOCS1	van Eyk et al. (2023); Fehlings et al. (2024)	SYT2	Fehlings et al. (2024)
CSTB	Matthews et al. (2019); Fehlings et al. (2024)	MOCS2	PanelApp Australia; van Eyk et al. (2023); Fehlings et al. (2024)	SYT9	Fehlings et al. (2024)
CTBP1	van Eyk et al. (2023); Fehlings et al. (2024)	MPC1	Fehlings et al. (2024)	SZT2	Fehlings et al. (2024)

CTC1	Fehlings et al. (2024)	MPDZ	Fehlings et al. (2024)	TAF1	PanelApp Australia; van Eyk et al. (2023); Fehlings et al. (2024); van Eyk et al. (2021)
CTCF	Zahrani et al. (2022); Fehlings et al. (2024)	MPP1	Fehlings et al. (2024)	TAF13	Fehlings et al. (2024)
CTDP1	Fehlings et al. (2024)	MPV17	Fehlings et al. (2024)	TAF15	Fehlings et al. (2024)
CTNNA2	PanelApp Australia; van Eyk et al. (2023)	MPZ	Fehlings et al. (2024)	TAF2	Fehlings et al. (2024)
CTNNB1	PanelApp Australia; Yechieli et al. (2022); Lee et al. (2023); Jin et al. (2020); van Eyk et al. (2023); Fehlings et al. (2024); van Eyk et al. (2021)	MR1	Fehlings et al. (2024)	TANGO2	Zahrani et al. (2022); van Eyk et al. (2023); Fehlings et al. (2024)
CTNND2	Fehlings et al. (2024)	MRPS22	Fehlings et al. (2024)	TARDBP	Fehlings et al. (2024)
CTSD	Fehlings et al. (2024)	MRTFA	Fehlings et al. (2024)	TARS1	Fehlings et al. (2024)
CTSF	Fehlings et al. (2024)	MSL3	PanelApp Australia; Zahrani et al. (2022); van Eyk et al. (2023)	TARS2	Fehlings et al. (2024)
CUL4B	Fehlings et al. (2024)	MST1	Fehlings et al. (2024)	TBC1D17	Fehlings et al. (2024)
CYB5R3	Fehlings et al. (2024)	MSTN	Fehlings et al. (2024)	TBC1D20	Fehlings et al. (2024)
CYP27A1	Fehlings et al. (2024)	MT-TL1	PanelApp Australia; van Eyk et al. (2023); van Eyk et al. (2023); van Eyk et al. (2021)	TBC1D23	Fehlings et al. (2024)
CYP2U1	PanelApp Australia; ClinGen CP GCEP; van Eyk et al. (2023); Fehlings et al. (2024)	MTFMT	Fehlings et al. (2024)	TBC1D24	Fehlings et al. (2024)
CYP7B1	Fehlings et al. (2024)	MTHFR	Fehlings et al. (2024)	TBCD	Fehlings et al. (2024)
D2HGDH	Fehlings et al. (2024)	MTM1	Fehlings et al. (2024)	TBCE	Fehlings et al. (2024)
DAAM1	Fehlings et al. (2024)	MTMR1	Fehlings et al. (2024)	TBCK	Matthews et al. (2019); Fehlings et al. (2024)
DAG1	Fehlings et al. (2024)	MTO1	Fehlings et al. (2024)	TBK1	Fehlings et al. (2024)
DAO	Fehlings et al. (2024)	MTOR	Fehlings et al. (2024)	TBL1XR1	Fehlings et al. (2024)
DARS1	Fehlings et al. (2024)	MTPAP	Fehlings et al. (2024)	TBL2	Fehlings et al. (2024)
DARS2	Fehlings et al. (2024)	MTRFR	Fehlings et al. (2024)	TBX2	van Eyk et al. (2021)
DCAF17	Fehlings et al. (2024)	MYH13	Fehlings et al. (2024)	TCF4	PanelApp Australia; Matthews et al. (2019); van Eyk et al. (2023); Fehlings et al. (2024)
DCHS1	Fehlings et al. (2024)	MYH4	van Eyk et al. (2021)	TCF7L2	Fehlings et al. (2024)
DCN	Fehlings et al. (2024)	MYO15A	Fehlings et al. (2024)	TCOF1	Fehlings et al. (2024)
DCTN1	Fehlings et al. (2024)	MYO5A	Fehlings et al. (2024)	TCTN1	Fehlings et al. (2024)
DCX	Fehlings et al. (2024)	MYO7A	Fehlings et al. (2024)	TCTN2	Fehlings et al. (2024)
DDB2	Fehlings et al. (2024)	MYT1L	Fehlings et al. (2024)	TCTN3	Fehlings et al. (2024)
DDC	PanelApp Australia; van Eyk et al. (2023); Fehlings et al. (2024)	NAA10	PanelApp Australia; Matthews et al. (2019); van Eyk et al. (2023); Fehlings et al. (2024)	TECPR2	Fehlings et al. (2024)
DDHD1	ClinGen CP GCEP; Fehlings et al. (2024)	NAA15	van Eyk et al. (2021)	TELO2	Fehlings et al. (2024)
DDHD2	PanelApp Australia; Yechieli et al. (2022); van Eyk et al. (2023); Fehlings et al. (2024)	NAA35	Fehlings et al. (2024)	TENM1	Fehlings et al. (2024)
DDX3X	PanelApp Australia; Hu et al. (2020); van Eyk et al. (2023); Fehlings et al. (2024)	NAALADL2	Fehlings et al. (2024)	TENM2	van Eyk et al. (2021)
DEAF1	Fehlings et al. (2024); van Eyk et al. (2021)	NACC1	Fehlings et al. (2024)	TENM4	May et al. (2021)
DENND5A	Fehlings et al. (2024)	NADK2	Fehlings et al. (2024)	TEP1	van Eyk et al. (2023)

DEPDC5	Fehlings et al. (2024)	NAGA	Fehlings et al. (2024)	TFG	Fehlings et al. (2024)
ESS2	Fehlings et al. (2024)	NAGLU	Zahrani et al. (2022)	TGFB1	Fehlings et al. (2024)
DGCR2	Fehlings et al. (2024)	NAGPA	Fehlings et al. (2024)	TGFBR2	Fehlings et al. (2024)
DGKZ	Matthews et al. (2019); Fehlings et al. (2024)	NALCN	van Eyk et al. (2023); Fehlings et al. (2024)	TGM6	Fehlings et al. (2024)
DGUOK	Fehlings et al. (2024)	NANS	Fehlings et al. (2024)	TH	PanelApp Australia; van Eyk et al. (2023); Fehlings et al. (2024)
DHCR24	Fehlings et al. (2024)	NARS2	Fehlings et al. (2024)	THAP1	Fehlings et al. (2024)
DHCR7	Fehlings et al. (2024)	NAXE	Fehlings et al. (2024)	THOC2	Chopra et al. (2022)
DHDDS	Fehlings et al. (2024)	NBAS	Fehlings et al. (2024)	THRA	van Eyk et al. (2021)
DHFR	Fehlings et al. (2024)	NBN	Fehlings et al. (2024)	TIMM50	Fehlings et al. (2024)
DHX32	Jin et al. (2020); Fehlings et al. (2024)	NCAPD2	Fehlings et al. (2024)	TIMM8A	Fehlings et al. (2024)
DIP2A	van Eyk et al. (2021)	NCAPG2	Fehlings et al. (2024)	TIMMDC1	Fehlings et al. (2024)
DIP2B	Fehlings et al. (2024)	NCOR1	van Eyk et al. (2021)	TK2	Fehlings et al. (2024)
DIP2C	Fehlings et al. (2024)	NDE1	Fehlings et al. (2024)	TLR7	van Eyk et al. (2021)
DKC1	Fehlings et al. (2024)	NDUFA1	Fehlings et al. (2024)	TMEM126B	Fehlings et al. (2024)
DKK1	Fehlings et al. (2024)	NDUFA10	Fehlings et al. (2024)	TMEM138	Fehlings et al. (2024)
DLAT	Fehlings et al. (2024)	NDUFA11	Fehlings et al. (2024)	TMEM216	Fehlings et al. (2024)
DLD	Fehlings et al. (2024)	NDUFA12	Zahrani et al. (2022); Fehlings et al. (2024)	TMEM231	Fehlings et al. (2024)
DLGAP1	Fehlings et al. (2024)	NDUFA13	Fehlings et al. (2024)	TMEM237	Fehlings et al. (2024)
DLL4	Fehlings et al. (2024)	NDUFA2	Fehlings et al. (2024)	TMEM240	Fehlings et al. (2024)
DMD	Fehlings et al. (2024)	NDUFA9	Fehlings et al. (2024)	TMEM67	Matthews et al. (2019); Fehlings et al. (2024)
DMRT3	Fehlings et al. (2024)	NDUFAF1	Fehlings et al. (2024)	TMEM70	Fehlings et al. (2024)
DMXL1	Fehlings et al. (2024)	NDUFAF2	PanelApp Australia; Zahrani et al. (2022); Fehlings et al. (2024)	TMTC3	Fehlings et al. (2024)
DMXL2	Fehlings et al. (2024)	NDUFAF3	Fehlings et al. (2024)	TMX2	PanelApp Australia; van Eyk et al. (2023)
DNAJC12	Fehlings et al. (2024)	NDUFAF4	Fehlings et al. (2024)	TNF	Fehlings et al. (2024)
DNAJC3	Fehlings et al. (2024)	NDUFAF5	Fehlings et al. (2024)	TNPO3	Fehlings et al. (2024)
DNAJC5	Fehlings et al. (2024)	NDUFAF6	Fehlings et al. (2024)	TNR	PanelApp Australia; van Eyk et al. (2023); Fehlings et al. (2024); van Eyk et al. (2021)
DNAJC6	Fehlings et al. (2024)	NDUFB3	Fehlings et al. (2024)	TOE1	Fehlings et al. (2024)
DNASE1L3	Fehlings et al. (2024)	NDUFB9	Fehlings et al. (2024)	TOPORS	Fehlings et al. (2024)
DNM1	Fehlings et al. (2024)	NDUFS1	Fehlings et al. (2024)	TOR1A	Fehlings et al. (2024)
DNM1L	Fehlings et al. (2024)	NDUFS2	Fehlings et al. (2024)	TOR1AIP1	Fehlings et al. (2024)
DNM2	Fehlings et al. (2024)	NDUFS3	Fehlings et al. (2024)	TP53RK	Fehlings et al. (2024)
DNMT1	Fehlings et al. (2024); van Eyk et al. (2021)	NDUFS4	Fehlings et al. (2024)	TPI1	Fehlings et al. (2024)
DNMT3A	Zahrani et al. (2022); Fehlings et al. (2024)	NDUFS6	Fehlings et al. (2024)	TPK1	Fehlings et al. (2024)
DOCK11	Fehlings et al. (2024)	NDUFS7	Fehlings et al. (2024)	TPP1	Fehlings et al. (2024)
DOCK6	PanelApp Australia; May et al. (2021); van Eyk et al. (2023); Fehlings et al. (2024); van Eyk et al. (2021)	NDUFS8	Fehlings et al. (2024)	TPRKB	Fehlings et al. (2024)
DOCK7	Fehlings et al. (2024)	NDUFV1	Fehlings et al. (2024)	TRAIP	Fehlings et al. (2024)
DOCK8	van Eyk et al. (2021)	NDUFV2	Fehlings et al. (2024)	TRAK1	Fehlings et al. (2024)

DPM3	Fehlings et al. (2024)	NECAP1	Fehlings et al. (2024); van Eyk et al. (2021)	TRAPPC11	Fehlings et al. (2024)
DPP6	Fehlings et al. (2024)	NECTIN2	van Eyk et al. (2021)	TRAPPC12	Fehlings et al. (2024)
DPYS	Fehlings et al. (2024)	NEDD4L	Fehlings et al. (2024)	TRAPPC9	PanelApp Australia; van Eyk et al. (2023); Fehlings et al. (2024)
DRD1	Fehlings et al. (2024)	NEFH	Fehlings et al. (2024)	TREM2	Fehlings et al. (2024)
DRD2	Fehlings et al. (2024)	NEK1	Fehlings et al. (2024)	TREX1	PanelApp Australia; van Eyk et al. (2023); Fehlings et al. (2024)
DRD3	Fehlings et al. (2024)	NELFA	Fehlings et al. (2024)	TRIM32	Fehlings et al. (2024); van Eyk et al. (2021)
DST	van Eyk et al. (2021)	NEXMIF	Fehlings et al. (2024)	TRIM8	Fehlings et al. (2024)
DSTYK	ClinGen CP GCEP; Fehlings et al. (2024)	NF1	van Eyk et al. (2023); Fehlings et al. (2024); van Eyk et al. (2021)	TRIO	Fehlings et al. (2024); van Eyk et al. (2021)
DYNC1H1	PanelApp Australia; van Eyk et al. (2023); Fehlings et al. (2024)	NFIX	van Eyk et al. (2023); Fehlings et al. (2024)	TRIP12	Fehlings et al. (2024)
DYRK1A	PanelApp Australia; van Eyk et al. (2023); Fehlings et al. (2024)	NFU1	Fehlings et al. (2024)	TRIT1	Fehlings et al. (2024)
EARS2	PanelApp Australia; Zahrani et al. (2022); van Eyk et al. (2023); Fehlings et al. (2024)	NGLY1	PanelApp Australia; van Eyk et al. (2023); Fehlings et al. (2024)	TRMT5	Fehlings et al. (2024)
EBF1	Fehlings et al. (2024)	NHLRC1	Fehlings et al. (2024)	TRPV4	Fehlings et al. (2024)
EBF3	Fehlings et al. (2024)	NIN	Fehlings et al. (2024)	TSC1	van Eyk et al. (2023); Fehlings et al. (2024)
ECHS1	PanelApp Australia; Zahrani et al. (2022); Chopra et al. (2022); van Eyk et al. (2023); Fehlings et al. (2024)	NIPA1	Fehlings et al. (2024); van Eyk et al. (2021)	TSC2	Fehlings et al. (2024)
ECM1	Fehlings et al. (2024)	NIPBL	Fehlings et al. (2024)	TSEN15	Fehlings et al. (2024)
ECSIT	Fehlings et al. (2024)	NKAIN2	Fehlings et al. (2024)	TSEN2	Fehlings et al. (2024)
EDNRB	Fehlings et al. (2024)	NKX2-1	PanelApp Australia; van Eyk et al. (2023); Fehlings et al. (2024)	TSEN34	Fehlings et al. (2024)
EED	Fehlings et al. (2024)	NKX2-5	Fehlings et al. (2024)	TSEN54	Fehlings et al. (2024)
EEF1A2	van Eyk et al. (2023); Fehlings et al. (2024)	NKX2-6	van Eyk et al. (2021)	TSFM	Fehlings et al. (2024)
EEF2	Fehlings et al. (2024)	NKX6-2	Fehlings et al. (2024); Fehlings et al. (2024)	TSHB	Fehlings et al. (2024)
EEFSEC	Fehlings et al. (2024)	NMNAT1	Fehlings et al. (2024)	TSHR	Fehlings et al. (2024)
EFEMP1	van Eyk et al. (2021)	NOS1	Fehlings et al. (2024)	TTBK2	Fehlings et al. (2024)
EFTUD2	Fehlings et al. (2024)	NOTCH1	Fehlings et al. (2024); van Eyk et al. (2021)	TTC19	Fehlings et al. (2024)
EGFR	van Eyk et al. (2021)	NOTCH3	Fehlings et al. (2024); van Eyk et al. (2021)	TTC21B	Fehlings et al. (2024)
EHMT1	Matthews et al. (2019); Fehlings et al. (2024)	NPC1	Ko et al. (2019); Fehlings et al. (2024)	TTF1	Fehlings et al. (2024)
EIF2AK1	Fehlings et al. (2024)	NPC2	Fehlings et al. (2024)	TTN	van Eyk et al. (2023); Fehlings et al. (2024); van Eyk et al. (2021)
EIF2AK2	Fehlings et al. (2024)	NPHP1	Fehlings et al. (2024)	TTPA	Fehlings et al. (2024)
EIF2AK3	Fehlings et al. (2024)	NPHP3	Fehlings et al. (2024)	TTR	Fehlings et al. (2024)
EIF2B1	Fehlings et al. (2024)	NPRL3	Fehlings et al. (2024)	TUBA1A	PanelApp Australia; van Eyk et al. (2023); Yechieli et al. (2022); Jin et al. (2020); van Eyk et al. (2023); Fehlings et al. (2024); van Eyk et al. (2021)
EIF2B2	Fehlings et al. (2024)	NR2F1	Fehlings et al. (2024)	TUBA8	Fehlings et al. (2024)
EIF2B3	Fehlings et al. (2024)	NR4A2	Fehlings et al. (2024)	TUBB	Fehlings et al. (2024)

EIF2B4	Fehlings et al. (2024)	NRDC	Fehlings et al. (2024)	TUBB2A	PanelApp Australia; ClinGen CP GCEP; van Eyk et al. (2023); Fehlings et al. (2024)
EIF2B5	Fehlings et al. (2024)	NRG3	Fehlings et al. (2024)	TUBB2B	PanelApp Australia; van Eyk et al. (2023); Fehlings et al. (2024)
EIF2S3	Fehlings et al. (2024)	NRXN1	Fehlings et al. (2024)	TUBB3	PanelApp Australia; van Eyk et al. (2023); Fehlings et al. (2024)
ELAC2	Fehlings et al. (2024)	NSD1	Fehlings et al. (2024)	TUBB4A	PanelApp Australia; van Eyk et al. (2023); Matthews et al. (2019); van Eyk et al. (2023); Fehlings et al. (2024); van Eyk et al. (2021)
ELN	Fehlings et al. (2024)	NSDHL	Fehlings et al. (2024)	TUBG1	Fehlings et al. (2024)
ELOVL1	PanelApp Australia; Yechieli et al. (2022); van Eyk et al. (2023)	NSRP1	PanelApp Australia; van Eyk et al. (2023)	TUBGCP4	Fehlings et al. (2024)
ELOVL4	Fehlings et al. (2024); van Eyk et al. (2021)	NSUN2	Fehlings et al. (2024)	TUBGCP6	Fehlings et al. (2024)
ELOVL5	Fehlings et al. (2024)	NT5C2	PanelApp Australia; ClinGen CP GCEP; van Eyk et al. (2023); Fehlings et al. (2024)	TUFM	Fehlings et al. (2024)
ELP2	PanelApp Australia; van Eyk et al. (2023); Fehlings et al. (2024)	NTNG1	Fehlings et al. (2024)	TULP1	Fehlings et al. (2024)
ELP4	Fehlings et al. (2024)	NTRK2	Fehlings et al. (2024)	TUSC3	Fehlings et al. (2024)
EMC1	ClinGen CP GCEP; Fehlings et al. (2024); van Eyk et al. (2021)	NUBPL	Fehlings et al. (2024)	TWIST1	Fehlings et al. (2024)
EML1	Fehlings et al. (2024)	NUP107	Fehlings et al. (2024)	TWNK	Fehlings et al. (2024)
EML2	van Eyk et al. (2021)	NUP62	Fehlings et al. (2024)	TXN2	Fehlings et al. (2024)
ENTPD1	ClinGen CP GCEP; Fehlings et al. (2024)	NUS1	Fehlings et al. (2024)	TYMP	Fehlings et al. (2024)
EOGT	Fehlings et al. (2024)	OCLN	Fehlings et al. (2024)	TYROBP	Fehlings et al. (2024)
EP300	Fehlings et al. (2024)	OCRL	Fehlings et al. (2024)	TYW1	Fehlings et al. (2024)
EP400	Fehlings et al. (2024)	OFD1	Fehlings et al. (2024)	UBA1	Fehlings et al. (2024)
EPB41L4A	Fehlings et al. (2024)	OGDHL	Fehlings et al. (2024)	UBA5	Fehlings et al. (2024)
EPCAM	Fehlings et al. (2024)	OLIG2	Fehlings et al. (2024)	UBB	Fehlings et al. (2024)
EPG5	Fehlings et al. (2024)	OPA1	Fehlings et al. (2024)	UBE3A	PanelApp Australia; van Eyk et al. (2023); Fehlings et al. (2024)
EPHA4	Matthews et al. (2019); Fehlings et al. (2024)	OPA3	Fehlings et al. (2024)	UBQLN2	Fehlings et al. (2024); van Eyk et al. (2021)
EPHA6	Fehlings et al. (2024)	OPHN1	Fehlings et al. (2024)	UCHL1	Fehlings et al. (2024)
EPM2A	Fehlings et al. (2024)	OPTN	Fehlings et al. (2024)	UNC13A	Fehlings et al. (2024)
ERBB4	Fehlings et al. (2024)	OSGEP	Fehlings et al. (2024)	UNC80	Fehlings et al. (2024); van Eyk et al. (2021)
ERCC1	Fehlings et al. (2024)	OTUD6B	Fehlings et al. (2024)	UPB1	Fehlings et al. (2024)
ERCC2	Fehlings et al. (2024)	PACRG	Fehlings et al. (2024)	UPF3B	Fehlings et al. (2024); Fehlings et al. (2024)
ERCC3	Fehlings et al. (2024)	PACS1	Fehlings et al. (2024)	UQCRQ	Fehlings et al. (2024)
ERCC4	Fehlings et al. (2024)	PAFAH1B1	PanelApp Australia; van Eyk et al. (2023); Fehlings et al. (2024)	UROC1	Fehlings et al. (2024)
ERCC5	Fehlings et al. (2024)	PAH	Fehlings et al. (2024)	USP18	Fehlings et al. (2024)
ERCC6	Fehlings et al. (2024)	PAK3	PanelApp Australia; Matthews et al. (2019); van Eyk et al. (2023); Fehlings et al. (2024)	USP32	van Eyk et al. (2021)

ERCC8	PanelApp Australia; van Eyk et al. (2023); Fehlings et al. (2024)	PALM	Matthews et al. (2019); Fehlings et al. (2024)	USP41P	van Eyk et al. (2021)
ERLIN1	Fehlings et al. (2024)	PANK2	PanelApp Australia; May et al. (2021); van Eyk et al. (2023); Fehlings et al. (2024)	USP7	Fehlings et al. (2024)
ERLIN2	Fehlings et al. (2024)	PRKN	Fehlings et al. (2024); van Eyk et al. (2021)	USP8	Fehlings et al. (2024)
ERMARD	Fehlings et al. (2024)	PARK7	Fehlings et al. (2024)	USP9X	Fehlings et al. (2024)
ESAM	PanelApp Australia	PARS2	Fehlings et al. (2024)	VAC14	Fehlings et al. (2024)
ESYT2	Fehlings et al. (2024)	PAX3	Fehlings et al. (2024)	VAMP1	Fehlings et al. (2024); van Eyk et al. (2021)
ETFA	Fehlings et al. (2024)	PAX6	Fehlings et al. (2024)	VAPB	Fehlings et al. (2024)
ETFB	Fehlings et al. (2024)	PAX8	Fehlings et al. (2024)	VARS2	Fehlings et al. (2024)
ETFDH	Fehlings et al. (2024)	PC	Fehlings et al. (2024)	VCP	Fehlings et al. (2024)
ETHE1	Fehlings et al. (2024)	PCBP3	Fehlings et al. (2024); van Eyk et al. (2021)	VCX3A	van Eyk et al. (2021)
EXD1	van Eyk et al. (2021)	PCCA	Fehlings et al. (2024)	VIPAS39	Fehlings et al. (2024)
EXOSC3	PanelApp Australia; van Eyk et al. (2023); Fehlings et al. (2024)	PCCB	Fehlings et al. (2024)	VLDLR	Fehlings et al. (2024)
EXOSC8	Fehlings et al. (2024)	PCDH12	PanelApp Australia; Yechieli et al. (2022); van Eyk et al. (2023); Fehlings et al. (2024)	VPS11	Yechieli et al. (2022); Fehlings et al. (2024)
EXOSC9	Fehlings et al. (2024)	PCDH19	Zahrani et al. (2022); Yechieli et al. (2022); Fehlings et al. (2024)	VPS13A	Fehlings et al. (2024)
EZH2	Fehlings et al. (2024)	PCLO	Fehlings et al. (2024)	VPS13C	Fehlings et al. (2024)
F2	van Eyk et al. (2021)	PCYT1A	Fehlings et al. (2024)	VPS13D	PanelApp Australia; Fehlings et al. (2024)
F8	van Eyk et al. (2021)	PCYT2	PanelApp Australia; Fehlings et al. (2024)	VPS16	Fehlings et al. (2024)
FA2H	Fehlings et al. (2024)	PDC	Fehlings et al. (2024)	VPS33B	Fehlings et al. (2024)
FAAH	Fehlings et al. (2024)	PDCD6IP	Fehlings et al. (2024)	VPS37A	Fehlings et al. (2024)
FADD	Fehlings et al. (2024)	PDE10A	Fehlings et al. (2024)	VPS50	Fehlings et al. (2024)
HYCC1	van Eyk et al. (2023)	PDE2A	Fehlings et al. (2024)	VPS51	Fehlings et al. (2024)
FAN1	Fehlings et al. (2024)	PDGFA	van Eyk et al. (2021)	VPS53	Fehlings et al. (2024); van Eyk et al. (2021)
FAR1	PanelApp Australia; van Eyk et al. (2023); Fehlings et al. (2024)	PDGFB	Fehlings et al. (2024)	VRK1	Fehlings et al. (2024)
FARS2	PanelApp Australia; van Eyk et al. (2023); Fehlings et al. (2024); van Eyk et al. (2021)	PDGFRB	Fehlings et al. (2024); van Eyk et al. (2021)	VWA3B	Fehlings et al. (2024)
FASN	Fehlings et al. (2024)	PDHA1	PanelApp Australia; van Eyk et al. (2023); Fehlings et al. (2024)	VWF	van Eyk et al. (2021)
FAT4	Fehlings et al. (2024)	PDHB	Fehlings et al. (2024)	WAC	Fehlings et al. (2024)
FBN1	van Eyk et al. (2021)	PDHX	PanelApp Australia; van Eyk et al. (2023); Fehlings et al. (2024)	WARS2	Zahrani et al. (2022); Fehlings et al. (2024)
FBN2	Fehlings et al. (2024)	PDP1	Fehlings et al. (2024)	WASHC4	Fehlings et al. (2024)
FBXL4	Fehlings et al. (2024)	PDX1	Fehlings et al. (2024)	WASHC5	Fehlings et al. (2024)
FBXO31	PanelApp Australia; Jin et al. (2020); van Eyk et al. (2023); Fehlings et al. (2024)	PDYN	Fehlings et al. (2024)	WDR26	PanelApp Australia; van Eyk et al. (2023); Fehlings et al. (2024)
FBXO32	Fehlings et al. (2024)	PEX1	Fehlings et al. (2024); van Eyk et al. (2021)	WDR45	PanelApp Australia; Zahrani et al. (2022); Matthews et al. (2019); van Eyk et al. (2023); Fehlings et al. (2024)

FBXO7	Fehlings et al. (2024)	PEX10	Fehlings et al. (2024)	WDR47	van Eyk et al. (2021)
FERMT3	van Eyk et al. (2021)	PEX11B	Fehlings et al. (2024)	WDR48	Fehlings et al. (2024)
FGD1	Fehlings et al. (2024); Fehlings et al. (2024)	PEX12	Fehlings et al. (2024)	WDR62	Fehlings et al. (2024)
FGF12	Fehlings et al. (2024)	PEX13	Fehlings et al. (2024)	WDR73	Fehlings et al. (2024)
FGF14	Fehlings et al. (2024)	PEX14	Fehlings et al. (2024); van Eyk et al. (2021)	WDR74	Fehlings et al. (2024)
FGFR1	Fehlings et al. (2024); van Eyk et al. (2021)	PEX16	Fehlings et al. (2024); Fehlings et al. (2024)	WDR81	Fehlings et al. (2024)
FGFR2	Fehlings et al. (2024); van Eyk et al. (2021)	PEX19	Fehlings et al. (2024)	NSD2	Fehlings et al. (2024)
FGFR3	Fehlings et al. (2024)	PEX2	Fehlings et al. (2024)	WIPI2	Fehlings et al. (2024)
FGFRL1	Fehlings et al. (2024)	PEX26	Fehlings et al. (2024)	WNK3	van Eyk et al. (2021)
FH	PanelApp Australia; van Eyk et al. (2023); Fehlings et al. (2024)	PEX3	Fehlings et al. (2024)	WNK4	van Eyk et al. (2021)
FHL1	Fehlings et al. (2024)	PEX5	Fehlings et al. (2024)	WNT4	Fehlings et al. (2024)
FIG4	Fehlings et al. (2024)	PEX6	Fehlings et al. (2024)	WNT8B	Fehlings et al. (2024)
FIGLA	Fehlings et al. (2024)	PEX7	Fehlings et al. (2024)	WWOX	PanelApp Australia; van Eyk et al. (2023); Fehlings et al. (2024); van Eyk et al. (2021)
FIGN	van Eyk et al. (2021)	PFN1	Fehlings et al. (2024)	XIAP	Fehlings et al. (2024)
FITM2	ClinGen CP GCEP; Fehlings et al. (2024)	PGAP1	Fehlings et al. (2024)	XPA	Fehlings et al. (2024)
FKRP	Fehlings et al. (2024)	PGAP2	Fehlings et al. (2024)	XPC	Fehlings et al. (2024)
FKTN	Fehlings et al. (2024)	PGK1	Rosello et al. (2021)	XPR1	Fehlings et al. (2024)
FLI1	Fehlings et al. (2024)	PHC1	Fehlings et al. (2024)	XRCC4	Fehlings et al. (2024)
FLNA	van Eyk et al. (2023); Fehlings et al. (2024)	PHF6	Fehlings et al. (2024)	YME1L1	Fehlings et al. (2024)
FLRT1	Fehlings et al. (2024)	PHF8	Yechieli et al. (2022)	YWHAE	Fehlings et al. (2024)
FOLR1	Fehlings et al. (2024)	PHGDH	Fehlings et al. (2024)	YWHAG	Fehlings et al. (2024)
FOXC1	Fehlings et al. (2024)	PHIP	PanelApp Australia; Fehlings et al. (2024)	YY1	Fehlings et al. (2024)
FOXE1	Fehlings et al. (2024)	PHKA1	Fehlings et al. (2024)	ZBTB18	Fehlings et al. (2024)
FOXG1	PanelApp Australia; van Eyk et al. (2023); Fehlings et al. (2024)	PHYH	Fehlings et al. (2024)	ZBTB20	Fehlings et al. (2024)
FOXP1	Fehlings et al. (2024); van Eyk et al. (2021)	PIEZO2	Fehlings et al. (2024); van Eyk et al. (2021)	ZC4H2	PanelApp Australia; van Eyk et al. (2023); Fehlings et al. (2024)
FOXRED1	Fehlings et al. (2024)	PIGA	PanelApp Australia; van Eyk et al. (2023); Fehlings et al. (2024)	ZDHHC15	Fehlings et al. (2024)
FRAS1	Fehlings et al. (2024)	PIGG	Fehlings et al. (2024)	ZDHHC9	Fehlings et al. (2024)
FREM2	Fehlings et al. (2024)	PIGN	PanelApp Australia; van Eyk et al. (2023); Fehlings et al. (2024)	ZEB2	PanelApp Australia; van Eyk et al. (2023); Fehlings et al. (2024)
FRRS1L	van Eyk et al. (2023); Fehlings et al. (2024)	PIGO	Fehlings et al. (2024)	ZFHX3	May et al. (2021); Fehlings et al. (2024); van Eyk et al. (2021)
FSCB	Fehlings et al. (2024)	PIGP	Fehlings et al. (2024)	ZFR	Fehlings et al. (2024)
FTHL17	Fehlings et al. (2024)	PIGT	Fehlings et al. (2024)	ZFYVE26	Fehlings et al. (2024); van Eyk et al. (2021)
FTL	Fehlings et al. (2024)	PIGV	Fehlings et al. (2024)	ZFYVE27	Fehlings et al. (2024)
FTO	Fehlings et al. (2024)	DNAAF6	van Eyk et al. (2021)	ZIC1	Fehlings et al. (2024)
FUCA1	Fehlings et al. (2024)	PIK3CA	Fehlings et al. (2024)	ZMYM2	Chopra et al. (2022)
FUS	Fehlings et al. (2024)	PIK3CD	Fehlings et al. (2024)	ZNF148	Fehlings et al. (2024)

FXN	Fehlings et al. (2024)	PIK3R2	Fehlings et al. (2024)	ZNF335	Fehlings et al. (2024)
G6PD	Fehlings et al. (2024)	PINK1	Fehlings et al. (2024)	ZNF423	Fehlings et al. (2024)
GABBR2	Fehlings et al. (2024)	PLA2G6	PanelApp Australia; van Eyk et al. (2023); Fehlings et al. (2024)	ZNF592	Fehlings et al. (2024)
GABRA1	Fehlings et al. (2024)	PLAA	Fehlings et al. (2024)	ZNF74	van Eyk et al. (2021)
GABRA2	Fehlings et al. (2024)	PLCB1	Fehlings et al. (2024)	ZNF778	Fehlings et al. (2024)
GABRB1	van Eyk et al. (2023); Fehlings et al. (2024)	PLEKHG2	Fehlings et al. (2024)	ZSWIM6	PanelApp Australia; May et al. (2021); van Eyk et al. (2023); Fehlings et al. (2024)

HGNC symbol and source for genes comprising the master CP gene list. This list was compiled from cerebral palsy gene lists available in the following databases and publications: 161 genes from the diagnostic grade “Green” genes from the Cerebral Palsy panel (Version 1.389), PanelApp Australia; 17 genes with a clinical validity classification of Definitive or Moderate from the ClinGen Cerebral Palsy Gene Curation Expert Panel (https://clinicalgenome.org/affiliation/40113/) ; 141 genes from Basu et al. (2024); 168 genes from van Eyk et al. (2021) and 208 genes from van Eyk et al. (2023); 1411 genes from Fehlings et al. (2024). Gene lists were combined, duplicated genes were removed and the remaining gene symbols checked against approved symbols using the Multi-symbol checker tool from HUGO Gene Nomenclature Committee (https://www.genenames.org/tools/multi-symbol-checker/).

Supplementary Table 3: Red flag features

Category	Red flags	HPO Term	HPO	Exclude term
Clinical presentation	Developmental regression	Developmental regression	HP:0002376	
Clinical presentation	Progressive neurologic deterioration	Progressive neurologic deterioration	HP:0002344	
Clinical presentation	Movement disorder	Ataxia	HP:0001251	
Clinical presentation	Movement disorder	Dyskinesia	HP:0100660	
Clinical presentation	Movement disorder	Dystonia	HP:0001332	
Clinical presentation	Movement disorder	Athetosis	HP:0002305	
Clinical presentation	Global developmental delay	Global developmental delay	HP:0001263	
Clinical presentation	Global developmental delay	Intellectual disability	HP:0001249	
Clinical presentation	Epilepsy	Seizure	HP:0001250	
Clinical presentation	Behavioural concerns	Attention deficit hyperactivity disorder	HP:0007018	
Clinical presentation	Behavioural concerns	Anxiety	HP:0000739	

Clinical presentation	Behavioural concerns	Atypical behavior	HP:0000708	
Clinical presentation	Other systems	Abnormal heart morphology	HP:0001627	
Clinical presentation	Other systems	Retinopathy	HP:0000488	
Clinical presentation	Other systems	Abnormality of the extraocular muscles	HP:0008049	
Clinical presentation	Other systems	Limb muscle weakness	HP:0003690	
Perinatal history	Intrauterine growth retardation	Intrauterine growth retardation	HP:0001511	
Perinatal history	Multiple miscarriages	Miscarriage	HP:0005268	
Perinatal history	Anomaly scan abnormality	Fetal anomaly	HP:0034057	
Perinatal history	Postnatal failure to thrive	Failure to thrive	HP:0001508	
Perinatal history	Postnatal failure to thrive	Feeding difficulties	HP:0011968	
Additional investigations	Corpus callosum abnormalities	Abnormal corpus callosum morphology	HP:0001273	
Additional investigations	Cerebellar atrophy	Cerebellar atrophy	HP:0001272	
Additional investigations	Basal ganglia abnormalities	Abnormal basal ganglia morphology	HP:0002134	
Additional investigations	White matter changes or other neurodegenerative changes	Neurodegeneration	HP:0002180	
Additional investigations	White matter changes or other neurodegenerative changes	Multifocal hyperintensity of cerebral white matter on MRI	HP:0040329	
Additional investigations	White matter changes or other neurodegenerative changes	Focal hyperintensity of cerebral white matter on MRI	HP:0040328	
Additional investigations	White matter changes or other neurodegenerative changes	Deep cerebral white matter hyperintensities	HP:0030892	
Additional investigations	White matter changes or other neurodegenerative changes	Confluent hyperintensity of cerebral white matter on MRI	HP:0040330	
Additional investigations	White matter changes or other neurodegenerative changes	Atrophy/Degeneration affecting the cerebrum	HP:0007369	
Additional investigations	Abnormal myelination	Abnormal CNS myelination	HP:0011400	
Additional investigations	Abnormal cerebral morphology	Abnormal cerebral morphology	HP:0002060	Encephalomalacia
Additional investigations	Abnormal cerebellum morphology	Abnormal cerebellum morphology	HP:0001317	
Additional investigations	Normal MRI brain			

Targeted investigations	CSF	Abnormal CSF metabolite concentration	HP:0025454	
Targeted investigations	CSF	Abnormal CSF protein concentration	HP:0025456	
Targeted investigations	Metabolic	Abnormal circulating metabolite concentration	HP:0032180	
Targeted investigations	Metabolic	Abnormal urine metabolite level	HP:0033354	
Targeted investigations	Metabolic	Abnormal tissue metabolite concentration	HP:0032243	
Targeted investigations	Metabolic	Abnormality of mitochondrial metabolism	HP:0003287	
Targeted investigations	Metabolic	Abnormal mitochondrial morphology	HP:0008322	
Targeted investigations	Muscle	Abnormal muscle fiber morphology	HP:0004303	
Targeted investigations	Muscle	Myopathy	HP:0003198	
Targeted investigations	Muscle	EMG abnormality	HP:0003457	
Examination	Syndromic features	Microcephaly	HP:0000252	
Examination	Syndromic features	Abnormal facial shape	HP:0001999	
Examination	Syndromic features	Abnormal skeletal morphology	HP:0011842	
Examination	Abnormal gait	Gait ataxia	HP:0002066	
Examination	Abnormal gait	Dystonic gait	HP:0031954	
Examination	Abnormal gait	Unsteady gait	HP:0002317	
Examination	Abnormal gait	Shuffling gait	HP:0002362	
Examination	Abnormal gait	Waddling gait	HP:0002515	
Examination	Abnormal gait	Tip-toe gait	HP:0030051	
Examination	Abnormal gait	Impaired tandem gait	HP:0031629	
Examination	Abnormal gait	Gait imbalance	HP:0002141	
Examination	Abnormal gait	Loss of ambulation	HP:0002505	
Examination	Abnormal gait	Cerebellar ataxia associated with quadrupedal gait	HP:0009878	

Examination	Abnormal gait	Broad-based gait	HP:0002136	
Examination	Abnormal movement	Involuntary movements	HP:0004305	
Examination	Abnormal movement	Hyperkinetic movements	HP:0002487	
Examination	Abnormal movement	Muscle fibrillation	HP:0010546	
Examination	Abnormal movement	Diminished movement	HP:0002374	
Examination	Abnormal movement	Abnormal head movements	HP:0002457	
Examination	Abnormal movement	Abnormal posturing	HP:0002533	
Examination	Scoliosis	Scoliosis	HP:0002650	
Examination	Hypotonia	Hypotonia	HP:0001252	
Family history	Family history present	Family history	HP:0032316	

Supplementary Table 4: Cases with a clinically confirmed and reported P/LP variant

Case	Family_size	Gene	Type	VEP_Consequence	Zygosity	Inheritance (variant)	HGVSC / ISCN	HGVSP	Condition	P/LP	ACMG codes applied
NNET1005	Duo (affected sibling)	*PMP22*	DUP	N/A	Unknown	Unknown	NC_[000017.11]: g.14159934_15527543dup	N/A	Hereditary Motor and Sensory Neuropathy Type 1A/Charcot Marie-Tooth Type 1A	P	N/A
NNET1005	Duo (affected sibling)	*EPHA2*	SNV	splice_polypyrimidine_tract_and_intron	Het	Unknown	NM_004431.5: c.2826-9G>A	N/A	*EPHA2*-related cataract	P	PS3_Strong; PM2_Moderate; PP1_Strong
NNET1017	Trio	*MT-ATP6*	SNV	missense_variant	N/A	Maternal	ENST00000361899.2: c.467T>G	p.(Leu156Arg)	*MT-ATP6*-related mitochondrial disease	P	PS4_Strong; PM2_Moderate; PM5_Moderate; PM6_Moderate; PP1_Moderate; PP3_Supporting
NNET1019	Trio	*F11*	SNV	missense_variant	Het	Maternal	NM_000128.4: c.901T>C	NP_000119.1 :p.(Phe301Leu)	Factor XI deficiency	P	PS3_Supporting; PM2_Moderate; PM3_VeryStrong; PP3_Supporting
NNET1020	Trio	*CIC*	SNV	stop-gained	Het	Paternal	NM_015125.5: c.3640C>T	NP_055940.3: p.(Arg1214Ter)	*CIC*-related disorder	P	PVS1_VeryStrong; PM2_Moderate
NNET2019	Trio	*SCN1A*	SNV	missense_variant	Het	de novo	NM_001165963.4: c.5494G>C	NP_001159435.1: p.(Ala1832Pro)	*SCN1A*-related disorder	LP	PS2_Moderate; PS4_Supporting; PM2_Moderate; PP2_Supporting; PP3_Supporting
NNET2020	Duo	*KRIT1*	INDEL	frameshift_variant	Het	Unknown	NM_194456.1: c.397dup	NP_919438.1: p.(Tyr133LeufsTer11)	Cerebral cavernous malformations-1	P	PVS1_VeryStrong; PM2_Moderate

NNET3008	Trio	*SETD1A*	SNV	stop-gained	Het	Paternal	NM_014712.3: c.2944G>T	NP_055527.1: p.(Glu982Ter)	*SETD1A*-related disorder	P	PVS1_Very Strong; PM2_Moderate
NNET3023	Duo	*TUBB2B*	SNV	missense_variant	Het	Maternal	NM_178012.5: c.1034T>A	NP_821080.1: p.(Ile345Asn)	*TUBB2B*-related disorder	LP	PM2_Moderate; PP2_Supporting; PP3_Supporting; PP4_Moderate
NNET3025	Trio	*ATL1*	SNV	missense_variant	Het	Paternal	NM_015915.5: c.1246C>T	NP_056999.2: p.(Arg416Cys)	*ATL1*-related spastic paraplegia	P	PS4_Strong; PP1_Strong; PM1_Moderate
NNET4012	Trio	*COL4A1*	SNV	missense_variant	Het	Maternal	NM_001845.6: c.3958G>A	NP_001836.3: p.(Gly1320Ser)	*COL4A1*-related disorder	LP	PM1_Strong; PM2_Moderate; PP3_Supporting
NNET4013	Trio	*TREX1*	SNV	stop-gained	Het	Paternal	NM_033629.6: c.490C>T	NP_338599.1: p.(Arg164Ter)	Aicardi-Goutières syndrome 1	P	PVS1_VeryStrong; PM2_Moderate; PM3_Moderate
NNET4013	Trio	*TREX1*	INDEL	frameshift_variant	Het	Maternal	NM_033629.6: c.764_765dup	NP_338599.1: p.(Thr256ProfsTer22)	Aicardi-Goutières syndrome 1	LP	PVS1_Strong; PM2_Moderate; PM3_Moderate

SNV = single nucleotide variant; INDEL = insertion / deletion; DUP = duplication. Het = heterozygous; P = pathogenic; LP = likely pathogenic.

Supplementary Table 5: Cases with a variant of interest

Case	Family_size	Gene	Type	VEP_Consequence	Zygosity	Inheritance (variant)	HGVSC / ISCN	HGVSP	Condition	P/LP	ACMG codes applied
NNET1006	Trio	*ADAR*	SNV	missense_variant	Het	Maternal	NM_001111.5: c.577C>G	NP_001102.3 p.(Pro193Ala)	Aicardi-Goutières syndrome	LP	PS3_Supporting; PM2_Moderate; PM3_Strong; PP2_Supporting; PP3_Supporting
NNET1010	Trio	*VWF*	SNV	missense_variant	Het	Maternal	NM_000552.5: c.2561G>A	NP_000543.3 p.(Arg854Gln)	von Willebrand disease type 2N	P	PS3_Supporting, PM3_Strong, PP4_Moderate, PP1_Supporting
NNET2010	Duo	*NIPA1;NIPA2*	DEL	N/A	Unknown	Unknown	NC_000015.9: g.22648948_23124083del	N/A	15q11.2 microdeletion	P	N/A
NNET2015	Trio	*F2*	SNV	3_prime_UTR_variant	Het	Maternal	NM_000506.5: c.*97G>A	N/A	Cerebral infarction, susceptibility to	P	PS4_VeryStrong; PM2_Moderate; PM3_Moderate
NNET3004	Trio	*POLG*	SNV	missense_variant	Het	Paternal	NM_002693.3: c.2209G>C	NP_002684.1: p.(Gly737Arg)	Primary mitochondrial disease	P	PS4_Moderate; PM3_Strong; PP3_Supporting
NNET3024	Trio	*PAX8*	INDEL	frameshift_variant	Het	Maternal	NM_003466.4: c.936dup	NP_003457.1: p.(Glu313ArgfsTer6)	Hypothyroidism, congenital, due to thyroid dysgenesis or hypoplasia	LP	PVS1_Strong; PM2_Moderate; PM3_Moderate
NNET4003	Trio	*ARFGEF1*	SNV	missense_variant	Het	Maternal	NM_006421.5: c.1441A>T	NP_006412.2 p.Ile481Phe	Developmental delay, impaired speech, and behavioral abnormalities, with or without seizures	VUS	PM2_Moderate; PP2_Supporting
NNET4006	Trio	*COL11A1*	SNV	splice_donor_variant	Het	Maternal	NM_001854.4:c.1245+1G>A	N/A	Stickler syndrome	LP	PVS1_Strong; PS4_Supporting; PP3_Supporting

SNV = single nucleotide variant; INDEL = insertion / deletion; DEL = deletion. Het = heterozygous; P = pathogenic; LP = likely pathogenic; VUS = variant of uncertain significance.

Supplementary Table 6: Cases with VUS in a CP gene

Case	Family size	Gene	Type	VEP Consequence	Zygosity	Inheritance (variant)	HGVSC	HGVSP	Condition	ACMG codes applied	Points	VUS sub-classification
NNET1009	Trio	*ZMYM2*	SNV	missense_variant	Het	Paternal	NM_003453.6: c.628A>G	NP_003444.1: p.(Met210Val)	Neurodevelopmental-craniofacial syndrome with variable renal and cardiac abnormalities	PM2_Moderate, BP4_Supporting	1	Low
NNET1016	Trio	*IFIH1*	SNV	missense_variant	Het	Maternal	NM_022168.4: c.1092T>G	NP_071451.2: p.(Asn364Lys)	Aicardi-Goutières syndrome	PM2_Moderate; BP4_Supporting	1	Low
NNET1018	Trio	*COL2A1*	SNV	intron_variant	Het	Maternal	NM_001844.5: c.610-85G>A	N/A	COL2A1-related disorders	PP3_Supporting	1	Low
NNET1018	Trio	*RIMS1*	SNV	missense_variant	Het	Maternal	NM_014989.7: c.1340A>C	NP_055804.2: p.(His447Pro)	Cone-rod dystrophy 7	PM2_Moderate; BP4_Supporting	1	Low
NNET1018	Trio	*PHOX2B*	SNV	missense_variant	Het	Paternal	NM_003924.4: c.648_650dup	NP_003915.2: p.(Gly217dup)	Central hypoventilation syndrome, congenital, 1, with or without Hirschsprung disease	PM2_Moderate; BP4_Supporting	1	Low
NNET1025	Trio	*FRYL*	SNV	missense_variant	Het	Paternal	NM_015030.2: c.2254G>T	NP_055845.1: p.(Asp752Tyr)	Developmental delay, intellectual disability, and dysmorphic features	PM2_Moderate; BP4_Supporting	1	Low
NNET1025	Trio	*CNOT1*	SNV	missense_variant	Het	Paternal	NM_001265612.2: c.3746C>T	NP_001252541.1: p.(Pro1249Leu)	Vissers-Bodmer syndrome	BS2_Strong; PP2_Supporting	-3	Low
NNET1025	Trio	*ATP6V0C*	SNV	3_prime_UTR_variant	Het	De novo	NM_001198569.2: c.*136C>T	N/A	Epilepsy, early-onset, 3, with or without developmental delay	PS2_Supporting; BP7_Supporting	0	Low
NNET2002	Duo	*KIF1A*	SNV	missense_variant	Het	Unknown	NM_004321.8: c.3838C>A	NP_004312.2: p.(Pro1280Thr)	NESCAV syndrome	PP2_Supporting; PP3_Supporting	2	Mid
NNET2002	Duo	*COL12A1*	SNV	missense_variant	Het	Maternal	NM_004370.6: c.7988A>C	NP_004361.3: p.(Tyr2663Ser)	Bethlem myopathy 2	PM2_Moderate; BP1_Supporting; BP4_Supporting	0	Low
NNET2004	Trio	*DNMT1*	SNV	missense_variant	Het	Paternal	NM_001130823.3: c.925C>A	NP_001124295.1: p.(Leu309Ile)	Neuropathy, hereditary sensory, type IE	PM2_Moderate; BP4_Supporting	1	Low
NNET2005	Trio	*CACNA1C*	SNV	missense_variant	Het	Paternal	NM_000719.7: c.4885C>T	NP_000710.5: p.(Arg1629Trp)	Neurodevelopmental disorder with hypotonia, language delay, and skeletal defects with or without seizures	PM2_Moderate; PP2_Supporting; PP3_Supporting	4	High
NNET2005	Trio	*POGZ*	SNV	missense_variant	Het	Paternal	NM_015100.4: c.845C>T	NP_055915.2 p.(Thr282Met)	White-Sutton syndrome	PM2_Moderate; BP4_Supporting	1	Low
NNET2008	Trio	*ERMARD*	SNV	splice_donor_variant	Het	Paternal	NM_018341.3: c.1853+1G>A	N/A	Periventricular nodular heterotopia 6	N/A (Limited GDR)	0	Low
NNET2009	Duo	*PAK3*	SNV	intron_variant	Hemi	Maternal	NM_002578.5: c.175+4566T>G	N/A	Intellectual developmental disorder, X-linked 30	PM2_Moderate; BP7_Supporting	1	Low

NNET2010	Duo	*PPP3CA*	SNV	missense_variant	Het	Unknown	NM_000944.5: c.1528A>G	NP_000935.1: p.(Thr510Ala)	Developmental and epileptic encephalopathy 91	PM2_Moderate; PP2_Supporting; BP4_Supporting	2	Mid
NNET2011	Duo	*TNPO3*	SNV	missense_variant	Het	Unknown	NM_012470.4: c.1969C>T	NP_036602.1: p.(Arg657Trp)	Muscular dystrophy, limb-girdle, autosomal dominant 2	PM2_Moderate	2	Mid
NNET2011	Duo	*SPTBN2*	SNV	missense_variant	Het	Maternal	NM_006946.4: c.2476C>T	NP_008877.2: p.(Arg826Trp)	Spinocerebellar ataxia 5	PM2_Moderate	2	Mid
NNET2014	Duo	*NR4A2*	SNV	missense_variant	Het	Unknown	NM_006186.4: c.235T>C	NP_006177.1: p.(Tyr79His)	Neurodevelopmental disorder and epilepsy (NR4A2)	PM2_Moderate; PP2_Supporting; PP3_Supporting	3	Mid
NNET2018	Trio	*PIEZO2*	SNV	missense_variant	Het	Paternal	NM_022068.4: c.6223C>T	NP_071351.2: p.(Arg2075Cys)	Arthrogryposis, distal, with impaired proprioception and touch	PM2_Moderate; PP2_Supporting; BP4_Supporting	2	Mid
NNET2018	Trio	*PIEZO2*	SNV	missense_variant	Het	Maternal	NM_022068.4: c.3236A>G	NP_071351.2: p.(Tyr1079Cys)	Arthrogryposis, distal, with impaired proprioception and touch	PM2_Moderate; PP2_Supporting; BP4_Supporting	2	Mid
NNET2019	Trio	*PIEZO2*	SNV	missense_variant	Het	Maternal	NM_022068.4:c.5413G>A	NP_071351.2: p.(Glu1805Lys)	Arthrogryposis, distal, with impaired proprioception and touch	PM2_Moderate; BP4_Supporting	1	Low
NNET2019	Trio	*PIEZO2*	SNV	missense_variant	Het	Paternal	NM_022068.4: c.1249G>C	NP_071351.2: p.(Val417Leu)	Arthrogryposis, distal, with impaired proprioception and touch	PM2_Moderate; BP4_Supporting	1	Low
NNET2021	Trio	*GRID2*	SNV	missense_variant	Het	Maternal	NM_001510.4: c.1964T>G	NP_001501.2: p.(Phe655Cys)	Spinocerebellar ataxia, autosomal recessive 18	PM1_Moderate; PM2_Moderate; PP3_Supporting	5	High
NNET2021	Trio	*GRID2*	SNV	frameshift_variant	Het	Paternal	NM_001510.4: c.2834del	NP_001501.2: p.(Asp945ValfsTer12)	Spinocerebellar ataxia, autosomal recessive 18	PVS1_Moderate; PM2_Supporting	3	Mid
NNET2021	Trio	*FHL1*	SNV	missense_variant	Hemi	De novo	NM_001449.5: c.359C>T	NP_001440.2: p.(Thr120Ile)	Uruguay faciocardiomusculoskeletal syndrome	PM2_Moderate; BP4_Supporting	1	Low
NNET2024	Trio	*HUWE1*	SNV	intron_variant	Hemi	Maternal	NM_031407.7: c.2877-11T>G	N/A	Intellectual developmental disorder, X-linked syndromic, Turner type	PM2_Moderate; PP3_Supporting; PP4_Supporting	4	High
NNET2026	Trio	*ATL1*	SNV	missense_variant	Het	Paternal	NM_015915.5: c.1259A>G	NP_056999.2: p.(Gln420Arg)	Spastic paraplegia 3A, autosomal dominant	PM1_Moderate; PM2_Moderate; PP2_Supporting; BP4_Supporting	4	High
NNET3002	Trio	*COL4A1*	SNV	intron_variant	Het	Maternal	NM_001845.6: c.652-14T>G	N/A	Brain small vessel disease with or without ocular anomalies	PM2_Moderate; PP3_Supporting; PP4_Supporting	4	High
NNET3002	Trio	*SPG7*	SNV	stop-gained	Het	Maternal	NM_001363850.1: c.2182C>T	NP_001350779.1: p.(Arg728Ter)	Spastic paraplegia 7	PVS1_Moderate; PM2_Moderate	4	High
NNET3007	Trio	*CAMTA1*	SNV	missense_variant	Het	Maternal	NM_015215.4: c.4129T>C	NP_056030.1: p.(Tyr1377His)	Cerebellar dysfunction with variable cognitive and behavioral abnormalities	PM2_Moderate; PP2_Supporting; BP4_Supporting	2	Mid
NNET3011	Quad	*ATP1A2*	SNV	missense_variant	Het	Maternal	NM_000702.4: c.2705A>T	NP_000693.1: p.(Glu902Val)	Migraine, familial basilar	PM2_Moderate; PP3_Supporting	3	Mid

NNET3011	Quad	*SLITRK2*	SNV	missense_variant	Hemi	Maternal	NM_032539.5: c.238A>G	NP_115928.1: p.(Asn80Asp)	Intellectual developmental disorder, X-linked 111	PM2_Moderate; BP4_Supporting	1	Low
NNET3012	Quad	*ATP1A2*	SNV	missense_variant	Het	Maternal	NM_000702.4: c.2705A>T	NP_000693.1: p.(Glu902Val)	Migraine, familial basilar	PM2_Moderate; PP3_Supporting	3	Mid
NNET3013	Duo	*NAA15*	SNV	missense_variant	Het	Unknown	NM_057175.5: c.1646G>A	NP_476516.1: p.(Arg549Gln)	Intellectual developmental disorder, autosomal dominant 50, with behavioral abnormalities	PM1_Moderate; PM2_Moderate	4	High
NNET3013	Duo	*ATRX*	SNV	missense_variant	Hemi	Maternal	NM_000489.6: c.867G>C	NP_000480.3 p.(Gln289His)	Intellectual disability-hypotonic facies syndrome, X-linked	PM2_Moderate	2	Mid
NNET3013	Duo	*COL6A2*	SNV	missense_variant	Het	Maternal	NM_001849.4: c.484G>A	NP_001840.3: p.(Asp162Asn)	*COL6A2*-related disorder	PM2_Moderate; PP3_Supporting	3	Mid
NNET3020	Singleton	*FOXP1*	SNV	intron_variant	Het	Unknown	NM_032682.6: c.-438A>G	N/A	Intellectual developmental disorder with language impairment with or without autistic features	PM2_Moderate; PP3_Supporting	3	Mid
NNET3023	Duo	*CACNA1G*	SNV	intron_variant	Het	Unknown	NM_018896.5: c.4760-1224G>A	N/A	Spinocerebellar ataxia 42	PM2_Moderate; PP3_Supporting	3	Mid
NNET4008	Trio	*PMP22*	SNV	missense_variant	Het	Paternal	NM_000304.4: c.353C>T	NP_000295: p.(Thr118Met)	Charcot-Marie-Tooth disease, type 1E	PS3_Moderate; (PS4_Moderate); (PM3_Strong); PP2; PP3	4	High

SNV = single nucleotide variant; Het = heterozygous; Hemi= Hemizygous; VUS = variant of uncertain significance. Points = points awarded for sub-classification of VUS variants per Durkie et al. (2024). VUS sub-classification = VUS sub-classification based on Figure 3 Durkie et al. (2024).

Supplementary Table 7: Phenotypic details reported in individuals with a P/LP/VOI variant

Case	Gene	HGVSC / ISCN	HGVSP	Classification	Disorder	Proband phenotypes	Antental/Neonatal/Postnatal risk factors
NNET1005	*PMP22*	NC_[000017.11]: g.14159934_15527543dup	N/A	P	Hereditary Motor and Sensory Neuropathy Type 1A/Charcot Marie-Tooth Type 1A	Visual impairment; Respiratory failure requiring assisted ventilation; Premature birth; Spastic diplegia; Spasticity; Tip-toe gait; Cataract; Caesarian section; Pregnancy history; Broad-based gait; Unsteady gait	Respiratory failure requiring assisted ventilation; Premature birth; Caesarean section
NNET1005	*EPHA2*	NM_004431.5: c.2826-9G>A	N/A	P	*EPHA2*-related cataract	See above	See above
NNET1017	*MT-ATP6*	ENST00000361899.2: c.467T>G	p.(Leu156Arg)	P	*MT-ATP6*-related mitochondrial disease	Seizure; Specific learning disability; Spastic hemiparesis; Left hemiplegia; Visual impairment; Spasticity; Hypermetropia; Ischemic stroke	

NNET1019	*F11*	NM_000128.4: c.901T>C	NP_000119.1 :p.(Phe301Leu)	P	Factor XI deficiency	Spastic diplegia; Spasticity; Periventricular leukomalacia	
NNET1020	*CIC*	NM_015125.5: c.3640C>T	NP_055940.3: p.(Arg1214Ter)	P	*CIC*-related disorder	Intellectual disability; Dystonia; Spastic tetraplegia; Visual impairment; Caesarean section; Spasticity; Bilateral tonic-clonic seizure; Seizure; Astigmatism; Scoliosis; Tube feeding; Progressive microcephaly; Neurodevelopmental delay; Esotropia; Encephalopathy; Patent foramen ovale; Neonatal seizure; Heart murmur	Respiratory failure requiring assisted ventilation; Infectious encephalitis; Neonatal asphyxia; Hypoxemia;
NNET2019	*SCN1A*	NM_001165963.4: c.5494G>C	NP_001159435.1: p.(Ala1832Pro)	LP	*SCN1A*-related disorder	Generalized non-motor (absence) seizure; Abnormality of the musculature; Abnormality of the nervous system; Abnormality of the digestive system; Intellectual disability; Delayed speech and language development ;Caesarean section; Dystonia; Motor delay; Global developmental delay; Focal aware seizure; Bilateral tonic-clonic seizure; Neurodevelopmental delay; Seizure; Infantile onset; Bilateral tonic-clonic seizure with generalized onset; Bilateral tonic-clonic seizure with focal onset; Complex febrile seizure; Status epilepticus; Encephalopathy; Cerebral atrophy; Ventriculomegaly; Exodeviation; Severe global developmental delay; Hemidystonia; Poor head control; Postural instability; Rigidity; Cerebral visual impairment; Limb dystonia; Hip dysplasia; Thoracic scoliosis; Constipation; Hypsarrhythmia; Bilateral multifocal epileptiform discharges; Myoclonic seizure; Focal tonic seizure	Infectious encephalitis; Meningitis; Caesarean section
NNET2020	*KRIT1*	NM_194456.1: c.397dup	NP_919438.1: p.(Tyr133LeufsTer11)	P	Cerebral cavernous malformations-1	Spasticity; Autistic behaviour; Generalized non-motor (absence) seizure; Intellectual disability; Atonic seizure; Spastic diplegia; Intellectual disability; Focal aware seizure; Seizure; Torticollis; Infantile onset; Generalized non-motor (absence) seizure; Hamstring contractures; Absent speech; Aggressive behaviour; Bilateral tonic-clonic seizure with focal onset; Global developmental delay; Head-banging; Intellectual disability, severe; Recurrent tonsillitis; Motor delay; Status epilepticus; Periventricular leukomalacia; Focal	Abnormality of prenatal development or birth; Pregnancy history (bleeding, low-lying placenta, threatened premature birth at 22 weeks);

						impaired awareness motor seizure; Joint hypermobility; Autism; Quadriceps muscle weakness; Focal aware seizure; Crouch gait	
NNET3008	*SETD1A*	NM_014712.3: c.2944G>T	NP_055527.1: p.(Glu982Ter)	P	*SETD1A*-related disorder	Spasticity; Spastic hemiparesis; Right hemiplegia; Intellectual disability; Intellectual disability, mild; Seizure; Bilateral tonic-clonic seizure; Visual impairment; Strabismus; Hemiparesis; Hemianopia; Periventricular leukomalacia; Neonatal seizure; Eczematoid dermatitis; Specific learning disability	Preterm intraventricular haemorrhage; Decreased foetal movement; Caesarean section; Infectious encephalitis
NNET3023	*TUBB2B*	NM_178012.5: c.1034T>A	NP_821080.1: p.(Ile345Asn)	LP	*TUBB2B*-related disorder	Spasticity; Spastic diplegia; Neurodevelopmental delay; Intellectual disability; Delayed speech and language development; Delayed early-childhood social milestone development; Autistic behaviour; Attention deficit hyperactivity disorder; Autism; Intellectual disability; Drooling; Short attention span; Delayed speech and language development; Urinary incontinence; Bowel incontinence; Lower limb hyperreflexia; abnormal brain MRI	
NNET3025	*ATL1*	NM_015915.5: c.1246C>T	NP_056999.2: p.(Arg416Cys)	P	*ATL1*-related spastic paraplegia	Spasticity; Spastic diplegia; Caesarean section; Lower limb spasticity; Pes planus	Caesarean section
NNET4012	*COL4A1*	NM_001845.6: c.3958G>A	NP_001836.3: p.(Gly1320Ser)	LP	*COL4A1*-related disorder	Spasticity; Spastic hemiparesis; Right hemiplegia; Neurodevelopmental delay; Intellectual disability; Global developmental delay; Intellectual disability, mild; Visual impairment; Global developmental delay; Abnormal corpus callosum morphology; Periventricular leukomalacia; Leukodystrophy; Drooling; Recurrent lower respiratory tract infections ;Hypermetropia	Premature birth; Moderate to late preterm birth
NNET4013	*TREX1*	NM_033629.6: c.490C>T / c.764_765dup	NP_338599.1: p.(Arg164Ter)/ p.(Thr256ProfsTer22)	P / LP	Aicardi-Goutières syndrome 1	Spasticity; Spastic tetraplegia; Intellectual disability; Intellectual disability, profound; Abnormality of vision; Abnormality of the eye; Spastic tetraplegia; Drooling; Hip dislocation; Abnormal posturing; Dysphagia; Progressive spasticity	

NNET1006	*ADAR*	NM_001111.5: c.577C>G	NP_001102.3 p.(Pro193Ala)	LP	Aicardi-Goutières syndrome	Visual impairment; Spastic tetraplegia; Spasticity; Athetosis; Dystonia; Sleep abnormality; Microcephaly; Intellectual disability, mild; Hypermetropia	Respiratory failure requiring assisted ventilation; Premature birth
NNET1010	*VWF*	NM_000552.5: c.2561G>A	NP_000543.3 p.(Arg854Gln)	P	von Willebrand disease type 2N	Spasticity; Spastic hemiparesis; Right hemiplegia; Cerebral infarct	Caesarean section; Respiratory failure requiring assisted ventilation
NNET2010	*NIPA1;NIPA2*	NC_000015.9: g.22648948_23124083del	N/A	P	15q11.2 microdeletion	Abnormality of the skeletal system; Ataxia; Behavioural abnormality; Spasticity; Intellectual disability; Spastic tetraplegia; Chronic lung disease; Inguinal hernia; Global developmental delay; Diminished ability to concentrate; Hyperactivity; Compensatory scoliosis; Chronic constipation; Strabismus; Abnormality of refraction; Spastic ataxia; Delayed ability to sit; Delayed ability to walk; Delayed fine motor development; Failure to thrive; Scissor gait; Upper limb hypertonia; Lower limb hypertonia; Spastic hemiparesis; Knee pain	Premature birth; Respiratory failure requiring assisted ventilation; Neonatal respiratory distress; Patent ductus arteriosus after premature birth;
NNET2015	*F2*	NM_000506.5: c.*97G>A	N/A	P	Cerebral infarction, susceptibility to	Neurodevelopmental delay; Behavioural abnormality; Spastic diplegia; Motor delay; Seizure; Delayed early-childhood social milestone development; Dyskinesia; Astigmatism; Pica; Meningitis; Hydrocele testis; Delayed ability to sit; Gait ataxia; Lower limb hypertonia; Autistic behaviour; Generalized non-motor (absence) seizure; Delayed ability to crawl; Dystonia; Hyperactive patellar reflex; Global developmental delay; Abnormality of the ear; Intellectual disability ;Delayed speech and language development; Bilateral tonic-clonic seizure; Focal-onset seizure; Ataxia; Intellectual disability; Profound sensorineural hearing impairment; Delayed ability to walk; Generalized atonic seizure; Frequent falls; Constipation; Tube feeding; Atonic seizure; Bilateral sensorineural hearing impairment; Insomnia	Respiratory failure requiring assisted ventilation; Subdural haemorrhage; Cerebral haemorrhage; Neonatal sepsis; Neonatal seizure;
NNET3004	*POLG*	NM_002693.3: c.2209G>C	NP_002684.1: p.(Gly737Arg)	P	Primary mitochondrial disease	Global developmental delay; Intellectual disability; Neurodevelopmental delay; Spasticity; Motor delay; Dystonia; Abnormality of the digestive	Prenatal maternal abnormality

						system; Strabismus; Spastic tetraplegia; Periventricular leukomalacia; Behavioral abnormality; Delayed speech and language development; Spastic tetraplegia; Dystonia; Spasticity; Periventricular leukomalacia; Hyperintensity of cerebral white matter on MRI; Constipation; Feeding difficulties; Abnormal communication; Exotropia	
NNET3024	*PAX8*	NM_003466.4: c.936dup	NP_003457.1: p.(Glu313ArgfsTer6)	LP	Hypothyroidism, congenital, due to thyroid dysgenesis or hypoplasia	Dystonia; Spasticity; Spastic tetraplegia; Neurodevelopmental delay; Intellectual disability; Delayed speech and language development; Motor delay; Global developmental delay; Delayed early-childhood social milestone development; Intellectual disability; Hearing impairment; Abnormality of the digestive system; Chronic lung disease; Bilateral sensorineural hearing impairment; Episodic vomiting; Cerebral palsy; Dystonia; Dyskinesia; Intellectual disability; Gastroesophageal reflux; Inguinal hernia	Premature birth; Respiratory failure requiring assisted ventilation; Extremely preterm birth
NNET4003	*ARFGEF1*	NM_006421.5: c.1441A>T	NP_006412.2 p.Ile481Phe	VUS	Developmental delay, impaired speech, and behavioral abnormalities, with or without seizures	Spasticity; Spastic diplegia; Intellectual disability ;Intellectual disability, mild; Feeding difficulties in infancy; Spastic diplegia; Constipation; Recurrent tonsillitis; Sensory behavioural abnormality; Iron deficiency anaemia; Autism; Short attention span; Hyperactivity;	
NNET4006	*COL11A1*	NM_001854.4:c.1245+1G>A	N/A	LP	Stickler syndrome	Spasticity; Spastic hemiparesis; Right hemiplegia; Neurodevelopmental delay; Motor delay; Delayed early-childhood social milestone development; Autistic behaviour; Abnormal brain morphology; Visual impairment; Right hemiplegia; Cerebral palsy; Spasticity; Middle cerebral artery stroke; Chiari type I malformation; Abnormal social behaviour; Autism;	Premature birth; Moderate to late preterm birth

Supplementary Table 8: Clinical correlation of pathogenic / likely pathogenic variants identified in the cohort. Association with neuromotor, neurodevelopmental (ND), behavioural and additional phenotypes discussed.

									Clinical correlation of phenotypes from OMIM / literature with proband's phenotype						
**NNET ID**	**Gene**	**Variant (c.)**	**Variant (p.)**	**P/LP**	**Disorder**	**Proband phenotypes**	**Antenatal/ Neonatal/ Postnatal risk factors**	**Notes**	**Neuromotor presentations**	**Relation to neuromotor phenotype (Full / Partial / Uncertain)**	**ND/ Behavioural phenotypes**	**Relation to ND phenotype (Full / Partial / Uncertain)**	**Additional phenotypes**	**Relation to additional phenotypes (Full / Partial / Uncertain)**	**Diagnostic results returned through GLH?**
NNET 1005	*PMP22*<br><br>*EPHA2* *	17-14195019-15279655 dup<br><br>c.2826-9G>A	N/A	P<br><br>P	Charcot-Marie-Tooth<br><br>Congenital cataract	Visual impairment; Respiratory failure requiring assisted ventilation;Premature birth; Spastic diplegia; Spasticity; Tip-toe gait; Cataract; Caesarian section; Pregnancy history; Broad-based gait; Unsteady gait	Respiratory failure requiring assisted ventilation; Premature birth; Caesarian section		Spastic diplegia; Spasticity; Tip-toe gait; Broad-based gait;	Partial	N/A	N/A	Visual impairment; Cataract	Full	Yes
NNET 1017	*MT-ATP6**	MT:8993 T>G, c.467T>G	p.(Leu156Arg)	P	Primary mitochondrial disease	Seizure; Specific learning disability; Spastic hemiparesis; Left hemiplegia; Visual impairment; Spasticity; Hypermetropia; Ischemic stroke	Ischemic stroke		Spastic hemiparesis; Left hemiplegia; Spasticity	Uncertain	Specific learning disability;	Partial	Seizure; Visual impairment; Hypermetropia; Ischemic stroke	Partial	Yes
NNET 1019	*F11*	c.901T>C	p.(Phe301Leu)	P	Factor XI deficiency	Spastic diplegia; Spasticity; Periventricular leukomalacia			Spastic diplegia; Spasticity; Periventricular leukomalacia	Uncertain	N/A	N/A	N/A	N/A	Yes
NNET 2019	*SCN1A*	c.5461G>C	p.(Ala1821Pro)	LP	*SCN1A*-related disorders	Generalized non-motor (absence) seizure; Abnormality of the musculature; Abnormality of the nervous system; Abnormality of the digestive system; Meningitis; Intellectual disability; Delayed speech and language development; Caesarian section; Dystonia; Motor delay; Global developmental delay; Focal aware seizure; Bilateral tonic-clonic seizure; Neurodevelopmental delay; Seizure; Infantile onset; Bilateral tonic-clonic seizure with generalized onset ; Bilateral tonic-clonic seizure with focal onset; Complex febrile seizure; Status epilepticus; Infectious encephalitis; Encephalopathy; Cerebral atrophy; Ventriculomegaly; Exodeviation; Severe global developmental delay; Hemidystonia; Poor head control; Postural instability; Rigidity; Cerebral visual impairment; Limb dystonia; Hip dysplasia; Thoracic scoliosis; Constipation; Hypsarrhythmia; Bilateral multifocal epileptiform	Infectious encephalitis; Meningitis; Caesarian section	Cerebral insult from infectious encephalitis could have explained GDD and seizures.	Dystonia; Cerebral atrophy; Ventriculomegaly; Hemidystonia; Poor head control; Postural instability; Rigidity; Limb dystonia; Hip dysplasia; Encephalopathy	Uncertain	Intellectual disability; Delayed speech and language development; Motor delay; Severe global developmental delay; Neurodevelopmental delay;	Full	Generalized non-motor (absence) seizure; Abnormality of the musculature; Abnormality of the nervous system; Abnormality of the digestive system; Focal aware seizure; Bilateral tonic-clonic seizure with generalized onset; Seizure, infantile onset; Bilateral tonic-clonic seizure with focal onset; Complex febrile seizure; Status epilepticus; Exodeviation; Cerebral visual impairment; Thoracic scoliosis; Constipation; Hypsarrhythmia; Bilateral multifocal epileptiform discharges; Myoclonic seizure; Focal tonic seizure;	Full	Yes

						discharges;Myoclonic seizure;Focal tonic seizure									
NNET 2020	*KRIT1**	c.397dup	p.(Tyr133LeufsTer11)	P	Cerebral cavernous malformations-1	Spasticity;Autistic behavior;Generalized non-motor (absence) seizure;Abnormality of prenatal development or birth;Intellectual disability;Atonic seizure;Spastic diplegia;Intellectual disability;Pregnancy history;Focal aware seizure;Seizure;Torticollis;Infantile onset;Generalized non-motor (absence) seizure;Hamstring contractures;Absent speech;Aggressive behavior;Bilateral tonic-clonic seizure with focal onset;Global developmental delay;Head-banging;Intellectual disability, severe;Recurrent tonsillitis;Motor delay;Status epilepticus;Periventricular leukomalacia;Focal impaired awareness motor seizure;Joint hypermobility;Autism;Quadriceps muscle weakness;Focal aware seizure;Crouch gait	Abnormality of prenatal development or birth; Pregnancy history (bleeding, low-lying placenta,threatened premature birth at 22 weeks);		Spasticity; Spastic diplegia; Periventricular leukomalacia; Hamstring contractures; Quadriceps muscle weakness; Crouch gait;	Uncertain	Autistic behaviour; Generalized non-motor (absence) seizure; Intellectual disability; Atonic seizure; Focal aware seizure; Absent speech; Aggressive behavior; Bilateral tonic-clonic seizure with focal onset; Global developmental delay; Head-banging; Intellectual disability, severe; Motor delay; Status epilepticus; Focal impaired awareness motor seizure; Autism; Focal aware seizure;	Partial	Tortocollis; Recurrent tonsillitis; Joint hypermobility;	Uncertain	Yes
NNET 3025	*ATL1*	c.1246C>T	p.(Arg416Cys)	LP	Spastic paraplegia 3A, autosomal dominant	Spasticity;Spastic diplegia;Caesarian section;Lower limb spasticity;Pes planus	Caesarian section;	Emergency Caesarian section and received more than 'routine newborn care', however, no further information given.	Spasticity; Spastic diplegia; Lower limb spasticity;	Full	N/A	N/A	Pes planus;	Uncertain	Yes
NNET 4013	*TREX1*	c.490C>T<br><br>c.764_765dup	p.(Arg164Ter)<br><br>p.(Thr256ProfsTer22)	P<br><br>P	Aicardi-Goutières syndrome	Spasticity;Spastic tetraplegia;Intellectual disability;Intellectual disability, profound;Abnormality of vision;Abnormality of the eye;Spastic tetraplegia;Drooling;Hip dislocation;Abnormal posturing;Dysphagia;Progressive spasticity		Maternal pregnancy-induced hypertension.	Spasticity; Spastic tetraplegia; Hip dislocation; Abnormal posturing; Progressive spasticity;	Full	Intellectual disability, profound;	Full	Abnormality of vision; Abnormality of the eye; Drooling; Dysphagia;	Full	Yes<br><br>Yes
NNET 4012	*COL4A1*	c.3958G>A	p.Gly1320Ser	LP	*COL4A1*-related disorders	Spasticity;Spastic hemiparesis;Right hemiplegia;Neurodevelopmental delay;Intellectual disability;Global developmental delay;Intellectual disability, mild;Visual	Premature birth; Moderate to late preterm birth;		Spasticity; Spastic hemiparesis; Right hemiplegia; Abnormal corpus callosum morphology; Periventricular	Partial	Neurodevelopmental delay; Intellectual disability; Global development delay; Intellectual disability, mild;	Full	Visual impairment; Drooling; Recurrent lower respiratory tract infections; Hypermetropia;	Partial	Yes

						impairment;Premature birth;Moderate to late preterm birth;Global developmental delay;Abnormal corpus callosum morphology;Periventricular leukomalacia;Leukodystrophy;Drooling;Recurrent lower respiratory tract infections;Hypermetropia			leukomalacia; Leukodystrophy;						
NNET 3008	*SETD1 A**	c.2944G>T	p.(Glu982Ter)	LP	Neurodevelopmental disorder with speech impairment and dysmorphic facies	Spasticity;Spastic hemiparesis;Right hemiplegia;Intellectual disability;Intellectual disability, mild;Seizure;Bilateral tonic-clonic seizure;Infectious encephalitis;Visual impairment;Strabismus;Caesarian section;Hemiparesis;Hemianopia;Preterm intraventricular hemorrhage;Periventricular leukomalacia;Neonatal seizure;Eczematoid dermatitis;Specific learning disability;Decreased fetal movement	Preterm intraventricular hemorrhage; Decreased fetal movement; Caesarian section; Infectious encephalitis;		Spasticity; Spastic hemiparesis; Right hemiplegia; Hemiparesis; Periventricular leukomalacia;	Uncertain	Intellectual disability; Intellectual disability, mild; Specific learning disability;	Full	Seizure; Bilateral tonic-clonic seizure; Visual impairment; Strabismus; Hemianopia; Neonatal seizure; Eczematoid dermatitis;	Partial	Yes
NNET 3023	*TUBB2 B*	c.1034T>A	p.(Ile345Asn)	LP	(TUBB2B-related disorder) Cortical dysplasia, complex, with other brain malformations 7	Spasticity;Spastic diplegia;Neurodevelopmental delay;Intellectual disability;Delayed speech and language development;Delayed early-childhood social milestone development;Autistic behavior;Attention deficit hyperactivity disorder;Autism;Intellectual disability;Drooling;Short attention span;Delayed speech and language development;Urinary incontinence;Bowel incontinence;Lower limb hyperreflexia		MRI findings: There are extensive corticated and/gyration abnormalities bilaterally involving the sylvian cortex with extension into the frontal and parietal operculum and perirolandic regions bilaterally.<br><br>There is asymmetry of the lateral ventricles with the left being more prominent. No evidence of abnormal white matter signal change to suggest that there is a cystic PVL component.	Spasticity; Spastic diplegia; Lower limb hyperreflexia;	Uncertain	Neurodevelopmental delay; Intellectual disability; Delayed speech and language development; Delated early-childhood milestone development; Autistic behavior; Attention deficit hyperactivity disorder; Autism; Short attention span;	Full	Drooling; Urinary incontinence; Bowel incontinence;	Uncertain	Yes

NNET 1020	*CIC**	c.3640C>T	p.(Arg1214Ter)	LP	CIC-related disorder (Intellectual developmental disorder, autosomal dominant 45)	Intellectual disability;Dystonia;Spastic tetraplegia;Respiratory failure requiring assisted ventilation;Visual impairment;Caesarian section;Spasticity;Infectious encephalitis;Bilateral tonic-clonic seizure;Seizure;Astigmatism;Scoliosis;Tube feeding;Progressive microcephaly;Neurodevelopmental delay;Esotropia;Neonatal asphyxia;Encephalopathy;Hypoxemia;Patent foramen ovale;Neonatal seizure;Heart murmur	Respiratory failure requiring assisted ventilation; Infectious encephalitis; Neonatal asphyxia; Hypoxemia;		Dystonia; Spastic tetraplegia; Spasticity; Progressive microcephaly; Encephalopathy;	Uncertain	Intellectual disability; Neurodevelopmental delay;	Full	Visual impairment; Bilateral tonic-clonic seizure; Astigmatism; Scoliosis; Tube feeding; Esotropia; Patent foramen ovale; Neonatal seizure; Heart murmur;	Partial	Yes

Supplemental Methods

DNA Sequencing

Extracted DNA samples were quantified on a Gemini XPS Microplate Reader (Molecular Devices) using the Quant-iT™ PicoGreen® dsDNA Assay Kit.

Sequencing libraries from blood-extracted DNA samples were prepared using Illumina DNA PCR-Free Library Prep, Tagmentation protocol (Illumina DNA PCR-Free Library Prep Reference Guide, Illumina document #1000000086922 v03). Genomic DNA (350ng in 25ul) was fragmented and tagged with NGS adapter sequences by incubating with Bead-Linked Transposomes PCR-Free (BLT-PF). IDT index adapters were added during the Extension Ligation Mix (ELM) step. Libraries were cleaned up and double size selected. Final libraries were eluted in 40 µl of RSB.

All libraries were quantified using Kapa qPCR (Merck Life Science UK, Gillingham, UK) on a Roche LightCycler LC480 (Roche Diagnostics Limited, Burgess Hill, UK) and normalised to 1.25 nM. Normalised libraries were loaded on to S4 flowcells using the Illumina® NovaSeq 6000 Sequencing System Guide XP Worflow. S1, S2 or S4 flowcells were sequenced on Novaseq 6000 using two-channel sequencing chemistry and bidirectional scanning. Libraries were sequenced as paired 150 cycles to an average depth of >30x, following alignment and removal of duplicate read pairs.

Sample processing and QC

Sequence analysis included several key steps: Sequence QC and mapping, alignment, variant calling, annotation, filtering, and prioritisation of variants (see Tertiary analysis). A manifest data submission workflow (including family size and sample information, phenotype HPO terms, DNA sample ID, and clinical data) was developed and implemented on the cloud-based DNANexus platform (https://www.dnanexus.com/) and manifest files were submitted to Emedgene™ for variant analysis and interpretation.

For all samples, cleaned reads were aligned to a Human genome reference GRCh38 (version GCA_000001405.15) to generate variant call format (VCF) files for various variant types, including single nucleotide variants (SNVs), small insertions and deletions (indels), short tandem repeats (STRs), copy number variants (CNVs), structural variants (SVs), mobile element insertions (MEIs), uniparental heterodisomy and isodisomy (UPD), *SMN1/SMN2* copy numbers, and mitochondrial DNA (mtDNA) variants.

QC metrics of alignment and variant calling were analysed for each DRAGEN run. The genome was sequenced to 30-40X, with a QC threshold of 40X. To ensure that no sample mix-ups occurred throughout the workflow in the pedigree (duo/trio/quads), the relatedness coefficient percentage (ranging from 46% to 53%) of the proband with respect to the mother and father samples was considered. For the sample variant quality check, the ratios of base substitutions [transition/transversion (Ti/Tv)], and Het/Hom ratios, were examined which were found to be within the range of 1.98–2.0 and 1.2–2.0, respectively.

Secondary Analysis

Variants were called using the DRAGEN™ secondary analysis pipeline version 4.3. https://emea.illumina.com/content/dam/illumina/gcs/assembled-assets/marketing-literature/dragen-bio-it-data-sheet-m-gl-00680/dragen-bio-it-data-sheet-m-gl-00680.pdf

Tertiary Analysis

Variant analysis including filtering, prioritization and interpretation was performed using the Emedgene™ software, version 35, which uses explainable AI (XAI) to generate a shortlist of potential causative variants along with supporting evidence from the literature and curated databases.

In addition to the AI preset filter, a set of preset filters designed to match the filtering specified in the Genomics England Rare Disease Tiering Process for the analysis of rare disease (https://re-docs.genomicsengland.co.uk/tiering/) were used. These included filtering for rare small variants with severe consequences, all variant types with confirmed de novo occurrence, CNVs > 100 kb called by the depth of coverage caller, and CNVs > 100 kb that overlap known disease-associated genes. Gene-agnostic WGS analysis was performed independently by two scientists (Figure 1: first and second analysis) and focused on triage of all

variants prioritised by the AI and/or found in genes associated with diseases with potential phenotypic overlap in the preset filters described.

In addition to the gene-agnostic approach, a comprehensive list of 1503 genes reported to be associated with CP (the "CP gene list"; see Supplementary Table 1) was applied. This list was compiled from cerebral palsy gene lists available in the following databases and publications: 161 genes from the diagnostic grade "Green" genes from the Cerebral Palsy panel (Version 1.389), PanelApp Australia; 17 genes with a clinical validity classification of Definitive or Moderate from the ClinGen Cerebral Palsy Gene Curation Expert Panel (https://clinicalgenome.org/affiliation/40113/) ; 141 genes from Basu et al. (2024); 168 genes from van Eyk et al. (2021) and 208 genes from van Eyk et al. (2023); 1411 genes from Fehlings et al. (2024). Gene lists were combined, duplicated genes were removed and the remaining gene symbols checked against approved symbols using the Multi-symbol checker tool from HUGO Gene Nomenclature Committee (https://www.genenames.org/tools/multi-symbol-checker/) (see Supplementary Table 1).

Sub-classification of VUS

The 40 VUS SNVs identified in genes on the CP gene list in 25 cases were sub-classified into High, Medium and Low, estimated from a Bayesian approach and a point-scale derived from the Bayesian approach as described in Durkie et al. (2024). See Supplementary Table 4 for VUS subclassifications. Variants were awarded evidence points based on the quality and strength of the ACMG criteria assigned during variant curation. VUS variant sub-classifications are given in Supplementary Table 2. Participants were segregated according to the highest variant classification.

Phenotype-Based Genetic Diagnostic Model Pipeline

The models were built using all available HPO terms from this study, the Next Generation Children's study for individuals that met inclusion criteria with CP phenotypes, and from the Fehling et al. (2024) published dataset. The Genomics England (GEL) cerebral palsy cohort (GEL CP) was generated by screening the 100k Genomes Project National Genomic Research Library database on the GEL platform (version 18, n = 90,178). Participants who were recorded with a cerebral palsy term in at least one of the HPO term (HP:0100021, HP:0011445) and/or at least one of the International Classification of Diseases 10th Revision (ICD-10) code for CP (G80.0, G80.1, G80.2, G80.3, G80.4, G80.8, G80.9) in their medical history data were included in the cohort. The recorded HPO terms (by the recruiting clinician at the time of recruitment), filtered to include participants with 'Cerebral palsy' (HP:0100021), and diagnosis status were extracted per participant for analysis. Obsolete HPO terms were manually updated using the Monarch Initiative website (https://monarchinitiative.org/).

A binary classification method was developed to predict genetic diagnostic outcomes from patient phenotypic data. All participants included in the final analysis possessed complete genomic data and diagnostic outcome classifications, ensuring no missing values existed among the fundamental training and validation outcome variables. Phenotypic clinical data were converted into a sparse binary matrix of HPO terms, where explicitly documented phenotypic abnormalities were coded as 1 and unrecorded clinical features were coded as 0. This approach reflects established computational phenotyping paradigms based on positive symptom identification, where unrecorded terms signify a lack of clinical observation rather than traditional missing data artifacts[1,2]. Consequently, standard multiple imputation techniques were considered conceptually inappropriate and irrelevant for these clinical data matrices. This predictive classification framework integrates Principal Component Analysis (PCA) for data dimensionality reduction with a supervised Linear Discriminant Analysis (LDA) classification model. The complete analysis was implemented in R (v4.5.0). This approach has been previously used for classifying schizophrenic[3,4], neurodegenerative disease[5], and control participants.

Step 1: Prepare Input Data

The model requires two data inputs: a binary matrix of Human Phenotype Ontology (HPO) terms and a binary vector indicating diagnostic status.

- *Diagnostic Status Vector*: Cases were categorized into a positive (diagnostic) group and a negative (non-diagnostic) group.
  - *Positive Group*: Included cases with 'Reported', 'VOI', and 'High VUS' results.
  - *Negative Group*: included cases with 'Low VUS' and 'Non-diagnostic' results.
- *Binary HPO Matrix*: To ensure standardized annotation depth, all HPO terms were normalized using the 'ontologyIndex' R package. HPO terms were carefully curated for consistency, and any obsolete terms were updated to their current valid equivalents. All curated terms were retained for downstream analysis. This process resulted in a binary matrix representing the presence or absence of normalized HPO terms for each case (rows) and HPO term (columns).

Step 2: Optimize Number of Principal Components (PCs) via bootstrapping
To find the optimal number of PCs for the model, a bootstrapping procedure is performed by iterating over a candidate range of PC counts. For each candidate PC count, 100 bootstrapped iterations are run as follows:

- *Sample Data*: 70% of the data is sampled for training, ensuring a balanced class distribution is maintained.
- *Apply PCA*: PCA is performed on the binary HPO term matrix of the training sample using the 'prcomp' function, without centering or scaling the data.
- *Train LDA Model*: An LDA model is trained on the PCA-transformed training data. The model is built to generate a classification probability vector classifying cases into their predefined diagnostic groups using the 'lda' function from the 'MASS' package (v2.2.2) with default parameters.
  OR
- *Train GBM Model:* Model training was performed using the 'gbm' (v2.2.2) package, and accuracy was used as the evaluation metric for model performance. The following parameters were specified within 'tuneGrid' option of the 'train' function: 'n.trees' = 100, 'interaction.depth' = 3, 'shrinkage' = 0.05, and 'n.minobsinnode' = 10. The remaining options and parameters were left at their default settings.)
- *Predict and Evaluate:* The complete dataset is projected into the PCA space derived from the training sample. The trained LDA model is then used to predict the genetic diagnostic status for the full dataset.
- *Store Results:* The Area Under the Curve (AUC) for the iteration is calculated from the Receiver Operating Characteristic (ROC) curve using the 'roc' function from the 'pROC' package (v1.18.5). The AUC and the PCA + LDA model from the iteration are stored.

Step 3: Select Optimal Number of PCs
The best PC count is identified by calculating the average AUC across the 100 iterations for each tested PC count. The number of PCs that yields the highest average AUC is selected as the optimal number.
Step 4: Select Final Model
From the 100 models trained using the optimal number of PCs (determined in Step 3), the single model whose AUC is closest to the mean AUC of that set is selected as the final model.
Step 5: Determine Best Probability Threshold
Using the predictions from the final model selected in Step 4, the operational probability threshold is explicitly identified. Rather than relying on standard mathematical indices that weight false positives and false negatives equally (such as the standard Youden's J statistic), this operational threshold is explicitly selected to prioritize high diagnostic sensitivity to avoid missing true genetic diagnoses.
Step 6: Final Output
This pipeline generates the final, optimized components for the PCA + LDA genetic diagnostic model:

- Optimal number of PCs
- Selected PCA+LDA model
- LDA scores
- Optimal classification probability threshold

HPO Term Contribution Analysis Pipeline

The contribution of normalized HPO terms to the model's predictions was assessed to determine the influence of individual clinical features. This protocol describes the specific methodology for calculating this contribution.
Step 1: Define Input Data
The following data and a pre-trained model are required for the analysis:

- *Normalised HPO terms matrix*: A binary matrix, HPO_terms, where rows represent individual cases and columns represent normalized HPO terms. The values within the matrix indicate the presence or absence of a specific term for each case.
- *Trained PCA + LDA model*: The combined PCA and LDA model trained for classification.

Step 2: Initialize Change Matrix
This step creates a matrix to store the results of the analysis.

- A change_matrix is generated with dimensions corresponding to the input data.
- All values in this matrix are initialized to 0. It will store the calculated difference in prediction probabilities.

Step 3: Iterative Contribution Calculation
This step systematically calculates the change in the model's prediction probability by removing one HPO term at a time for each case.

- *Outer Loop*: For each HPO term j:
- *Inner Loop*: For each case i:
  - Check for term presence: If HPO_terms[i, j] == 1, proceed with the following steps. Otherwise, skip to the next case.

- *Get original classification probability*:
  - Apply the PCA + LDA model to the original HPO term vector for case i (HPO_terms[i, :]).
  - The resulting prediction probability is stored as the original probability (oProb_matrix[i, j]).
- *Create modified case*:
  - Create a temporary copy of the case's HPO term vector, HPO_terms_modified[i, :].
  - In this copy, set the value for term j to 0 (HPO_terms_modified[i, j] = 0).
- *Get Modified Classification Probability*:
  - Apply the PCA + LDA model to the HPO_terms_modified [i, :] vector.
  - The resulting prediction probability is stored as the modified probability (mProb_matrix[i, j]).
- *Calculate classification probability change*:
  - Store the difference between the original and modified probabilities in change_matrix[i, j] = oProb_matrix[i, j] - mProb_matrix[i, j].

Step 4: Generate Final Output
The result of this pipeline is an aggregated score for each HPO term, representing its average influence on the model's output.

- For each HPO term j, calculate the average contribution by taking the mean of all values in change_matrix[:, j] where the term was originally present (HPO_terms[i, j] == 1).
- Calculate the respective 95% confidence intervals for each average contribution score.

Dimensionality Tuning Sensitivity and Global Effect Size Analysis
The optimal number of principal components was selected based entirely on an empirical validation paradigm maximizing the validation area under the receiver operating characteristic curve (AUC) across the 100 resampling iterations used for principal component optimization. Rather than utilizing null-hypothesis significance testing to determine model architecture, a global non-parametric sensitivity analysis was conducted to establish whether the classifier's performance was meaningfully sensitive to this parameterization. A global Kruskal-Wallis test was executed across the complete evaluation matrix of 7,600 AUC scores computed exclusively on the validation dataset. This matrix represented the 100 resampling iterations used for principal component optimization evaluated across 76 distinct principal component dimensions, utilizing PCA+LDA models trained on the training splits of the NNET+NGC+Fehlings'24 cohorts. The empirical Epsilon-squared (ϵ^2) effect size was derived from the resulting Kruskal-Wallis test statistic to isolate and quantify the magnitude of the dimensional effect on validation model discrimination, where an ϵ^2 value between $0.01 \leq \epsilon^2 < 0.06$ denotes a small effect, $0.06 \leq \epsilon^2 < 0.14$ denotes a medium effect, and $\epsilon^2 \geq 0.14$ indicates a large practical effect size[6]. To establish the statistical stability and precision of this estimate, a 1,000-iteration percentile bootstrap resampling loop was subsequently executed to establish the 95% confidence interval for the global Epsilon-squared effect size, mathematically validating the parameterization effect.

Model Calibration Analysis
Using the optimized PCA+LDA model parameters derived from the training dataset of the NNET+NGC+Fehlings'24 cohorts, posterior diagnostic probabilities were predicted for the validation dataset. Global model calibration and predictive accuracy were evaluated using two primary metrics. The observed-to-expected (O/E) ratio was calculated by dividing the total sum of observed positive outcomes by the sum of the predicted probabilities across the validation cohort. The Brier score was computed as the mean squared difference between the individual predicted probabilities and their corresponding observed binary outcomes. To assess calibration across varying levels of estimated risk, the predicted probabilities were ranked and partitioned into five equal-sized patient subgroups (quintiles). Within each discrete quintile, the mean predicted probability was calculated and evaluated against the actual observed proportion of positive diagnoses. The agreement between the predicted diagnostic risk and the observed outcome proportion was evaluated against a theoretical baseline of perfect calibration, represented by a diagonal identity line, to identify specific probability strata where the classifier systematically overestimated or underestimated clinical risk.

Data Processing and Net Benefit Computation
We predicted posterior diagnostic probabilities for the validation dataset using PCA+LDA parameters optimized on the NNET, NGC, and Fehlings'24 training cohorts. Performance and decision boundaries were evaluated using validation receiver operating characteristic (ROC) curves from the 100 resampling iterations. Rather than using a standard balanced Youden Index, the classification threshold was explicitly selected to prioritize clinical sensitivity, thereby minimizing false negatives. To ensure the study's utility for future meta-analyses, absolute classification counts, comprising true positives (TP), true negatives (TN), false positives (FP), and false negatives (FN) were computed at this sensitivity-optimized threshold.

Baseline prevalence was defined as the ratio of diagnosed cases to total individuals. Clinical Net Benefit was computed across threshold probabilities from 0 to 0.99 in 0.01 increments and evaluated over the clinically informative range of 0 to 0.80 to compare four distinct clinical strategies. The PCA+LDA Model strategy represents model-guided triage using threshold-specific true and false positive counts from the validation cohort. Its empirical 95% confidence ribbon was derived from the 2.5th and 97.5th percentiles of the Net Benefit distributions, calculated across the identical 100 resampling iterations used for principal component optimization to ensure strict statistical consistency. This model-guided approach was evaluated against baseline clinical strategies: 'Test All' (systematic referral), 'Test None' (a flat zero net benefit reference), and 'Perfect Model' (a theoretical classifier with 100% sensitivity and zero false positives, yielding a constant net benefit equal to the baseline prevalence).

References

1 Köhler, S., et al. 2021. The Human Phenotype Ontology in 2021. Nucleic Acids Research.

2 Bastarache, L., et al. 2018. Phenotype risk scores identify patients with unrecognized Mendelian disease. Science.

3 Sabeti M, Katebi SD, Boostani R, Price GW. 2011. A new approach for EEG signal classification of schizophrenic and control participants. Expert Systems with Applications 38(3):2063-2071.

4 Sabeti M, Katebi SD, Boostani R, Price GW. 2007. A new approach for EEG signal classification of schizophrenic and control participants. Expert Systems with Applications 38(3):2063-2071. Biomedical Signal Processing and Control 2(2):122-134.

5 Amin S, Singhal A. 2017. Identification and Classification of Neurodegenerative Diseases using Feature Selection through PCA-LD. 4th IEEE UTTAR PRADESH section International Conference on Electrical, Computer and Electronics 578-586.

6 Peres, F. F. 2026. Effect sizes for nonparametric tests. Biochemia Medica, 36(1), 010101. https://doi.org/10.11613/BM.2026.010101
